# Biofilm formation in enterococci: genotype-phenotype correlations and inhibition by vancomycin

**DOI:** 10.1038/s41598-017-05901-0

**Published:** 2017-07-18

**Authors:** Yomna A. Hashem, Heba M. Amin, Tamer M. Essam, Aymen S. Yassin, Ramy K. Aziz

**Affiliations:** 1 Department of Microbiology and Immunology, Faculty of Pharmacy, The British University in Egypt (BUE), Shorouk City, Egypt; 2 Department of Microbiology and Immunology, Faculty of Pharmacy, October University for Modern Sciences and Arts, 6 October City, Egypt; 3 Department of Microbiology and Immunology, Faculty of Pharmacy, Cairo University, Cairo, Egypt

## Abstract

Enterococci are nosocomial pathogens that can form biofilms, which contribute to their virulence and antibiotic resistance. Although many genes involved in biofilm formation have been defined, their distribution among enterococci has not been comprehensively studied on a genome scale, and their diagnostic ability to predict biofilm phenotypes is not fully established. Here, we assessed the biofilm-forming ability of 90 enterococcal clinical isolates. Major patterns of virulence gene distribution in enterococcal genomes were identified, and the differentiating virulence genes were screened by polymerase chain reaction (PCR) in 31 of the clinical isolates. We found that detection of *gelE* in *Enterococcus faecalis* is not sufficient to predict gelatinase activity unless *fsrAB*, or *fsrB* alone, is PCR-positive (*P = *0.0026 and 0.0012, respectively). We also found that *agg* is significantly enriched in isolates with medium and strong biofilm formation ability (*P = *0.0026). Additionally, vancomycin, applied at sub minimal inhibitory concentrations, inhibited biofilm in four out of five strong biofilm-forming isolates. In conclusion, we suggest using *agg* and *fsrB* genes, together with the previously established *gelE*, for better prediction of biofilm strength and gelatinase activity, respectively. Future studies should explore the mechanism of biofilm inhibition by vancomycin and its possible use for antivirulence therapy.

## Introduction

Enterococci are Gram-positive bacteria that cause serious nosocomial infections, including urinary tract, bloodstream infections and endocarditis^1^. Enterococci are known for their ability to form biofilms, which are populations of cells irreversibly attached to various biotic and abiotic surfaces and encased in a hydrated matrix of exopolymeric substances, proteins, polysaccharides and nucleic acids^2^. Biofilms contribute to bacterial virulence in several ways. For example, adherence, an early step in biofilm formation, allows the bacteria to bind to catheters (e.g., urinary and intravascular catheters), biliary stents and silicone gastrostomy devices^3^. Additionally, biofilms contribute to bacterial resistance to antibiotics^4^ and to phagocytosis, making their eradication extremely difficult^5^. In a mature biofilm, the bacterial cells can tolerate antibiotics at concentrations 10–1000 times higher than those required to kill planktonic cells^5^.

Several enterococcal virulence proteins have been studied for their important roles in biofilm development^6^. These include the aggregation substance (Agg), *Enterococcus f* *aecalis* endocarditis-associated antigen A (EfaA), adhesion of collagen of *E* *. faecalis* (Ace) and biofilm on plastic operon (Bop)^7^. Moreover, expression of pili on the cell surface, which facilitates cell adhesion, is considered the trigger of biofilm formation^8^, and pili components are encoded by the endocarditis- and biofilm-associated pili genetic locus (EbpABC) and an adjacent downstream sortase-encoding gene, *srt* ^9^.

In general, biofilm formation is associated with quorum sensing, which is the regulation of bacterial gene expression in response to large cell population densities. This regulation is usually driven by molecules known as autoinducers^10^. In *E. faecalis*, biofilm formation is regulated by the well-defined quorum sensing system, *fsr* (faecal streptococci regulator) locus^11^. This locus consists of three genes, *fsrA, fsrB* and *fsrC*, immediately located next to two virulence factor-encoding genes: one encoding a gelatinase (*gelE*) and the other a serine protease (*sprE*). The expression of *gelE* is regulated by the *fsr* locus^12^.

Enterococcal cells do not only communicate through Fsr quorum signaling, but are also capable of communicating by peptide pheromones, secreted by recipient cells to induce the conjugative apparatus of donor cell, which mediate the transfer of pheromone-responsive plasmids^13^. Some of these plasmids carry genes that regulate or promote biofilm formation, such as the plasmid-encoded aggregation substance genes^14^. A direct link between pheromones and biofilm formation was demonstrated in *Candida albicans* ^15^. Examples of secreted enterococcal pheromones are Cpd, Cob, and Ccf^16^.

Because of the role of biofilms in virulence and antimicrobial resistance, different methods have been developed for biofilm prevention and removal^17^. One such method is the use of sub-inhibitory concentrations of antibiotics, which was shown to modify the physicochemical properties and the architecture of the outer surface of enterococcal cells, thus affecting their overall virulence^1^. Sub-inhibitory antibiotic concentrations have also been used to inhibit bacterial initial adhesion to abiotic substrates^18^.

Additionally, despite a growing body of literature about the aforementioned genetic determinants involved in biofilm formation and regulation in enterococci, no clear genotype–phenotype correlation has been established for these determinants. Associations between some of these genes and biofilm formation have been recognized (e.g., *gelE* ^11, 19^); yet, no study has systematically investigated whether the complete or partial presence of this gene set is a predictor of the biofilm formation phenotype. Such correlation would be of great diagnostic value, but is hindered by poor metadata available about published enterococcal genomes, which impede attempts for comparative genomics between biofilm and non-biofilm formers.

For the above reasons we performed a comprehensive genome survey for biofilm-associated genes, with emphasis on those that could consistently predict the strength of biofilm-forming phenotype in *E. faecalis*. We examined biofilm formation and antibiotic resistance phenotypes in enterococcal clinical isolates; then we screened representative *E. faecalis* isolates for their biofilm-regulating quorum sensing genes, as well as other virulence genetic determinants. Finally, we investigated the effect of certain antibiotics applied at sub-inhibitory concentrations on biofilm formation in strong biofilm-forming isolates. We found that *agg* is a good predictor of biofilm strength, that the *fsrA* and *fsrB* genes have a high predictive value of biofilm-associated gelatinase activity, and that sub-minimal inhibitory concentrations of vancomycin were able to inhibit biofilm formation.

## Results

### Bacterial isolate identification and relatedness

Out of 140 clinical isolates, collected from Egyptian hospitals over a period of seven years and suspected to be enterococci, 90 isolates were identified as *Enterococcus* spp (Table S1). These showed small brownish-black colonies surrounded by a black zone when streaked on Enterococcosel agar. Under the microscope, they were Gram positive cocci or coccobacilli arranged in pairs and short chains. Further identification by catalase test and 6.5% NaCl tolerance showed that these enterococcal isolates were catalase negative and able to grow in high concentration of NaCl. Their cultivation on Chromogenic UTI agar showed blue colonies.

The isolates were further identified by the VITEK 2 identification system, and their majority were *E. faecalis* (65 isolates or 72.2%), with a good number of *E. faecium* (22 isolates or 24.4%). Two of the remaining isolates belonged to the species *E. casseliflavus* and only one was identified as *E. gallinarum* (Table S1).

**Figure 1:**
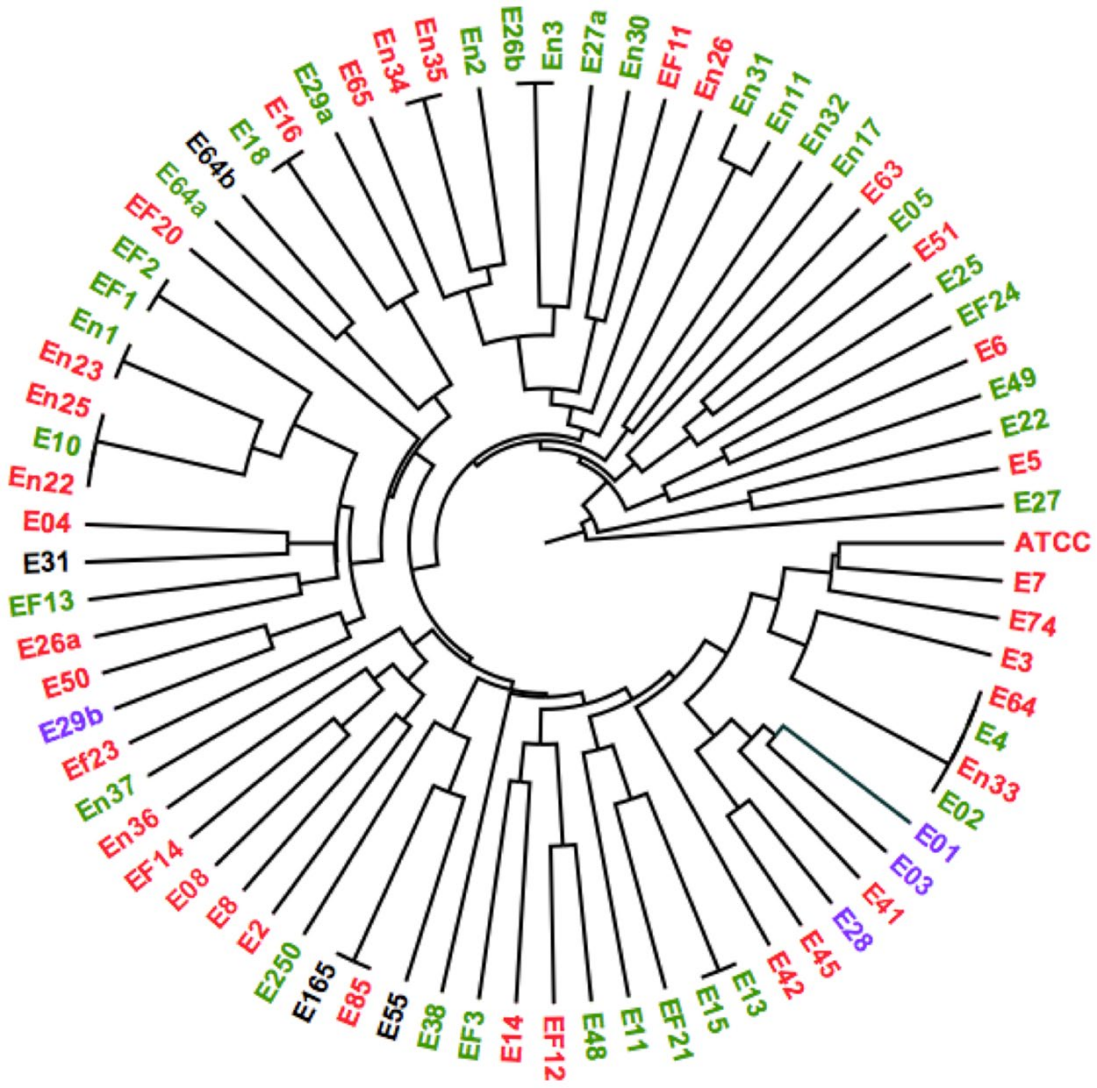
ERIC-PCR analysis of the isolates. An nweighted pair group method using arithmetic overage algorithm (UPGMA) dendrogram in a radial format representing the ERIC-PCR pattern relatedness of all tested isolates. Isolate names are color coded according to their biofilm strength (Purple, strong; Red, medium; Green, weak; Black, non-biofilm-forming).

The divergent nature of the isolates was confirmed by enterobacterial repetitive intergenic consensus polymerase chain reaction (ERIC-PCR), which showed a wide variety of patterns, most of which representing distinct enterococcal clades (Fig. 1 and S1). Only a few isolates appeared to be clonal or highly related (e.g., E02/E4/E64/En33 and E10/ En23/En25, Fig. 1).

### Phenotypic assessment of the biofilm-forming ability of the entercococcal clinical isolates

Congo-Red agar biofilm assay differentiated the 90 enterococcal isolates into (i) five strong biofilm-producing isolates; (ii) eighty-one isolates that varied in their biofilm formation strength between moderate and weak; and (iii) four non-biofilm-producing isolates. This assay could not persuasively differentiate between moderate and weak biofilm formation ability. On the other hand, the Crystal Violet biofilm assay differentiated isolates into strong, moderate, weak, and non-biofilm-forming according to the O.D. values at 545 nm. Again, only five of the isolates (5.5%) were classified as strong biofilm-formers; 38 isolates (42%) were moderate; 43 were weak biofilm-formers (48%); and four (4.5%) could not form any detectable biofilm.

Gelatinase activity has been described as one of the first steps in the process of biofilm formation^11^. Here, all isolates were screened for their *in vitro* gelatinase activity, but only 27% were gelatinase positive.

Of note, neither biofilm formation strength nor gelatinase activity correlated with ERIC-PCR types, except that three strong biofilm-forming strains fell in the same clade (E01, E03 and E28, Fig. 1). The ERIC patterns somehow correlated with the species of different isolates; however, given that only two major species were represented (*E. faecalis* and *E. faecium*), strain-to-strain variations remained important to determine by ERIC.

Biofilm formation strength was not significantly associated with the bacterial species (Chi-square *P* value = 0.2738), but there was obvious association (Chi-square *P* value ≤ 0.0001) with the sample source—urine and stool samples being enriched with biofilm-forming strains with higher average strength (Table 1).

**Table 1:** Association between biofilm-forming strength and specimen origin.

	Source							
	bile	blood	dental	pus	stool	urine	vagina	Total
**Biofilm Strength**								
No Biofilm	0	0	1	0	3	0	0	4
Weak	2	5	0	2	1	33	0	43
Moderate	0	0	2	0	5	31	0	38
Strong	0	0	0	0	0	4	1	5
Total	2	5	3	2	9	68	1	90

Chi-square = 58.03 (with 18 degrees of freedom); *P* ≤ 0.0001.

### Comparative genomic analysis

Biofilm formation in enterococci is a complex trait encoded by complementary, overlapping, and possibly redundant pathways/gene clusters; yet a clear biofilm genotype–phenotype correlation remains to be established. Here, a set of 17 protein-coding genes was selected after thorough literature survey (summarized in Introduction).

**Figure 2:**
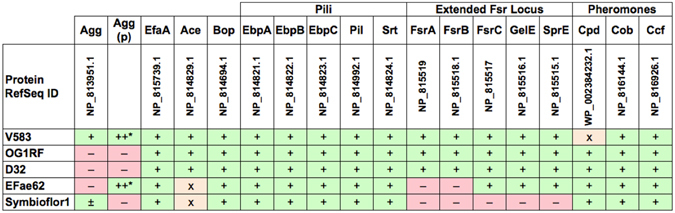
Comparative genomics screen for the 17 biofilm-associated protein-coding genes. Comparative genomics in a tabular format, representing patterns of gene presence and absence, analyzed by blastX against the 17 biofilm-related proteins and confirmed by blastN against primer pairs, in five representative *E. faecalis* strains. +: orthologous gene present; ±: paralogous gene present; −: gene absent; X: pseudogene. For Agg, more than one paralogous gene have been identified in some genomes. We differentiate the chromosomal and plasmid (p) copy of these genes.

First, we screened for the presence/absence pattern of these 17 protein-coding genes in five fully sequenced *E. faecalis* genomes (with a BlastX cutoff of either 80% amino acid identity or an E-value of 10^−80^). The genomes represented different biofilm phenotypes ranging from strong biofilm formers (V583) to the non-biofilm-forming probiotic strain Symbioflor1 (Fig. 2). Apart from the highly variable *ace* gene and the mobile *agg* gene, the variation was represented by the presence/absence of *fsr* locus as well as an inactivating mutation in the Cpd-encoding gene (Fig. 2). A more extensive analysis on the *fsr/gelE* locus was conducted by the Subsystems approach on the SEED server^20^, and showed many variants most of which are characterized by a missing *fsrAB*, or—less frequently—*fsrA*, whereas *gelE* and *fsrC* seemed to be the most conserved genes of the locus (Table S2 and http://pubseed.theseed.org/?page=SubsystemSelect; subsystem deposited under the name: “Biofilm Formation in Enterococci”).

**Figure 3:**
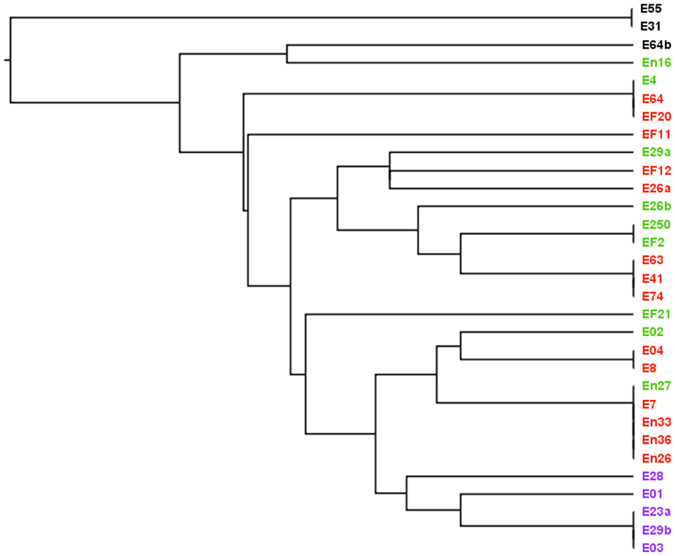
Results of PCR analysis of the distribution of each of the genes involved in biofilm formation.

We confirmed the aforementioned results by in silico PCR primer screening, using BLASTN searches for the primer pairs of the 17 genes mentioned above (Table 2) in the five representative genomes, and confirmed that Symbioflor1 lacked all members of the *fsr/gelE* operon, and that strain 62 lacked *fsrA* and *fsrB* but had *fsrC*.

**Table 2:** Specific primer pairs used in this study.

Gene	Forward primer	Reverse primer	Amplicon size (bp)
*agg*	TCTTGGACACGACCCATGAT	AGAAAGAACATCACCACGAGC	413
*efaA*	GACAGACCCTCACGAATATG	CCAGTTCATCATGCTGTAGTA	706
*ace*	GAATGACCGAGAACGATGGC	CTTGATGTTGGCCTGCTTCC	615
*bop*	GATCGTCTTCGCCATAGTAGG	ATACACAACAGCCCTTGGCT	312
*ebpA*	CCATTTGCAGAAGCAAGAATG	GAGTGAAAGTTCCTCCTCTAG	613
*ebpB*	CATTAGCAGAGGCATCGCAA	CAAGTGGTGGTAAGTCATAGG	504
*ebpC*	CTGCTACGAATATGGTGGTG	GGTGTTTGATTGTTTGCTTC	487
*pil*	GAAGAAACCAAAGCACCTAC	CTACCTAAGAAAAGAAACGCG	620
*srt*	GTATCCTTTTGTTAGCGATGC	TGTCCTCGAACTAATAACCGA	612
*fsrA*	CGTTCCGTCTCTCATAGTTA	GCAGGATTTGAGGTTGCTAA	474
*fsrB*	TAATCTAGGCTTAGTTCCCAC	CTAAATGGCTCTGTCGTCTAG	428
*fsrC*	GTGTTTTTGATTTCGCCAGAGA	TATAACAATCCCCAACCGTG	716
*gelE*	GGTGAAGAAGTTACTCTGAC	GGTATTGAGTTATGAGGGGC	704
*sprE*	CTGAGGACAGAAGACAAGAAG	GGTTTTTCTCACCTGGATAG	432
*cpd*	CGTTAGGCTTACATCAATCGAA	CCACCAACTACCCAGTAAAG	481
*cob*	GCTTTGTTTGCTGAATGTTCC	GACAACTGATGAGGTGCTAG	395
*ccf*	GGGAATTGAGTAGTGAAGAAG	AGCCGCTAAAATCGGTAAAAT	543

To more systematically determine which genes could serve as biomarkers for biofilm formation in *E. faecalis* in an unbiased way, we conducted genome-wide comparative analysis between biofilm-forming strains and the non-biofilm-forming Symbioflor 1. Genome-scale SEED comparative analysis confirmed BLAST results and, in addition, identified several prophage and pathogenicity island genes that differentiated the different strains (e.g., phage capsid proteins, integrases, transposases) (Table S2). Bidirectional best hit analysis indicated that the chromosomal *agg* gene of *E. faecalis* V583 is not the ortholog of the *agg* of *E. faecalis* Str. Symbioflor1, and that *agg* gene is more related to the *agg* variants carried on plasmids in *E. faecalis* V583 and 62 (Fig. 2 and Table S3).

Taken together, the above results indicate that *agg* and *ace* sequences are too variable to serve as good PCR biomarkers; on the other hand, *gelE* and the *fsr* locus genes could be more promising, notably *fsrA* and *fsrB*. Yet, the actual correlation of these candidate genes with biofilm phenotypes remained to be tested on clinical isolates.

### Screening clinical isolates for genes involved in biofilm formation

Out of the 90 enterococcal isolates, 31 were screened by PCR for the 17 genes listed above (Table 2), and the screening results are summarized as follows (Fig. 3 and Table 3): *srt, ccf, bop, efaA*, and *cpd* genes were present in 94% of the isolates. *gelE, sprE*, and *fsrC* genes in 90% of the isolates, *ebpC* and *pil* in 84%, *agg* in 81%, *ebpA* in 77%, *fsrB* in 65%, *ebpB* in 55%, *cob* in 50%, and *ace* and *fsrA* in 45% of the isolates.

**Table 3:** PCR screening results.

Isolate	Phenotype			Genotype																
	Biofilm OD	Biof. Str.	Gelatinase	*agg*	*efaA*	*ace*	*bop*	*epbA*	*ebpB*	*epbC*	*pil*	*srt*	*fsrA*	*fsrB*	*fsrC*	*gelE*	*sprE*	*cpd*	*cob*	*ccf*
E31	0.3	N	−	−	−	−	−	−	−	−	−	+	−	−	−	−	−	−	−	−
E55	0.3	N	−	−	−	−	−	−	−	−	−	+	−	−	−	−	−	−	−	−
E64b	0.3	N	−	−	+	−	−	+	−	+	+	+	−	−	−	−	−	+	+	+
E02	0.46	W	+	−	+	+	+	+	−	+	+	+	+	+	+	+	+	+	+	+
EF21	0.45	W	+	−	+	+	+	+	−	+	+	−	+	+	+	+	+	+	−	+
E250	0.42	W	+	+	+	+	+	+	+	+	+	+	−	+	+	+	+	+	+	+
E26b	0.6	W	+	−	+	+	+	+	+	+	+	+	−	+	+	+	+	+	+	+
E29a	0.54	W	+	+	+	−	+	+	+	+	+	+	−	−	+	+	+	+	−	+
En27	0.61	W	−	+	+	−	+	+	−	+	+	+	+	+	+	+	+	+	+	+
EF2	0.53	W	−	+	+	+	+	+	+	+	+	+	−	+	+	+	+	+	+	+
E4	0.4	W	−	+	+	−	+	−	+	−	+	+	−	−	+	+	+	+	−	+
En16	0.52	W	−	+	+	+	+	+	−	+	+	+	−	+	−	−	–	+	+	+
E04	0.77	M	+	+	+	+	+	+	−	+	+	+	+	+	+	+	+	+	+	+
E8	0.9	M	+	+	+	+	+	+	−	+	+	+	+	+	+	+	+	+	+	+
E7	0.89	M	+	+	+	−	+	+	−	+	+	+	+	+	+	+	+	+	+	+
En33	0.9	M	+	+	+	−	+	+	−	+	+	+	+	+	+	+	+	+	+	+
En36	0.91	M	+	+	+	−	+	+	−	+	+	+	+	+	+	+	+	+	+	+
E26a	1.06	M	+	+	+	−	+	+	+	+	−	+	−	+	+	+	+	+	−	+
En26	0.92	M	−	+	+	−	+	+	−	+	+	+	+	+	+	+	+	+	+	+
EF11	1.1	M	−	−	+	+	+	−	+	+	+	−	−	+	+	+	+	+	+	+
E63	1	M	−	+	+	+	+	+	+	+	+	+	−	−	+	+	+	+	+	+
E41	1.07	M	−	+	+	+	+	+	+	+	+	+	−	−	+	+	+	+	+	+
E74	1.1	M	−	+	+	+	+	+	+	+	+	+	−	−	+	+	+	+	+	+
EF12	0.93	M	−	+	+	+	+	+	+	+	−	+	−	−	+	+	+	+	−	+
E64	0.95	M	−	+	+	−	+	−	+	−	+	+	−	−	+	+	+	+	−	+
EF20	1	M	−	+	+	−	+	−	+	−	+	+	−	−	+	+	+	+	−	+
E29b	1.6	S	+	+	+	−	+	+	+	+	+	+	+	+	+	+	+	+	−	+
E03	1.63	S	+	+	+	−	+	+	+	+	+	+	+	+	+	+	+	+	−	+
E23a	162	S	−	+	+	−	+	+	+	+	+	+	+	+	+	+	+	+	−	+
E01	1.62	S	+	+	+	−	+	+	−	+	+	+	+	+	+	+	+	+	−	+
E28	1.6	S	+	+	+	−	+	−	−	+	+	+	+	+	+	+	+	+	−	+
Percent positive:			48	81	94	45	94	77	55	84	84	94	45	65	90	90	90	94	52	94
Percent negative:			52	19	6	55	6	23	45	16	16	6	55	35	10	10	10	6	48	6

Key: + , positive; −, negative; Biofilm formation strength (N, none; W, weak; M moderate, S, strong; Biof. Str. = Strength of biofilm.

These data are in agreement with the genome screen (Fig. 2) and subsytems analysis (Table S2), and indicate the importance of combining patterns of the *gelE/fsrA* locus genes in diagnosis and gelatinase activity prediction. Additionally, patterns of *agg*, *ace* combined with *gelE*/*fsrA* could distinguish weak biofilm phenotypes (Tables 4–5).

**Table 4:** Genotype–phenotype associations of *agg* PCR results and gelatinase activity with biofilm formation strength.+, positive; −, negative.

	*aggA presence*			Gelatinase activity		
	−	+	Total	−	+	Total
**Biofilm Strength**						
No Biofilm	3	0	3	3	0	3
Weak	3	6	9	4	5	9
Moderate	1	13	14	8	6	14
Strong	0	5	5	1	4	5
Total	7	24	31	16	15	31
	Chi-square = 14.25 *P* = 0.0026: significant association at *P* < *0.01*			Chi-square = 5.17 *P* = 0.1598: no significant association

**Table 5:** Genotype–phenotype associations of *gelA, fsrAB*, and *fsrB* with gelatinase activity with biofilm formation strength + , positive; −, negative; A: positive for *fsrA* only; A&B: positive for both *fsrA* and *fsrB*.

	*fsrAB*				*fsrB* only			*gelE*		
	−	*A*	*A* & *B*	Total	−	+	Total	−	+	Total
**Gelatinase Activity**										
−	10	3	3	16	10	6	16	4	12	16
**+**	1	3	11	15	1	14	15	0	15	15
Total	11	6	14	31	11	20	31	4	27	31
	Chi-square = 11.92 *P* = 0.0026 Significant at *P* < 0.01				Chi-square = 10.54 *P* = 0.0012 Significant at *P* < 0.01 Fisher’s Exact Test* = 0.002 (Significant)			Chi-square = 4.306 *P* = 0.038 Significant at *P* < *0.05* but not at *P* < *0.01* Fisher’s Exact Test* = 0.101 (Non-significant)		

*Fisher Exact Test applies only to 2 × 2 tables.

**Figure 4:**
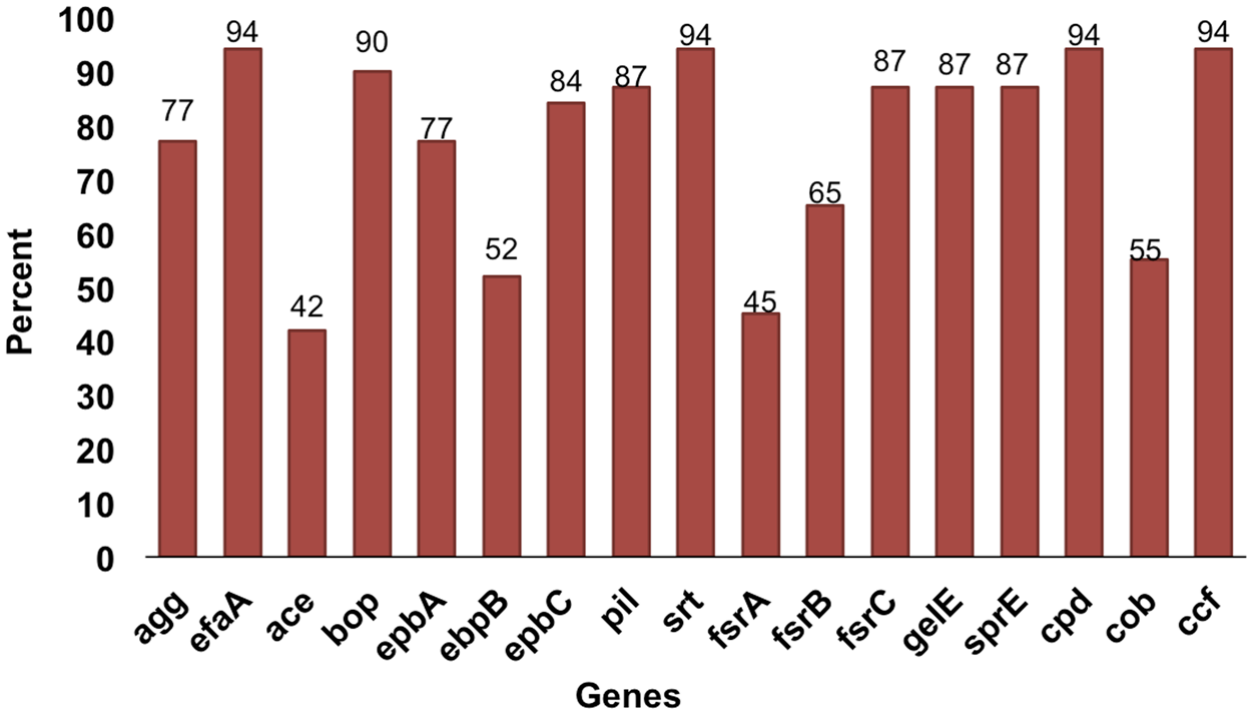
UPGMA analysis to cluster the isolates according to the pattern of their PCR results. A UPGMA cladogram representing the clustering relatedness of 31 isolates according to the pattern of their PCR results for biofilm-associated genes. Isolate names are color coded according to their biofilm strength (Purple, strong; Red, medium; Green, weak; Black, non-biofilm-forming).

Of note, when we tried to use the patterns of gene distribution to classify the isolates, in a similar way to that used with ERIC-PCR analysis, non-biofilm-forming isolates were clustered out, as expected. However, no obvious cluster could sepearte moderate from weak biofilm-formers, but all strong biofilm-forming isolates fell in one major clade (Fig. 4).

### Antibiotic resistance pattern and effect of antibiotic sub-MIC on biofilm formation

The sensitivity of the isolates to azithromycin, ciprofloxacin, vancomycin, gentamycin and tigecycline was estimated by the determination of MIC values by the broth microdilution method. MICs of vancomycin was ≤ 4 µg/ml, tigecycline ≤ 0.25 µg/ml, ciprofloxacin ≤ 1 µg/ml, gentamycin ≤ 500 µg/ml, and azithromycin ≤ 0.5 µg/ml.

**Figure 5:**
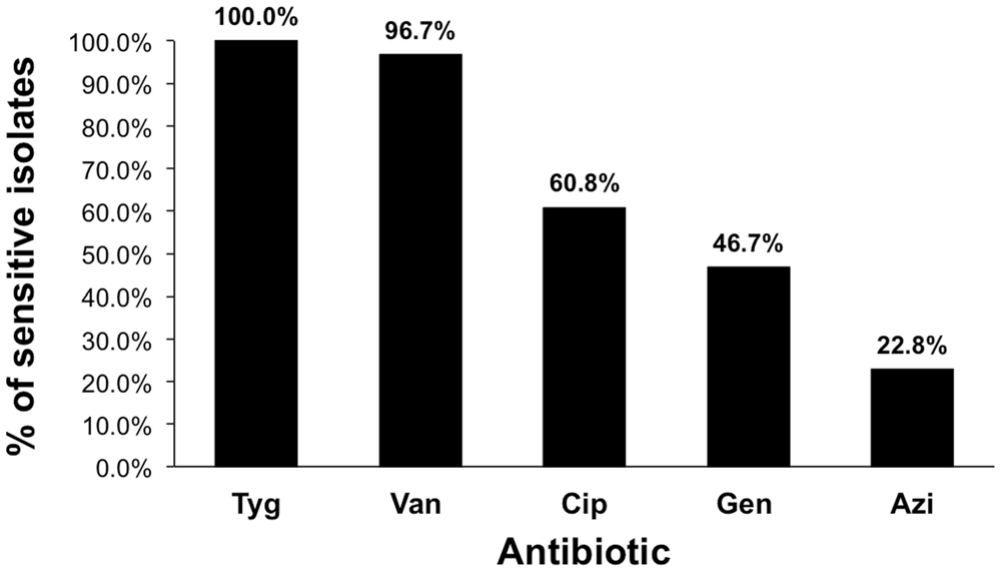
Sensitivity of *Enterococcus* isolates to different antibiotics as analyzed by MIC. Tyg: tigecycline; Van: vancomycin; Cip: ciprofloxacin; Gen: gentamycin; Azi: azithromycin.

**Figure 6:**
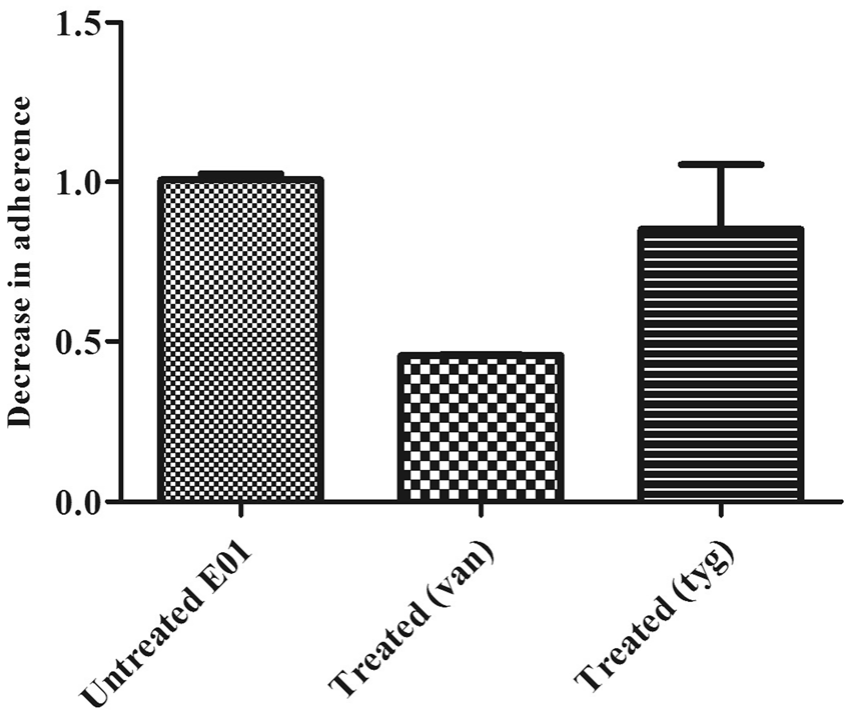
Effect of sub-MIC of some antibiotics on biofilm formation in a representative strong biofilm-forming isolate (E01).

The strains selected for subsequent assays were those with strong biofilm-forming phenotype, which were also gelatinase positive. The five isolates with strong biofilm formation ability were sensitive to vancomycin and tigecycline (Fig. 5). Both antibiotics were tested for their sub-MIC effect on the adherence of the five isolates. In one isolate only, the adherence was significantly decreased by both vancomycin (55% decrease, *p* < 0.05, one-way ANOVA) and tigecycline (42% decrease, *p* < 0.05, one-way ANOVA) in comparison to untreated cells. The adherence of another three isolates was only significantly decreased by vancomycin (50% decrease in adherence, *p* < 0.05, one-way ANOVA). The adherence of one isolate was neither affected by vancomycin nor tigecycline (Fig. 6).

The effect of sub-MIC of vancomycin and tigecycline was also assessed on gelatinase activity in the five strong biofilm-forming isolates. Vancomycin inhibited gelatinase activity in the four isolates with decreased adherence and tigecycline inhibited gelatinase activity in the same isolate in which adherence was reduced.

## Discussion

The main focus of this study was to determine the biofilm-forming ability of clinical enterococcal isolates, its correlation with gelatinase activity and *fsr* locus genotype, as well as its inhibition by antibiotics such as vancomycin. The long-term goal is to define fewer, but more precise, genetic biomarkers to predict different aspects of biofilm formation (i.e., gelatinase activity and biofilm strength) instead of screening a full panel of virulence genes. To this end, a major step was to first establish a good genotype–phenotype correlation relying on genomic analysis to define good biomarker genes for biofilm formation and gelatinase activity, as two separate—even if interdependent—phenotypes. Once the genes were defined, they were validated experimentally.

Our work demonstrates a higher than usual frequency (~96%) of biofilm production by enterococcal clinical isolates collected from Egyptian hospitals, regardless of the strength of the biofilm formed. This frequency is much higher than other reports from developing countries. For instance, Sindhanai *et al*. reported that 68% of isolates in Tamilnadu, India were biofilm producers^21^ and 64.40% of bacterial isolates from urinary tract infection in a hospital in Dhaka, Bangladesh were biofilm producers^22^.

Using already-established Crystal Violet assays, we differentiated the clinical isolates into strong (5.5%), moderate (42%), weak (48%), and non-biofilm-forming (4.5%) strains. Congo-Red agar assay could not differentiate moderate from weak biofilm forming strains, so the results of the Crystal Violet assay were considered. The superiority of Crystal Violet assays has been previously described^21, 23^. Sindhanai and coworkers^21^ reported that 34% of their isolates formed strong biofilms; 49% formed moderate biofilms; and 17% formed weak biofilms. As determined in this study, differentiation between weak and moderate biofilm formation may be associated with the aggregation substance (Agg), as the gene encoding it is enriched in moderate and strong isolates (Table 4). The extent of biofilm formation and the differentiation between weak, medium and strong biofilm-forming capacity is clinically relevant as the potential implication of biofilm formation on hospitalized patients could be serious^24^. Of note, we performed the biofilm assays in TSB medium with additional 0.25% glucose (i.e., a final concentration of 0.5% glucose). There are mixed reports about the effect of glucose on biofilm formation: Kristich *et al*. reported an inhibitory effect of increasing glucose concentrations^25^, yet with a reasonable biofilm formation at 0.5% glucose, whereas Baldassarri *et al*.^26^ and Pillai *et al*.^27^ reported potentiating effect at concentrations <1%. Using 0.5% seemed reasonable. Likewise, we consistently performed the assays after 48 hours incubation based on prior reports^28, 29^; this consistency was important to guarantee that the incubation time would not be a confounding factor.

To better understand the genotype–phenotype correlations for a complex phenotype such as biofilm formation in enterococci, we performed extensive genomic analysis of *E. faecalis*, first starting by screening five well-defined genomes for a set of 17 biofilm-associated genes, and then by extending the screen to ~190 genomes for *agg*, *ace* and *fsr* locus genes.

This genomic analysis was followed by PCR detection of the 17 genes in 31 isolates with variable biofilm formation strength and variable gelatinase activity. Although the entire set of 17 genes was not fully detected in each biofilm-forming strain, many of these genes were present in all the biofilm-forming strains, i.e., *gelE, ccf, sprE, bop, efaA, cpd*, and *fsrC* (Table 3 and Fig. 4). As indicated in Results, in addition to *gelE* (previously determined as a biomarker for biofilm formation), *agg* was found to be a predictor of weak vs. strong/medium biofilm formation (Chi-square *P value* = 0.0026, Table 4).

As for *gelE*, its presence in all biofilm-forming strains and absence in non-biofilm-formers confirmed the reported importance of gelatinase for biofilm formation process^11^. Hancock and Perego showed that GelE is required for the formation of biofilm, as it promotes cell aggregation in microcolonies to develop a three-dimensional structure^11^. Two possibilities were suggested for this function of GelE: either through the enzyme’s proteolytic activity as an initiator of bacterial attachment to surfaces or through the physical presence of the protein rather than its enzymatic activity^11^.

GelE is positively regulated by the quorum sensing-encoding *fsr* locus^30^. Despite the presence of *gelE* gene in 94% of isolates, *in vitro* gelatinase activity was only detected in 30% of the isolates. Other studies, involving large and diverse groups of clinical isolates, showed that 56% and 59% of isolates produced gelatinase, while 88% and 92% were *gelE*-positive, respectively^31, 32^. Among 31 endodontic *Enterococcus* isolates, *gelE* was detected in all tested isolates, whereas the gelatinase activity was found in 74% of them^33^. These reports, together with our results, confirm that *gelE* is obviously essential for gelatinase activity, but that it is not sufficient as *fsrA* and *fsrB* are required for (and highly associated with) the gelatinase phenotype. Because the genomic survey we performed on ~190 genomes (Table S2), as well as our PCR results (Table 3), indicated that *fsrB* is more frequent than *fsrA*, and that no cases were found in which *fsrA* was present in absence of *fsrB*, we suggest that *fsrB* is sufficient to predict the gelatinase activity (Chi-Square *P* value = 0.0012, Table 5).

Thus, we conclude that the absence of gelatinase activity in *gelE-*positive strains is due to the disruption of the *fsr* quorum sensing locus. As described by Nakayama *et al*., the most common cause for loss of gelatinase production is a 23.9 kb deletion encompassing most of the *fsr* locus^34^ (*fsrA* and *fsrB* in particular^35^).

In several pathogens, biofilm production is regulated by quorum sensing systems, including *fsr* in *Enterococcus*, which was shown to have a pronounced effect on biofilms^11^. In the current study the strong biofilm-forming strains contained the entire quorum sensing locus, and loss of any of the quorum sensing genes was correlated with decrease in biofilm strength. Mohamed *et al*.^4^ reported that loss of *fsr* and/or gelatinase function resulted in diminished biofilm formation, in comparison with biofilm production by progenitor, *fsr*-positive, gelatinase-positive *E*. *faecalis* isolate. Carniol and Gilmore discussed the role of signal transduction, quorum sensing and extracellular protease activity in biofilm formation by *E. faecalis* ^36^. Another research team found that biofilm formation was reduced in all three *fsr* mutants (*fsrA*, *fsrB*, *fsrC*) by ~28 to 32% compared to wild-type *E*. *faecalis* OG1RF^4^.

Another focus of this study was the mutual effects between biofilm formation and antibiotics/antibiotic resistance. Here, the antimicrobial susceptibility of *Enterococcus* isolates to azithromycin, ciprofloxacin, vancomycin, gentamycin and tigecycline was estimated through determination of each antibiotic’s MIC by the broth microdilution method. Although studies on the antimicrobial susceptibility patterns of enterococci have affirmed the worldwide emergence of multiple-drug resistant enterococci, particularly to vancomycin^37^, 96.7% of our isolates were actually sensitive to vancomycin. This is not unprecedented, e.g., Karmarkar *et al*. reported that 76% of the isolates they screened were sensitive to vancomycin^38^. Additionally, all the isolates were sensitive to tigecycline, while 60.8% and 46.7% were sensitive to ciprofloxacin and gentamycin, respectively, and only 22.8% were sensitive to azithromycin. Strong biofilm-forming strains were only sensitive to vancomycin and tigecycline.

By evaluating the effect of sub-MIC of vancomycin and tigecycline on adherence of strong biofilm-forming isolates, we found that vancomycin decreased the adherence significantly in four isolates while tigecycline only decreased the adherence in one isolate. Tigecycline has previously been reported to inhibit biofilm formation in enterococci. Maestre and colleagues demonstrated that tigecycline decreased the adherence of 55% of the isolates that had strong biofilm-formation ability^39^. To the best of our knowledge, there are no published reports of biofilm inhibition by vancomycin in enterococci, but there is a precedent case reported for a clinical staphylococcal isolates^16^. Further studies should explore the mechanism of biofilm inhibition by vancomycin and whether it is through direct interaction with the gelatinase enzyme or with any of its regulators. Other possibilities for such inhibition could be related to alteration of gene regulator circuits in response to vancomycin-induced stress or to selection of a bacterial subpopulation that has higher tolerance for low doses of vancomycin.

## Conclusion

We conclude that the biofilm-forming ability of enterococcal isolates sampled in this study, and representing different Egyptian hospitals, does not particularly correlate with their overall genetic make-up, which was highly divergent (as reflected by ERIC-PCR patterns). Several genetic determinants, including the quorum sensing locus, which encodes proteins that regulate biofilm formation, were detected in these biofilm-forming isolates, but none of them was detected in non-biofilm-formers. The genes with highest diagnostic values, as determined by in silico genome analysis and validated by *in vitro* PCR screens and phenotypic assays, are *gelE*, *agg, and fsrB*. Finally, we show that vancomycin, and to a lesser extent tigecycline, could inhibit biofilm formation when used at concentrations below their measured MICs. This work highlights the importance of integrating phenotypic and genotypic assays of biofilm-forming determinants in clinical enterococcal isolates. In addition, it calls for further understanding of the mechanism by which some antibiotics inhibit biofilm formation, thus affecting enterococcal virulence and possibly resistance to other antibiotics.

## Methods

### Bacterial isolates and culture media

Isolates were collected from Egyptian hospitals in the period from 2009 to 2015. *Enterococcus* colonies were isolated by surface streaking of clinical specimens on Enterococcosel agar (Difco laboratories, USA) and identified by Gram stain followed by catalase test. The identity was further confirmed by cultivation on Chromogenic UTI agar (Oxoid, UK), currently known as Brilliance UTI agar. This differential culture medium provides presumptive identification of several urinary tract pathogens. Unlike other species, *Enterococcus* species express beta-glucosidase but not beta-galactosidase or tryptophan deaminase. The beta-glucosidase activity targets the chromogen, x-glucoside, and produces blue colonies. Confirmed *Enterococcus* strains were streaked on Brain Heart agar slants and kept at 4 °C for the period of experimentation. Duplicate glycerol stocks of each isolate were stored at −80 °C.

### Bacterial isolate identification by the VITEK 2 system

A number of isolated colonies (5–10) from a fresh overnight pure culture were resuspended in 3.0 mL of sterile saline solution. The turbidity was adjusted to 0.5 McFarland standard. GP (Gram Positive) identification cards were inoculated with the suspended microorganism by an integrated vacuum apparatus, and test tubes containing the bacterial suspension were placed into a special rack while the identification cards were placed in the neighboring slot. Cards were sealed and inserted into the VITEK 2 reader-incubator, and then subjected to fluorescence measurement every 15 min. Results were obtained automatically and interpreted by the ID-GPC database.

### Polymerase Chain Reaction (PCR) and ERIC-PCR

DNA from prescreened *Enterococcus* strains was extracted as described by Soumet *et al*.^40^. Specific primer pairs were designed and used for amplification of the different genes (Table 2). PCR amplification was performed in a Thermocycler (SensoQuest, Germany) in 0.2 ml reaction tubes, each with 25 µl reaction mixtures. Reaction mixtures were made of 10 pM of each primer, 200 µM of each deoxyribonucleotide (Promega, USA), 5 X reaction buffer, 1.5 mM MgCl_2_, 0.5 U Taq polymerase (Promega, USA) and 0.25 µg extracted enterococcal genomic DNA (or non-template control of nuclease-free water). PCR products were analyzed in 1% agarose gels by electrophoresis, stained with ethidium bromide and visualized under UV light (Fig. S2).

A modified enterobacterial repetitive intergenic consensus PCR (ERIC-PCR) has been developed for typing enterococci^41^. This method was used as previously described, with the primers ERIC-1: ATGTAAGCTCCTGGGGATTCAC and ERIC-2: AAGTAAGTGACTGGG GTGAGCG. PCR conditions were as follows: For ERIC-1, an initial denaturation at 94 °C for 5 min was followed by five cycles of 94 °C for 5 min., 35 °C for 5 min. and 72 °C for 5 min; then 30 cycles of 94 °C for 1 min., 35 °C for 1 min., 72 °C for 2 min and a final extension of 72 °C for 10 min. For ERIC-2, the initial denaturation step was followed by 35 cycles of 94 °C for 1 min., 25 °C for 1 min. then, 72 °C for 4 min, and a final extension of 72 °C for 10 min.

Unweighted pair group method using arithmetic overage algorithm (UPGMA), available at (http://genomes.urv.cat/UPGMA/) was used for ERIC-PCR gel electrophoretic pattern analysis and dendrogram generation. Trees were rendered, colored, and annotated by FigTree v.1.2.3 (obtained from http://tree.bio.ed.ac.uk/software/figtree).

### Congo-red agar biofilm assay

The investigation of biofilm production by the Congo-red agar assay was proposed by Freeman *et al*.^42^. Enterococcal cell suspensions were inoculated on Congo-red agar plates. Black colonies with a dry crystalline consistency indicated a positive result. If the colonies were dark but with no dry crystalline colonial morphology, the biofilm-forming ability was considered *intermediate*. Non-biofilm producers grow as pink colonies (Fig. S3).

### Crystal Violet biofilm assay

Biofilm formation was also assessed by the Crystal Violet assay as described by Christensen *et al*.^43^. Briefly, overnight cultures were inoculated in fresh trypticase soy broth (TSB) containing 0.25% glucose. The culture density was spectrophotometrically adjusted to approximately 0.5 McFarland standard (at absorbance = 600 nm). Each broth culture was diluted 1:100 in fresh broth, and 200 µl of each diluted culture was distributed in three wells of a 96-well microtiter plate and incubated for 48 hours at 37 °C. Negative control wells were included. Planktonic cells were removed, and wells were (i) washed with sterile phosphate-buffered saline for removal of non-adherent cells, (ii) decanted, and then (iii) left to dry. Adherent cells were stained with 200 µl of 2% (W/V) crystal violet for 15 minutes. Excess stain was gently rinsed off with water. Plates were allowed to air-dry. The dye bound to the adherent cells was re-solubilized with 200 µl of 33% (v/v) glacial acetic acid. The optical density (OD) was measured at 545 nm in a plate reader (ELx800, Biotek, USA). Readings from triplicate wells were averaged (Fig. S4).

Strains were classified into categories according to the O.D. measurement of their biofilms as follows: Those with O.D. ≤ O.D.c (O.D. of the negative control) were considered *non-adherent* or biofilm negative. If O.D.c < O.D. ≤ (2x O.D.c), strains were classified as *weakly adherent*; (2x O.D.c) < O.D. ≤ (4x O.D.c) = *moderately adherent*; and (4x O.D.c) < O.D. = *strongly adherent*.

### Gelatinase assay

An inoculum from a pure culture was grown on agar plates containing 3% (w/v) gelatin and incubated for 48 hours. After incubation, plates were flooded with Frazier solution. A positive gelatinase activity was exhibited as transparent halo zones surrounding colonies^44^ (Fig. S5).

### Antibiotics and determination MIC values

The MICs of vancomycin, tigecycline, ciprofloxacin, gentamycin and azithromycin were determined by broth microdilution performed in 96-well plates containing Mueller Hinton broth, in accordance with CLSI guidelines^45^.

### Evaluation of the effect of antibiotic sub-MIC on biofilm formation and gelatinase activity

MICs of different antibiotics were determined by the broth microdilution method, and the antibiotics to which strong biofilm-forming *E. faecalis* isolates were sensitive were selected for studying the effect of their sub-MIC on biofilm production and gelatinase activity. Bacterial suspensions of the strong biofilm-forming isolates were prepared as described under the Crystal Violet assay: Wells (200 µl) were inoculated with bacterial suspensions, TSB containing 0.25% glucose and the corresponding antibiotic sub-MIC concentrations or TSB without antibiotic (antibiotic-free controls). Plates were incubated for 48 hours at 37 °C. The O.D. of isolates with and without antibiotics at sub-MIC was measured at 545 nm using the plate reader as detailed above. Results were taken as the average of triplicate reads.

The evaluation of sub-MIC antibiotic concentrations on gelatinase activity was performed in a similar way to their effect on biofilm formation. Bacterial suspensions were mixed with TSB containing the corresponding antibiotic (at sub-MICs) or supplemented TSB without antibiotic (antibiotic-free controls). A loopful of this mixture was grown on agar plates containing 3% (w/v) gelatin and incubated for 48 hr, then halos were recorded as detailed above.

### Comparative genomic analysis

Five representative strains whose genomes have been previously sequenced were used in this analysis. Three of the five strains have known biofilm formation ability: *E. faecalis* V583 (Accession # AE016830.1), *E. faecalis* OG1RF (Accession # CP002621.1) and *E. faecalis* D32 (Accession # CP003726.1). The other two strains were *E. faecalis* Str. Symbioflor1 (Accession # HF558530.1), a probiotic strain with no biofilm-forming capacity, and *E. faecalis* 62 (Accession # CP002491.1), a commensal strain isolated from a healthy child^46^.

Amino acid and DNA sequences of the selected proteins and their coding genes, respectively, were obtained from NCBI based on the standard strain V583. Analysis tools for genome screens and comparative genomics were BLASTX, BLASTN^47^ (http://blast.ncbi.nlm.nih.gov/) and the SEED subsystems analysis and database comparative genomics tool^20^ (http://pubseed.theseed.org/?page = MultiGenomeCompare http://pubseed.theseed.org/comp_genomes.cgi) with reference organism set to V583 as follows: organism = 226185.1 and comparison organisms set to: 936153.3 (62), 1206105.3 (D32), 474186.5 (OG1RF) and 1261557.3 (Symbioflor 1).

### Statistical Analysis

All statistical analyses, including descriptive statistics and hypothesis tests (e.g., Chi-Square, Fisher Exact Test, ANOVA and Student t-test were performed on Data Desk v. 6.3 (Data Description Inc., Ithaca, NY, USA) and GraphPad Prism (GraphPad Software Tools, Inc., La Jolla, CA, USA).

## Electronic supplementary material

Supplementary material
