# Supplementary material for: Biofilm formation in enterococci: genotype-phenotype correlations and inhibition by vancomycin
Source: Sci Rep. 2017 Jul 18;7:5733. doi: 10.1038/s41598-017-05901-0 (PMC5515943; doi:10.1038/s41598-017-05901-0)
Supplement: Supplementary file 1 — Supplementary material [file 41598_2017_5901_MOESM1_ESM.pdf]

Biofilm formation in enterococci: genotype-phenotype correlations and inhibition by vancomycin

Yomna A. Hashem[1], Heba M. Amin[2], Tamer M. Essam[3], Aymen S. Yassin[3] and Ramy K. Aziz[3]

[1] Department of Microbiology and Immunology, Faculty of Pharmacy, British University in Egypt, Shorouk City, Egypt

[2] Department of Microbiology and Immunology, Faculty of Pharmacy, October University for Modern Sciences and Arts, 6 October City, Egypt

[3] Department of Microbiology and Immunology, Faculty of Pharmacy, Cairo University, Cairo, Egypt

SUPPLEMENTARY INFORMATION

SUPPLEMENTARY FIGURES:

Figure S1: ERIC-PCR cladogram. A full cladogram representation of the UPGMA tree shown in radial format in Fig. 1

Figure S2: Gel-electrophoresis of PCR amplification products of different genes involved in biofilm formation

Figure S3: Congo Red assay for assessment of biofilm formation

Figure S4: Crystal Violet assay for assessment of biofilm formation

Figure S5: Gelatinase test (Gelatinase positive *Enterococcus* on gelatin agar)

SUPPLEMENTARY TABLES:

Table S1: Description of the isolates, their origins, sources, their biofilm phenotypes (Crystal Violet assay optical density and biofilm strength), and their gelatinase phenotype.

Table S2: SEED Subsystems Analysis of 192 enterococcal genomes for the presence of *agg*, *ace*, and the *fsr* locus genes (URL: http://pubseed.theseed.org/?page=SubsystemSelect ; subsystem deposited under the name: "Biofilm Formation in Enterococci").

Table S3: Detailed SEED comparative genomic analysis of four *E. faecalis* genomes against *E. faecalis* V583 (URL: http://pubseed.theseed.org/?page=MultiGenomeCompare http://pubseed.theseed.org/comp_genomes.cgi)

Figure S1

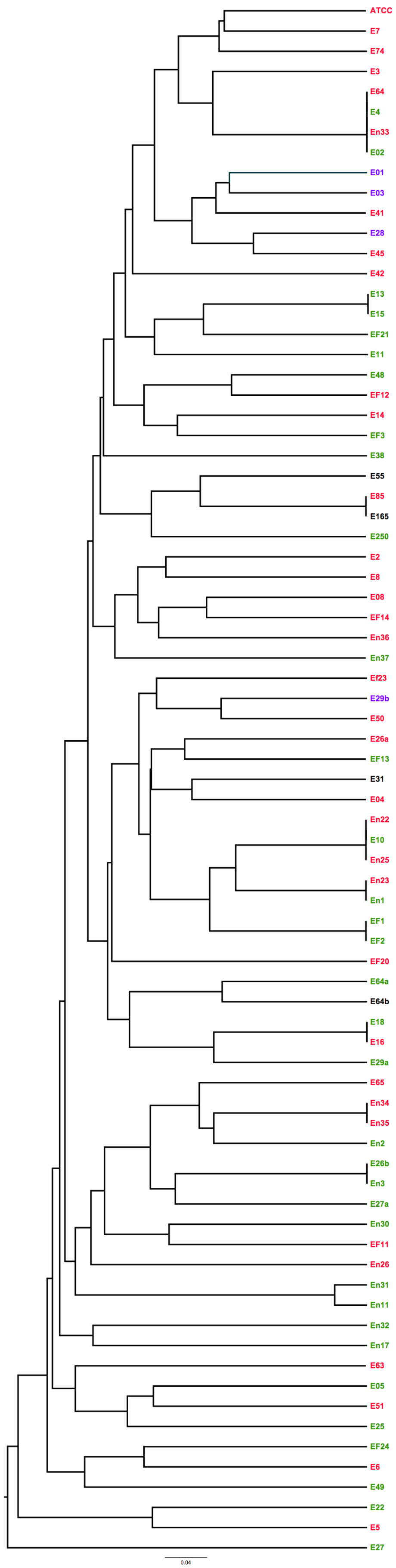

ERIC-PCR Cladogram

Figure S2

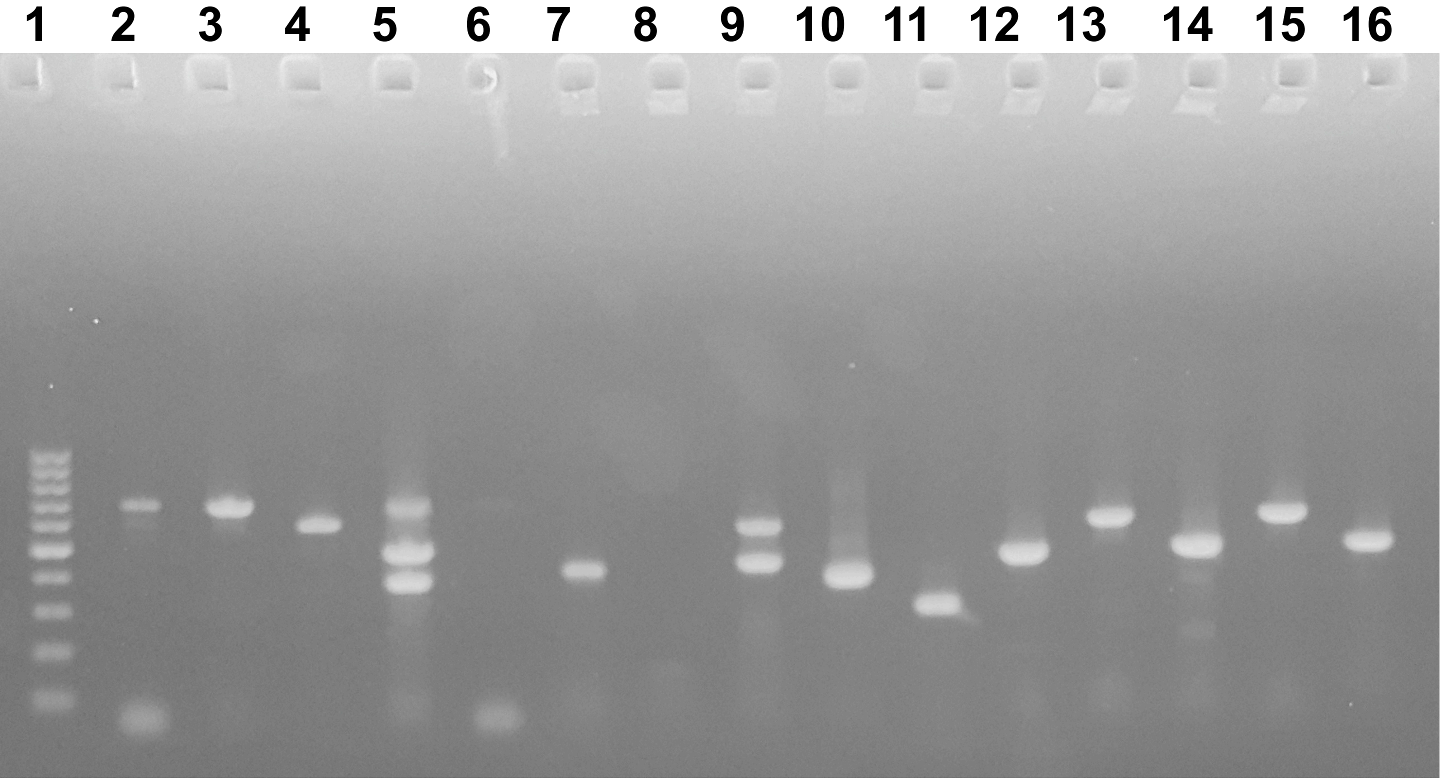

Gel-electrophoresis of PCR amplification products of different genes involved in biofilm formation. Lane 1: 100-bp DNA ladder, 2: *gelE*, lane 3: *efaA*, lane 4: *ace*, lane 5: *fsrC*, *ebpB* and *cob*, lane 7: *sprE*, lane 9: *pilA* and *fsrA*, lane 10: *agg*, lane 11: *bop*, lane 12: *cpd*, lane 13: *srt*, lane 14: *ebpC*, lane 15: *ebpA* and lane16: *fsrB*.

Figure S3

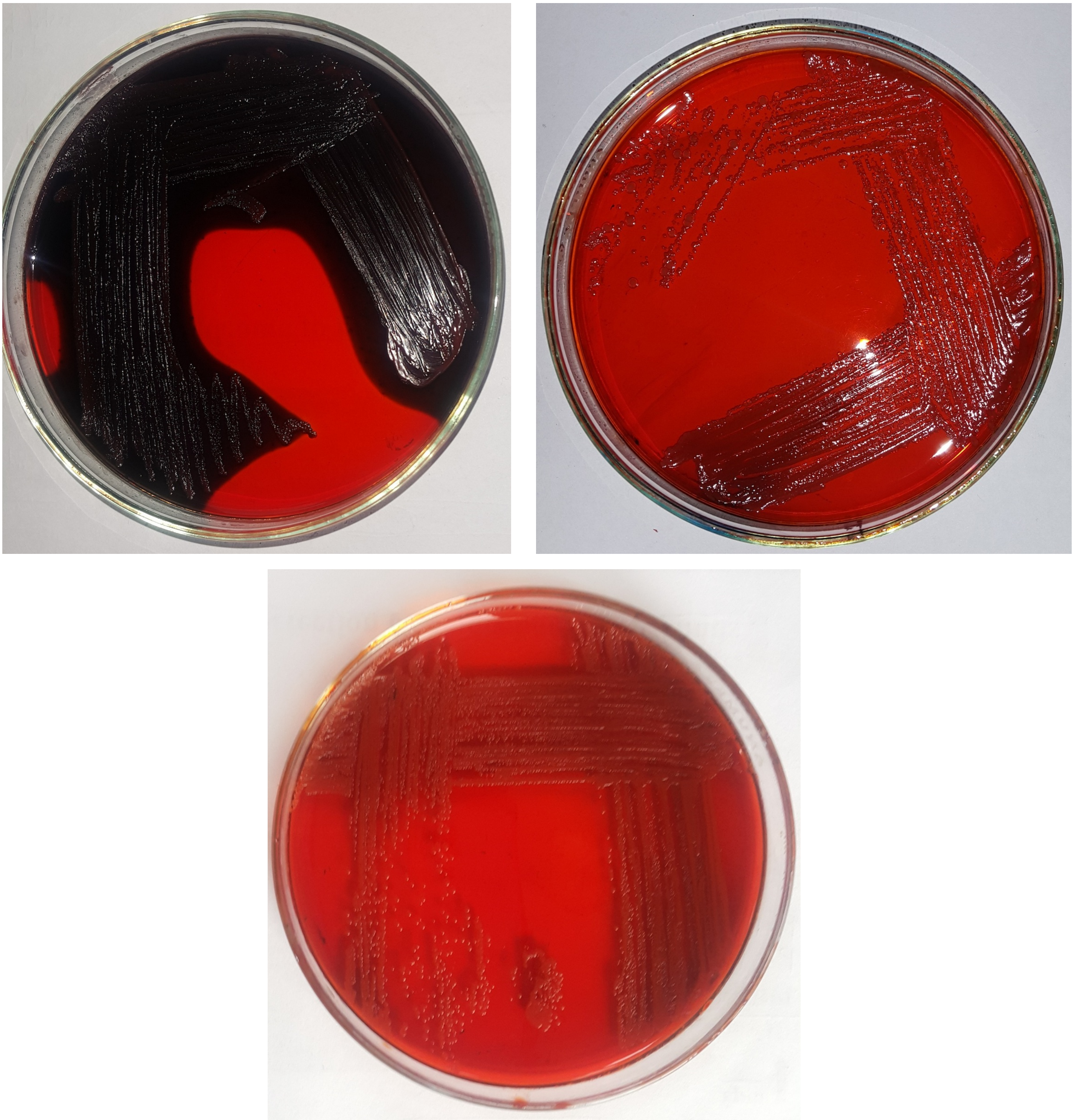

Congo Red assay for assessment of biofilm formation.
left: strong; right: intermediate; bottom: non-forming

Figure S4

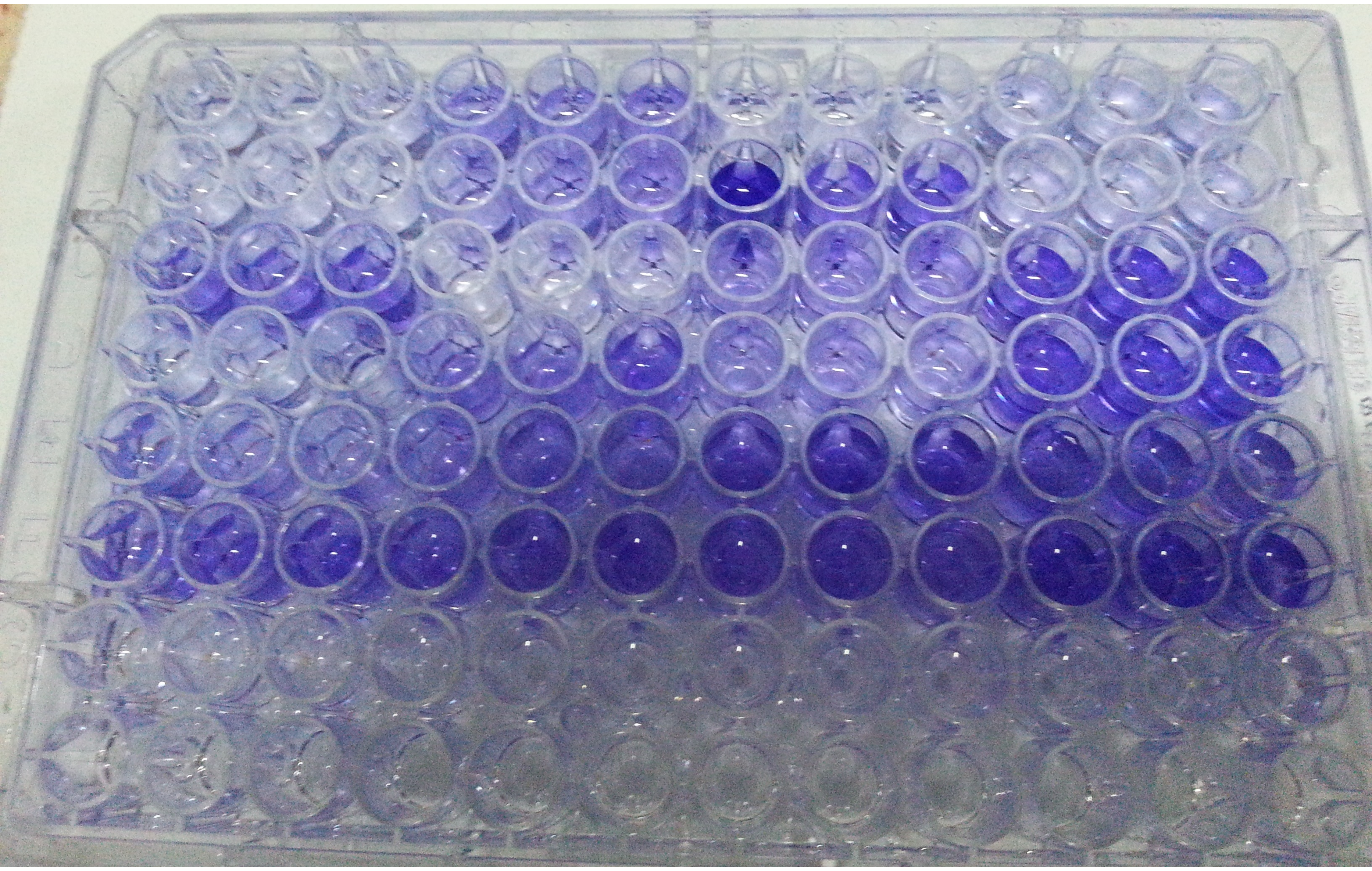

Crystal Violet assay for assessment of biofilm formation

Figure S5

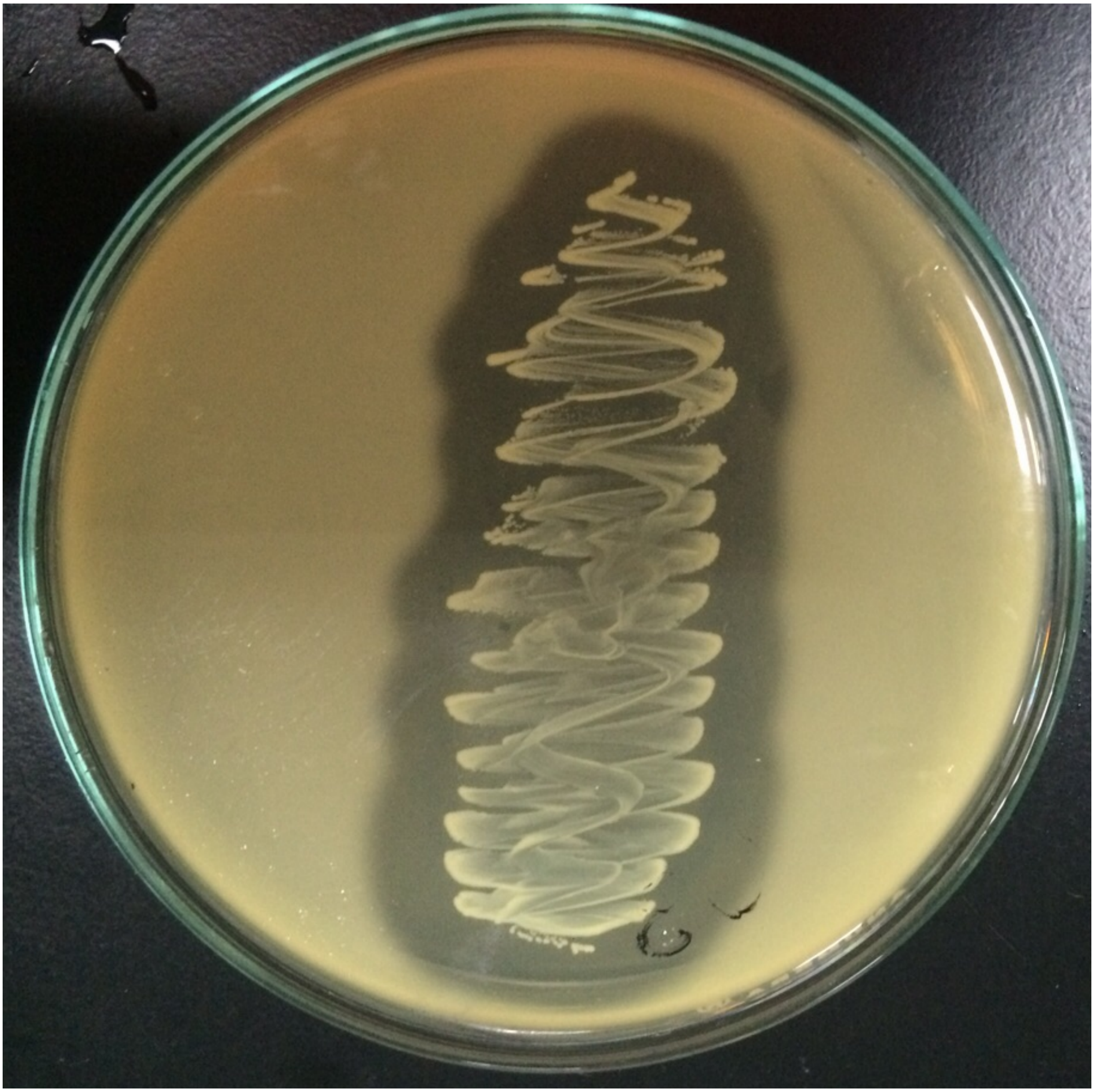

Gelatinase-positive *Enterococcus* on gelatin agar

Table S1: Information about the 90 enterococcal isolates used in this study

Key: S = strong, M = moderate, W = weak, N = non-forming; + = positive, – = negative

Isolate	Source	Hospital/Lab	Species	Biofilm OD	Biofilm Strength	Gelatinase activity
**E03**	urine	AboulRish	*E. faecalis*	1.63	S	+
**E01**	urine	AboulRish	*E. faecalis*	1.62	S	+
**E23a**	vagina	Elkasr El.Eini	*E. faecalis*	1.62	S	–
**E28**	urine	Elkasr El.Eini	*E. faecalis*	1.6	S	+
**E29b**	urine	Elkasr El.Eini	*E. faecalis*	1.6	S	+
**ATCC**	urine	NAMRU	*E. faecalis*	1.1	M	–
**E74**	stool	Elkasr El.Eini	*E. faecalis*	1.1	M	–
**EF11**	urine	AboulRish	*E. faecalis*	1.1	M	–
**E50**	urine	AboulRish	*E. faecium*	1.09	M	–
**E41**	urine	AboulRish	*E. faecalis*	1.07	M	–
**E26a**	urine	Elkasr El.Eini	*E. faecalis*	1.06	M	+
**E44**	urine	AboulRish	*E. faecalis*	1	M	–
**E63**	urine	Elkasr El.Eini	*E. faecalis*	1	M	–
**EF20**	urine	AboulRish	*E. faecalis*	1	M	–
**En35**	urine	El-Monira	*E. faecium*	1	M	–
**En34**	urine	El-Monira	*E. faecium*	0.99	M	–
**E14**	urine	AboulRish	*E. faecalis*	0.98	M	–
**E3**	urine	AboulRish	*E. faecalis*	0.97	M	–
**En14**	urine	El-Monira	*E. faecalis*	0.97	M	–
**En23**	urine	El-Monira	*E. faecium*	0.96	M	–
**E64**	stool	Elkasr El.Eini	*E. faecalis*	0.95	M	–
**En22**	urine	El-Monira	*E. faecalis*	0.95	M	–
**En25**	urine	El-Monira	*E. faecium*	0.94	M	–
**EF12**	urine	AboulRish	*E. faecalis*	0.93	M	–
**E16**	dental	MSA dental clinic	*E. faecalis*	0.92	M	–
**En26**	urine	El-Monira	*E. faecalis*	0.92	M	–
**E5**	stool	Elkasr El.Eini	*E. faecium*	0.91	M	–
**En36**	urine	El-Monira	*E. faecalis*	0.91	M	+
**E8**	stool	Elkasr El.Eini	*E. faecalis*	0.9	M	+
**En33**	urine	El-Monira	*E. faecalis*	0.9	M	+
**E6**	urine	AboulRish	*E. faecalis*	0.89	M	+
**E7**	stool	Elkasr El.Eini	*E. faecalis*	0.89	M	+
**E08**	urine	AboulRish	*E. faecium*	0.86	M	+
**EF14**	urine	AboulRish	*E. faecalis*	0.86	M	–
**EF23**	urine	AboulRish	*E. faecalis*	0.8	M	–
**E51**	urine	AboulRish	*E. faecalis*	0.79	M	–
**E04**	urine	AboulRish	*E. faecalis*	0.77	M	+
**E32**	urine	AboulRish	*E. faecalis*	0.75	M	–
**E85**	dental	MSA dental clinic	*E. faecium*	0.74	M	–
**E2**	urine	AboulRish	*E. faecalis*	0.73	M	–
**E65**	urine	AboulRish	*E. faecalis*	0.71	M	–
**E42**	urine	AboulRish	*E. faecalis*	0.69	M	–
**E45**	urine	Elkasr El.Eini	*E. faecalis*	0.68	M	+
**En27**	urine	El-Monira	*E. faecalis*	0.61	W	–
**E05**	urine	AboulRish	*E. faecalis*	0.6	W	–
**E18**	urine	Elkasr El.Eini	*E. faecalis*	0.6	W	–
**E25**	urine	AboulRish	*E. faecalis*	0.6	W	–
**E26b**	pus	Elkasr El.Eini	*E. faecalis*	0.6	W	+
**E22**	urine	Elkasr El.Eini	*E. faecalis*	0.59	W	–
**E38**	urine	Elkasr El.Eini	*E. faecalis*	0.59	W	–
**EF3**	blood	Elkasr El.Eini	*E. faecium*	0.58	W	–
**E29a**	urine	Elkasr El.Eini	*E. faecalis*	0.54	W	+
**EF2**	blood	AboulRish	*E. faecalis*	0.53	W	–
**En16**	urine	El-Monira	*E. faecalis*	0.52	W	–
**En30**	urine	El-Monira	*E. faecium*	0.51	W	–

**Total Cases**	**90**
**Number of Categories**	**4**

Group	Count	%
E. faecalis	65	72.22
E. faecium	22	24.44
E. casseliflavus	2	2.22
E. gallinarum	1	1.11

**En31**	urine	El-Monira	*E. faecalis*	0.51	W	–
**E20**	urine	AboulRish	*E. casseliflavus*	0.5	W	–
**En17**	urine	El-Monira	*E. faecalis*	0.5	W	–
**EF22**	urine	AboulRish	*E. faecalis*	0.49	W	–
**E48**	urine	Elkasr El.Eini	*E. faecium*	0.48	W	+
**En11**	urine	El-Monira	*E. faecalis*	0.48	W	–
**En32**	urine	El-Monira	*E. faecalis*	0.48	W	–
**E11**	urine	AboulRish	*E. faecalis*	0.47	W	+
**E49**	urine	AboulRish	*E. faecium*	0.47	W	–
**EF24**	urine	AboulRish	*E. faecalis*	0.47	W	+
**En20**	urine	El-Monira	*E. gallinarum*	0.47	W	–
**E02**	urine	AboulRish	*E. faecalis*	0.46	W	+
**E64a**	stool	Elkasr El.Eini	*E. faecalis*	0.46	W	+
**EF21**	urine	AboulRish	*E. faecalis*	0.45	W	+
**En10**	urine	El-Monira	*E. faecalis*	0.45	W	–
**En2**	urine	El-Monira	*E. faecium*	0.45	W	–
**En37**	urine	El-Monira	*E. faecium*	0.45	W	–
**E10**	urine	Elkasr El.Eini	*E. faecalis*	0.44	W	–
**E15**	urine	AboulRish	*E. casseliflavus*	0.44	W	–
**EF1**	blood	AboulRish	*E. faecalis*	0.44	W	–
**En3**	urine	El-Monira	*E. faecalis*	0.44	W	–
**E27**	bile	Elkasr El.Eini	*E. faecalis*	0.43	W	–
**E27a**	bile	Elkasr El.Eini	*E. faecium*	0.43	W	–
**EF25**	urine	AboulRish	*E. faecalis*	0.43	W	–
**E250**	blood	AboulRish	*E. faecalis*	0.42	W	+
**E13**	urine	Elkasr El.Eini	*E. faecalis*	0.41	W	+
**E26**	pus	Elkasr El.Eini	*E. faecium*	0.41	W	+
**En1**	urine	El-Monira	*E. faecium*	0.41	W	–
**En9**	urine	El-Monira	*E. faecium*	0.41	W	–
**E4**	urine	AboulRish	*E. faecalis*	0.4	W	–
**EF13**	blood	AboulRish	*E. faecium*	0.4	W	–
**E165**	dental	MSA dental clinic	*E. faecium*	0.3	N	–
**E31**	stool	Elkasr El.Eini	*E. faecium*	0.3	N	–
**E55**	stool	Elkasr El.Eini	*E. faecium*	0.3	N	–
**E64b**	stool	Elkasr El.Eini	*E. faecalis*	0.3	N	–

Table S2:

#Subsystem: Biofilm Formation in Enterococci
#SEED Subsystems Analysis (URL: http://pubseed.theseed.org/?page=SubsystemSelect)
#Genomes in Fig. 2 are in bold and are boxed
#Numbers in cells reflect gene order in the analyzed genome

Serial #	Organism: Strain name (Genome ID)	Variant Code	Protein-coding genes: *agg*	*ace*	*gelE*	*sprE*	*fsrA*	*fsrB*	*fsrC*	Pattern
1	Enterococcus faecalis B1441 (1157342.3)	1	464	1066	1756	1755	1754	1753	1752	a010010101101101101101
2	Enterococcus faecalis B4638 (1157408.3)	1	465	1055	1746	1745	1744	1743	1742	a010010101101101101101
3	Enterococcus faecalis B4270 (1157405.3)	1	437	1040	1731	1730	1729	1728	1727	a010010101101101101101
4	Enterococcus faecalis B878 (1157366.3)	1	438	1038	1727	1726	1725	1724	1723	a010010101101101101101
5	Enterococcus faecalis B939 (1157367.3)	1	463	1046	1737	1736	1735	1734	1733	a010010101101101101101
6	Enterococcus faecalis B4259 (1157403.3)	1	471	1162	1852	1851	1850	1849	1848	a010010101101101101101
**7**	**Enterococcus faecalis V583 (226185.1)**	1	138, 3154, 3213, 452	1014	1704	1703	1702	1701	1700	a040010101101101101101
8	Enterococcus faecalis V583 (226185.9)	1	136, 3144, 3197, 446	1026	1711	1710	1709	1708	1707	a040010101101101101101
9	Enterococcus faecalis B4163 (1157402.3)	1	798	1470	2160	2159	2158	2157	2156	a010010101101101101101
10	Enterococcus faecalis B4148 (1157401.3)	1	467	1122	1812	1811	1810	1809	1808	a010010101101101101101
11	Enterococcus faecalis UAA1489 (1158976.3)	1	505	1079	1850	1849	1848	1847	1846	a010010101101101101101
12	Enterococcus faecalis AR01/DG (565651.6)	1	2539	2297	382	381	380	379	378	a010010101101101101101
13	Enterococcus faecalis DS16 (1158677.3)	1	2719	921	1558	1557	1556	1555	1554	a010010101101101101101
14	Enterococcus faecalis TX4248 (749495.4)	1	581	869	1444	1445	1446	1447	1448	a010010101101101101101
15	Enterococcus faecalis B4008 (1157399.3)	1	471	1149	1840	1839	1838	1837	1836	a010010101101101101101
16	Enterococcus faecalis HH22 (491075.3)	1	2432, 2437, 2903, 2904, 2905, 2908, 2909	252	663	662	2913	661	660	a522010103103103103103
17	Enterococcus faecalis B2949 (1157394.3)	1	544	1099	1789	1788	1787	1786	1785	a010010101101101101101
18	Enterococcus faecalis B2211 (1157385.3)	1	470	1138	1829	1828	1827	1826	1825	a010010101101101101101
19	Enterococcus faecalis B69486 (1151189.3)	1	2157, 2307	1658	951	952	953	954	955	a020010101101101101101
20	Enterococcus faecalis B1505 (1157343.3)	1	3197, 3219	1048	1739	1738	1737	1736	1735	a020010101101101101101
21	Enterococcus faecalis JH1 (565648.4)	1	2781	635	1576	1575	1574	1573	1572	a010010101101101101101
22	Enterococcus faecalis B2864 (1157392.3)	1	464	1062	1753	1752	1751	1750	1749	a010010101101101101101
23	Enterococcus faecalis TX0645 (749510.3)	1	1521	2432	554	553	552	551	550	a010010101101101101101
24	Enterococcus faecalis B2687 (1157389.3)	1	3168, 3237, 776	1384	2075	2074	2073	2072	2071	a030010101101101101101
25	Enterococcus faecalis TX0635 (749509.3)	1	780	1308	1256	1255	1254	1253	1252	a010010101101101101101
26	Enterococcus faecalis SF19 (1158629.3)	1	426	1090	1783	1782	1781	1780	1779	a010010101101101101101
27	Enterococcus faecalis B2488 (1157357.3)	1	3191, 3237, 464, 468	1054	1745	1744	1743	1742	1741	a040010101101101101101
28	Enterococcus faecalis B1618 (1157346.3)	1	3189, 463	1065	1756	1755	1754	1753	1752	a020010101101101101101
29	Enterococcus faecalis TX0309B (749504.3)	1	1411	2541	284	285	286	287	288	a010010101101101101101
30	Enterococcus faecalis TX0309A (749503.3)	1	664	323	2138	2139	2140	2141	2142	a010010101101101101101
31	Enterococcus faecalis SS-6 (1158621.3)	1	287	1061	1760	1759	1758	1757	1756	a010010101101101101101
32	Enterococcus faecalis TX0104 (491074.3)	1	2320, 2963	478	451	450	2969	449	448	a020010101101101101101
33	Enterococcus faecalis T1 (565636.4)	1	2724	2282	1552	1553	1554	1555	1556	a010010101101101101101
34	Enterococcus faecalis TX0031 (749499.3)	1	166	844	1976	1977	1978	1979	1980	a010010101101101101101
35	Enterococcus faecalis T11 (565640.5)	1	2514	2018	1303	1302	1301	1300	1299	a010010101101101101101
36	Enterococcus faecalis B1385 (1151212.3)	1	2636	2035	1345	1346	1347	1348	1349	a010010101101101101101
37	Enterococcus faecalis B4018 (1157400.3)	1.1	469		1799	1798	1797	1796	1795	a010000101101101101101
38	Enterococcus faecalis B1719 (1157350.3)	1.1	3154, 3173		1771	1770	1769	1768	1767	a020000101101101101101
39	Enterococcus faecalis B1734 (1157351.3)	1.1	464		1757	1756	1755	1754	1753	a010000101101101101101
40	Enterococcus faecalis TR161 (1169314.3)	1.1	501		1775	1774	1773	1772	1771	a010000101101101101101
41	Enterococcus faecalis B1843 (1157352.3)	1.1	467		1756	1755	1754	1753	1752	a010000101101101101101
42	Enterococcus faecalis CH19 (1158627.3)	1.1	285		1706	1705	1704	1703	1702	a010000101101101101101
43	Enterococcus faecalis B1851 (1157381.3)	1.1	3217, 463		1754	1753	1752	1751	1750	a020000101101101101101
44	Enterococcus faecalis B1874 (1157353.3)	1.1	3182, 464		1742	1741	1740	1739	1738	a020000101101101101101
45	Enterococcus faecalis CH188 (565644.5)	1.1	283		1375	1374	1373	1372	1371	a010000101101101101101
46	Enterococcus faecalis B1933 (1157383.3)	1.1	466		1758	1757	1756	1755	1754	a010000101101101101101
47	Enterococcus faecalis B2202 (1157384.3)	1.1	465		1746	1745	1744	1743	1742	a010000101101101101101
48	Enterococcus faecalis T14 (1158626.3)	1.1	2943		1715	1714	1713	1712	1711	a010000101101101101101
49	Enterococcus faecalis CH136 (1158661.3)	1.1	3074		1689	1688	1687	1686	1685	a010000101101101101101
50	Enterococcus faecalis B1696 (1157349.3)	1.1	3206		1739	1738	1737	1736	1735	a010000101101101101101
51	Enterococcus faecalis B1678 (1157348.3)	1.1	463		1746	1745	1744	1743	1742	a010000101101101101101
52	Enterococcus faecalis B1623 (1157347.3)	1.1	3159, 688, 894		2188	2187	2186	2185	2184	a030000101101101101101
53	Enterococcus faecalis SS-7 (1169298.3)	1.1	2852, 405		1668	1667	1666	1665	1664	a020000101101101101101
54	Enterococcus faecalis TX0109 (749502.3)	1.1	840		1344	1345	1346, 2544	2543	2542	a010000101101202102102
55	Enterococcus faecalis B2255 (1157355.3)	1.1	3160, 3213		1746	1745	1744	1743	1742	a020000101101101101101
56	Enterococcus faecalis B2391 (1157356.3)	1.1	3221, 466		1749	1748	1747	1746	1745	a020000101101101101101
57	Enterococcus faecalis SF370 (1158655.3)	1.1	182, 501		1787	1786	1785	1784	1783	a020000101101101101101
58	Enterococcus faecalis B1586 (1157345.3)	1.1	3159		2787	2786	2785	2784	2783	a010000101101101101101
59	Enterococcus faecalis SF339 (1169305.3)	1.1	167		1717	1716	1715	1714	1713	a010000101101101101101
60	Enterococcus faecalis CH116 (1158660.3)	1.1	503		1737	1736	1735	1734	1733	a010000101101101101101
61	Enterococcus faecalis B4672 (1157409.3)	1.1	465		1754	1753	1752	1751	1750	a010000101101101101101
62	Enterococcus faecalis B2535 (1157358.3)	1.1	3201, 3272, 466		1741	1740	1739	1738	1737	a030000101101101101101
63	Enterococcus faecalis SF24397 (1158618.3)	1.1	528		1763	1762	1761	1760	1759	a010000101101101101101
64	Enterococcus faecalis B2557 (1157359.3)	1.1	3159, 3230, 466		1748	1747	1746	1745	1744	a030000101101101101101
65	Enterococcus faecalis B15725 (1151186.3)	1.1	2026		12	11	10	9	8	a010000101101101101101
66	Enterococcus faecalis SF1592 (1158630.3)	1.1	466		1740	1739	1738	1737	1736	a010000101101101101101
67	Enterococcus faecalis SF100 (1158654.3)	1.1	547		1809	1808	1807	1806	1805	a010000101101101101101
68	Enterococcus faecalis S613 (699185.3)	1.1	2805		2180	2179	2178	2177	2176	a010000101101101101101
69	Enterococcus faecalis RM4679 (1158625.3)	1.1	3113		1901	1900	1899	1898	1897	a010000101101101101101
70	Enterococcus faecalis B2593 (1157360.3)	1.1	3223, 806		2095	2094	2093	2092	2091	a020000101101101101101
71	Enterococcus faecalis R712 (699186.3)	1.1	2293		1114	1115	1116	1117	1118	a010000101101101101101
72	Enterococcus faecalis B2685 (1157388.3)	1.1	463		1744	1743	1742	1741	1740	a010000101101101101101
73	Enterococcus faecalis TX0635 = WH245 (749509.5)	1.1	3113		1719	1718	1717	1716	1715	a010000101101101101101
74	Enterococcus faecalis B2802 (1157390.3)	1.1	446		1838	1837	1836	1835	1834	a010000101101101101101
75	Enterococcus faecalis B1532 (1157344.3)	1.1	462		1751	1750	1749	1748	1747	a010000101101101101101
76	Enterococcus faecalis MMH594 (1158653.5)	1.1	464		1754	1753	1752	1751	1750	a010000101101101101101
77	Enterococcus faecalis TX1302 (749513.3)	1.1	2700		2431	2430	2429	2428	2427	a010000101101101101101
78	Enterococcus faecalis B1376 (1151211.3)	1.1	1858		736	735	734	733	732	a010000101101101101101
79	Enterococcus faecalis B1327 (1151210.3)	1.1	206, 3120		1495	1494	1493	1492	1491	a020000101101101101101
80	Enterococcus faecalis B3031 (1157361.3)	1.1	462, 463		1749	1748	1747	1746	1745	a201000102102102102102
81	Enterococcus faecalis B3053 (1157363.3)	1.1	3188, 465		1765	1764	1763	1762	1761	a020000101101101101101
82	Enterococcus faecalis B4969 (1157411.3)	1.1	464		1766	1765	1764	1763	1762	a010000101101101101101
83	Enterococcus faecalis WH257 (1158628.3)	1.1	121, 239		1651	1650	1649	1648	1647	a020000101101101101101
84	Enterococcus faecalis A-3-1 (1169246.3)	1.1	993		1728	1727	1726	1725	1724	a010000101101101101101
85	Enterococcus faecalis B3126 (1157364.3)	1.1	3171, 3189, 466		1783	1782	1781	1780	1779	a030000101101101101101
86	Enterococcus faecalis 7330112-3 (1151199.3)	1.1	2386		1144	1145	1146	1147	1148	a010000101101101101101
87	Enterococcus faecalis B4674 (1157410.3)	1.1	206		1505	1504	1503	1502	1501	a010000101101101101101
88	Enterococcus faecalis B4411 (1157406.3)	1.1	792		2147	2146	2145	2144	2143	a010000101101101101101
89	Enterococcus faecalis B594 (1157365.3)	1.1	459		1755	1754	1753	1752	1751	a010000101101101101101
90	Enterococcus faecalis B4568 (1157407.3)	1.1	465		1776	1775	1774	1773	1772	a010000101101101101101
91	Enterococcus faecalis SF26630 (1158620.3)	1.1	581		1716	1715	1714	1713	1712	a010000101101101101101
92	Enterococcus faecalis CH570 (1158656.3)	1.1	538		1809	1808	1807	1806	1805	a010000101101101101101
93	Enterococcus faecalis V587 (1158623.3)	1.1	210		1503	1502	1501	1500	1499	a010000101101101101101
94	Enterococcus faecalis UAA823 (1157370.3)	1.1	423		1818	1817	1816	1815	1814	a010000101101101101101
95	Enterococcus faecalis B5035 (1158632.3)	1.1	467		1826	1825	1824	1823	1822	a010000101101101101101

**96**	Enterococcus faecalis DAPTO 512 (749489.3)	1.1	1337		2029	2028	2027	2026	2025	a010000101101101101101
**97**	Enterococcus faecalis DAPTO 516 (749490.3)	1.1	2574		1419	1418	1417	1416	1415	a010000101101101101101
**98**	Enterococcus faecalis B1249 (1157380.3)	1.1	771		2060	2059	2058	2057	2056	a010000101101101101101
**99**	Enterococcus faecalis B1290 (1151209.3)	1.1	3036		735	734	733	732	731	a010000101101101101101
**100**	Enterococcus faecalis E1 (1158673.3)	1.1	2891		1690	1689	1688	1687	1686	a010000101101101101101
**101**	Enterococcus faecalis B3336 (1157398.3)	1.1	477		1708	1707	1706	1705	1704	a010000101101101101101
**102**	Enterococcus faecalis B3196 (1157396.3)	1.1	468		1838	1837	1836	1835	1834	a010000101101101101101
**103**	Enterococcus faecalis ERV103 (1134785.3)	1.2	1004	2258	485	486	487	488		a010010101101101101000
**104**	Enterococcus faecalis ERV25 (1134788.3)	1.2	1040	1299	484	485	486	487		a010010101101101101000
**105**	Enterococcus faecalis F1 (1169297.3)	1.4	2938		1795	1794		1790	1789	a010000101101000101101
**106**	Enterococcus faecalis str. 'C 19315 led 1b pp (SCV)' (1151192.3)	1.4	2653, 948, 949		719	718		716	715	a211000102102000102102
**107**	Enterococcus faecalis C19315WT (1151191.3)	1.4	1936, 1937, 441		1705	1704		1702	1701	a211000102102000102102
**108**	Enterococcus faecalis B5076 (1157412.3)	1.4	460		1679	1678		1675	1674	a010000101101000101101
**109**	Enterococcus faecalis SF28073 (1158624.3)	1.4	306		1519	1518		1516	1515	a010000101101000101101
**110**	Enterococcus faecalis T6 (1169291.3)	1.4	264		1665	1664		1662	1661	a010000101101000101101
**111**	Enterococcus faecalis UAA769 (1157369.3)	1.7	2978		1940	1939	1938	1937		a010000101101101101000
**112**	Enterococcus faecalis ERV62 (1134792.3)	1.7	481		1234	1235	1236	852		a010000101101101010000
**113**	Enterococcus faecalis ERV81 (1134798.3)	1.7	438		2586	2587	2588	2589		a010000101101101101000
**114**	Enterococcus faecalis ERV116 (1134786.3)	1.7	2787		2359	2358	2357	2356		a010000101101101101000
**115**	Enterococcus faecalis B1921 (1157382.3)	1.7	404		1636	1635	1634	1633		a010000101101101101000
**116**	Enterococcus faecalis B2207 (1157354.3)	1.7	407		1641	1640	1639	1638		a010000101101101101000
**117**	Enterococcus faecalis ERV31 (1134789.3)	1.7	557		1362	1361	1360	1359		a010000101101101101000
**118**	Enterococcus faecalis ERV85 (1134799.3)	1.7	146		1591	1590	1589	2432		a010000101101101010000
**119**	Enterococcus faecalis ERV129 (1134787.3)	1.7	1416		2950	2949	2948	1413		a010000101101101010000
**120**	Enterococcus faecalis 12107 (1158664.3)	1.8		948	1584	1583	1582	1581		a000010101101101101000
**121**	Enterococcus faecalis ERV65 (1134794.3)	1.8		2582	2832	2833	2834	1562		a000010101101101010000
**122**	Enterococcus faecalis T13 (1158663.3)	1.9		940	1576	1575	1574	1573	1572	a000010101101101101101
**123**	Enterococcus faecalis TX1341 (749514.3)	1.9		924	1903	1904	1905	1906	1907	a000010101101101101101
**124**	Enterococcus faecalis D1 (1169247.3)	1.9		1026	1659	1658	1657	1656	1655	a000010101101101101101
**125**	Enterococcus faecalis TX0630 (749508.3)	1.9		2168	1798	1799	1800	1801	1802	a000010101101101101101
**126**	Enterococcus faecalis T20 (1169285.3)	1.9		886	1512	1511	1510	1509	1508	a000010101101101101101
**127**	Enterococcus faecalis TX0102 (749501.3)	1.9		443	44	43	42	41	40	a000010101101101101101
**128**	Enterococcus faecalis B2277 (1157386.3)	1.9		1145	1838	1837	1836	1835	1834	a000010101101101101101
**129**	Enterococcus faecalis E1Sol (565647.5)	1.9		2269	1592	1593	1594	1595	1596	a000010101101101101101
**130**	Enterococcus faecalis TX0012 (749496.3)	1.9		1411	1871	1872	1873	1874	1875	a000010101101101101101
**131**	Enterococcus faecalis B3119 (1157395.3)	1.9		1140	1830	1829	1828	1827	1826	a000010101101101101101
**132**	Enterococcus faecalis B1005 (1157378.3)	1.9		1193	1883	1882	1881	1880	1879	a000010101101101101101
**133**	Enterococcus faecalis B3042 (1157362.3)	1.9		1051	1743	1742	1741	1740	1739	a000010101101101101101
**134**	Enterococcus faecalis B1138 (1157379.3)	1.9		591	1281	1280	1279	1278	1277	a000010101101101101101
**135**	Enterococcus faecalis T3 (565638.4)	1.9		1491	2179	2178	2177	2176	2175	a000010101101101101101
**136**	Enterococcus faecalis TX1342 (749515.3)	1.9		1124	2534	2533	2532	2531	2530	a000010101101101101101
**137**	Enterococcus faecalis T10 (1169295.3)	1.9		900	1549	1548	1547	1546	1545	a000010101101101101101
**138**	Enterococcus faecalis Merz89 (1169250.3)	1.9		1072	1705	1704	1703	1702	1701	a000010101101101101101
**139**	Enterococcus faecalis TX4244 (749494.3)	1.9		585	1667	1666	1665	1664	1663	a000010101101101101101
**140**	**Enterococcus faecalis OG1RF (474186.5)**	1.9		849	1485	1484	1483	1482	1481	a000010101101101101101
**141**	Enterococcus faecalis TX2137 (749491.3)	1.9		90	321	320	319	318	317	a000010101101101101101
**142**	Enterococcus faecalis TX0470 (749507.3)	1.9		352	2406	2407	2408	2409	2410	a000010101101101101101
**143**	Enterococcus faecalis B2867 (1157393.3)	1.9		1145	1838	1837	1836	1835	1834	a000010101101101101101
**144**	**Enterococcus faecalis D32 (1206105.3)**	1.9		922	1562	1561	1560	1559	1558	a000010101101101101101
**145**	Enterococcus faecalis 599951 (1169281.3)	2	3283	1565			2197	2196	2195	a010010000000101101101
**146**	**Enterococcus faecalis 62 (936153.3)**	2	2942, 3043	1494			2136	2135	2134	a020010000000101101101
**147**	Enterococcus faecalis TX0411 (749506.3)	2	142, 2454, 2455	1285			1901	1902	1903	a211010000000102102102
**148**	Enterococcus faecalis TX0017 (749497.3)	2	692	2361			1938	1939	1940	a010010000000101101101
**149**	Enterococcus faecalis HIP11704 (565646.5)	2	818, 833	2609			2819	2820	2821	a020010000000101101101
**150**	Enterococcus faecalis TX1322 (525278.3)	2	2799	958			284	285	286	a010010000000101101101
**151**	Enterococcus faecalis TX2134 (749518.3)	2	2195, 376	212			1239	1240	1241	a020010000000101101101
**152**	Enterococcus faecalis T7 (1169254.3)	2	2835	1399			2030	2029	2028	a010010000000101101101
**153**	Enterococcus faecalis T2 (565637.4)	2.1	2867				1795	1796	1797	a010000000000101101101
**154**	Enterococcus faecalis SF6375 (1169280.3)	2.1	2676, 3				1537	1536	1535	a020000000000101101101
**155**	Enterococcus faecalis SF5039 (1169252.3)	2.1	3, 543				1876	1875	1874	a020000000000101101101
**156**	Enterococcus faecalis SF350 (1169251.3)	2.1	14, 552				2221	2220	2219	a020000000000101101101
**157**	Enterococcus faecalis Merz151 (1169304.3)	2.1	390, 420				1716	1715	1714	a020000000000101101101
**158**	Enterococcus faecalis E99 (1169284.3)	2.1	2914, 429				1763	1762	1761	a020000000000101101101
**159**	Enterococcus faecalis YI6-1 (1158667.3)	2.1	199, 2347				1696	1695	1694	a020000000000101101101
**160**	Enterococcus faecalis TX0860 (749512.3)	2.1	2924, 2937, 632				132	133	134	a030000000000101101101
**161**	Enterococcus faecalis SF21521 (1158668.3)	2.1	192, 2				1477	1476	1475	a020000000000101101101
**162**	Enterococcus faecalis B56765 (1151188.3)	2.1	1856				951	952	953	a010000000000101101101
**163**	Enterococcus faecalis ATCC 4200 (565642.4)	2.3					2233	2232	2231	a000000000000101101101
**164**	Enterococcus faecalis ATCC 19433 (1169286.3)	2.3					1613	1612	1611	a000000000000101101101
**165**	Enterococcus faecalis T4 (1169253.3)	2.3					1681	1680	1679	a000000000000101101101
**166**	Enterococcus faecalis SF24413 (1158619.3)	3.1	530			1761	1760	1759	1758	a010000000101101101101
**167**	Enterococcus faecalis TR197 (1169307.3)	3.1	2086, 492			1902	1901	1900	1899	a020000000101101101101
**168**	Enterococcus faecalis RC73 (1158678.3)	4	1009	1123	1759	1758		1756	1755	a010010101101000101101
**169**	Enterococcus faecalis Merz96 (565645.4)	4	2671, 617	2341	1728	1729		1730	1731	a020010101101000101101
**170**	Enterococcus faecalis ATCC 10100 (1169312.3)	4.1		1102	1740	1739		1737	1736	a000010101101000101101
**171**	Enterococcus faecalis Fly1 (565649.5)	4.1		2346	1608	1609		1610	1611	a000010101101000101101
**172**	Enterococcus faecalis B653 (1169309.3)	4.8	2624		1476	1475		1473		a010000101101000101000
**173**	Enterococcus faecalis T18 (1169296.3)	4.8	2728		1537	1536		1534		a010000101101000101000
**174**	Enterococcus faecalis X98 (565641.4)	5	2759	2230		1535		1536	1537	a010010000101000101101
**175**	Enterococcus faecalis ATCC 27959 (1158676.3)	5.2			1383		1381	1380	1379	a000000101000101101101
**176**	Enterococcus faecalis HEF39 (1157368.3)	9	163, 164, 438							a211000000000000000000
**177**	Enterococcus faecalis DS5 (565643.5)	9	1659, 2072, 2850							a030000000000000000000
**178**	Enterococcus faecalis UAA1014 (1158546.3)	9	25, 509							a020000000000000000000
**179**	Enterococcus faecalis 1448E03 (1151193.3)	9	161, 440							a020000000000000000000
**180**	Enterococcus faecalis 19116 (1151195.3)	9	1095							a010000000000000000000
**181**	Enterococcus faecalis UAA1180 (1158552.3)	9	281							a010000000000000000000
**182**	**Enterococcus faecalis str. Symbioflor 1 (1261557.3)**	9	397							a010000000000000000000
**183**	Enterococcus faecalis 7430821-4 (1151208.3)	9	2712							a010000000000000000000
**184**	Enterococcus faecalis 7430416-3 (1151207.3)	9	398							a010000000000000000000
**185**	Enterococcus faecalis 7430275-3 (1151205.3)	9	2010							a010000000000000000000
**186**	Enterococcus faecalis 7330948-5 (1151204.3)	9	1550							a010000000000000000000
**187**	Enterococcus faecalis 7330259-5 (1151202.3)	9	2760, 2807							a020000000000000000000
**188**	Enterococcus faecalis 7330257-1 (1151201.3)	9	2003							a010000000000000000000
**189**	Enterococcus faecalis B16457 (1151187.3)	9	1179, 1180, 2475							a211000000000000000000
**190**	Enterococcus faecalis B84847 (1151190.3)	9	1703							a010000000000000000000
**191**	Enterococcus faecalis TUSoD Ef11 (553209.3)	9.1	2151	2313						a010010000000000000000
**192**	Enterococcus faecalis D6 (565650.5)	9.1	149	749						a010010000000000000000

Compared organisms:

**Reference**	*Enterococcus faecalis* V583 (226185.1)
**Comparison Organism 1**	*Enterococcus faecalis* 62 (936153.3)
**Comparison Organism 2**	*Enterococcus faecalis* D32 (1206105.3)
**Comparison Organism 3**	*Enterococcus faecalis* OG1RF (474186.5)
**Comparison Organism 4**	*Enterococcus faecalis* str. Symbioflor 1 (1261557.3)

Percent protein sequence identity

Bidirectional best hit	100	99.9	99.8	99.5	99	98	95	90	80	70	60	50	40	30	20	10
Unidirectional best hit	100	99.9	99.8	99.5	99	98	95	90	80	70	60	50	40	30	20	10

226185.1			936153.3			1206105.3			474186.5			1261557.3		
Contig	Gene	Length	Hit	Contig	Gene	Hit	Contig	Gene	Hit	Contig	Gene	Hit	Contig	Gene
1	1	448	bi	1	384	bi	1	1	bi	1	1	bi	1	1
1	2	377	bi	1	385	bi	1	2	bi	1	2	bi	1	2
1	3	82	bi	1	386	bi	1	3	bi	1	3	bi	1	3
1	4	376	bi	1	387	bi	1	4	bi	1	4	bi	1	4
1	5	643	bi	1	388	bi	1	5	bi	1	5	bi	1	5
1	6	834	bi	1	389	bi	1	6	bi	1	6	bi	1	6
1	7	101	bi	1	390	bi	1	7	bi	1	7	bi	1	7
1	8	189	bi	1	391	bi	1	8	bi	1	8	bi	1	8
1	9	80	bi	1	392	bi	1	9	bi	1	9	bi	1	9
1	10	659	bi	1	393	bi	1	10	bi	1	10	bi	1	10
1	11	151	bi	1	394	bi	1	11	bi	1	11	bi	1	11
1	12	456	bi	1	395	bi	1	15	bi	1	12	bi	1	12
1	13	431	bi	1	396	bi	1	16	bi	1	13	bi	1	13
1	14	290	bi	1	397	bi	1	17	bi	1	14	bi	1	14
1	15	544	bi	1	398	bi	1	18	bi	1	15	bi	1	15
1	16	962	bi	1	399	bi	1	19	bi	1	16	bi	1	16
1	17	171	bi	1	400	bi	1	20	bi	1	17	bi	1	17
1	18	331	bi	1	401	bi	1	21	bi	1	18	bi	1	18
1	19	268	bi	1	402	bi	1	22	bi	1	19	bi	1	19
1	20	304	bi	1	403	bi	1	23	bi	1	20	bi	1	20
1	21	126	bi	1	404	bi	1	24	bi	1	21	bi	1	21
1	22	176	bi	1	405	bi	1	25	bi	1	22	bi	1	22
1	23	68	bi	1	406	bi	1	26	bi	1	23	bi	1	23
1	24	257	bi	1	407	bi	1	27	bi	1	24	bi	1	24
1	25	523	bi	1	408	bi	1	28	bi	1	25	bi	1	25
1	26	405	bi	1	409	bi	1	29	bi	1	26	bi	1	26
1	27	127	bi	1	410	bi	1	30	bi	1	27	bi	1	27
1	28	864	bi	1	411	bi	1	31	bi	1	28	bi	1	32
1	29	872	bi	1	413	bi	1	32	bi	1	29	bi	1	33
1	30	50	bi	1	414	bi	1	33	bi	1	30	bi	1	34
1	31	170	bi	1	415	bi	1	34	bi	1	31	bi	1	35
1	32	156	bi	1	416	bi	1	35	bi	1	32	bi	1	36
1	33	312	bi	1	417	bi	1	36	bi	1	33	bi	1	37
1	34	418	bi	1	418	bi	1	37	bi	1	34	bi	1	38
1	35	272	bi	1	419	bi	1	38	bi	1	35	bi	1	39
1	36	160	bi	1	420	bi	1	39	bi	1	36	bi	1	40
1	37	457	bi	1	421	bi	1	40	bi	1	37	bi	1	41
1	38	382	bi	1	422	bi	1	41	bi	1	38	bi	1	42
1	39	158	bi	1	423	bi	1	42	bi	1	39	bi	1	43
1	40	486	bi	1	424	bi	1	43	bi	1	40	bi	1	44
1	41	180	bi	1	425	bi	1	44	bi	1	41	bi	1	45
1	42	471	bi	1	426	bi	1	45	bi	1	42	bi	1	46

1	43	133	bi	1	427	bi	1	46	bi	1	43	bi	1	47
1	44	283	bi	1	428	bi	1	47	bi	1	44	bi	1	48
1	45	177	bi	1	429	bi	1	48	bi	1	45	bi	1	49
1	46	189	bi	1	430	bi	1	49	bi	1	46	bi	1	50
1	47	82	bi	1	431	bi	1	50	bi	1	47	bi	1	51
1	48	284	bi	1	432	bi	1	51	bi	1	48	bi	1	52
1	49	209	-			-			-			-		
1	50	290	uni	1	2501	uni	1	2017	uni	1	1757	uni	1	1906
1	51	137	-			-			-			-		
1	52	318	bi	1	433	bi	1	52	bi	1	49	bi	1	53
1	53	231	bi	1	434	bi	1	53	bi	1	50	bi	1	54
1	54	280	bi	1	435	bi	1	54	bi	1	51	bi	1	55
1	55	274	bi	1	436	bi	1	55	bi	1	52	bi	1	56
1	56	459	bi	1	437	bi	1	56	bi	1	53	bi	1	57
1	57	1314	bi	1	439	bi	1	57	bi	1	54	bi	1	58
1	58	560	bi	1	440	bi	1	60	bi	1	55	bi	1	59
1	59	75	bi	1	441	bi	1	61	bi	1	56	bi	1	60
1	60	329	bi	1	442	bi	1	62	bi	1	57	bi	1	61
1	61	205	bi	1	443	bi	1	64	bi	1	58	bi	1	63
1	62	339	bi	1	444	bi	1	65	bi	1	59	bi	1	64
1	63	146	-			bi	1	66	bi	1	60	bi	1	65
1	64	233	bi	1	445	bi	1	67	bi	1	61	bi	1	66
1	65	780	bi	1	446	bi	1	68	bi	1	62	bi	1	67
1	66	306	bi	1	447	bi	1	69	bi	1	63	bi	1	68
1	67	218	bi	1	448	bi	1	70	bi	1	64	bi	1	70
1	68	298	bi	1	450	bi	1	72	bi	1	65	bi	1	72
1	69	191	bi	1	451	bi	1	73	bi	1	66	bi	1	73
1	70	64	bi	1	452	bi	1	74	bi	1	67	bi	1	74
1	71	171	bi	1	453	bi	1	75	bi	1	68	bi	1	75
1	72	187	bi	1	454	bi	1	76	bi	1	69	bi	1	76
1	73	81	bi	1	455	bi	1	77	bi	1	70	bi	1	77
1	74	412	bi	1	461	bi	1	78	bi	1	76	bi	1	78
1	75	60	bi	1	462	bi	1	79	bi	1	77	bi	1	79
1	76	128	uni	1	463	uni	1	80	uni	1	79	uni	1	80
1	77	368	bi	1	463	bi	1	81	bi	1	79	bi	1	80
1	78	496	bi	1	464	bi	1	82	bi	1	80	bi	1	81
1	79	1988	bi	1	466	bi	1	84	bi	1	81	bi	1	82
1	80	305	bi	1	467	bi	1	85	bi	1	82	bi	1	83
1	81	502	bi	1	468	bi	1	86	bi	1	83	bi	1	84
1	82	80	bi	1	469	bi	1	87	bi	1	84	bi	1	85
1	83	1056	bi	1	470	bi	1	88	bi	1	85	bi	1	86
1	84	257	bi	1	471	bi	1	89	bi	1	86	bi	1	87
1	85	262	bi	1	472	bi	1	90	bi	1	87	bi	1	88
1	86	227	bi	1	473	bi	1	91	bi	1	88	bi	1	90
1	87	369	bi	1	474	bi	1	92	bi	1	89	bi	1	92
1	88	220	bi	1	475	bi	1	93	bi	1	90	bi	1	93
1	89	298	bi	1	476	bi	1	94	bi	1	91	bi	1	94
1	90	424	bi	1	477	bi	1	95	bi	1	92	bi	1	95
1	91	305	bi	1	478	bi	1	96	bi	1	93	bi	1	96
1	92	160	bi	1	479	bi	1	97	bi	1	94	bi	1	97
1	93	163	bi	1	480	bi	1	98	bi	1	95	bi	1	98
1	94	409	bi	1	481	bi	1	99	bi	1	96	bi	1	99
1	95	340	bi	1	482	bi	1	100	bi	1	97	bi	1	100
1	96	311	bi	1	483	bi	1	101	bi	1	98	bi	1	101
1	97	231	bi	1	484	bi	1	102	bi	1	99	bi	1	102
1	98	484	bi	1	485	bi	1	103	bi	1	100	bi	1	103
1	99	227	bi	1	486	bi	1	104	bi	1	101	bi	1	104

1	100	112	bi	1	487	bi	1	105	bi	1	102	bi	1	105
1	101	340	bi	1	488	bi	1	106	bi	1	103	bi	1	106
1	102	268	-			-			-			-		
1	103	272	-			-			-			-		
1	104	836	bi	1	489	bi	1	107	bi	1	104	bi	1	107
1	105	423	bi	1	490	bi	1	108	bi	1	106	bi	1	108
1	106	148	bi	1	491	bi	1	109	bi	1	107	bi	1	109
1	107	471	bi	1	492	bi	1	110	bi	1	108	bi	1	110
1	108	322	bi	1	493	bi	1	111	bi	1	109	bi	1	111
1	109	263	bi	1	494	bi	1	112	bi	1	110	bi	1	112
1	110	179	bi	1	495	bi	1	113	bi	1	111	bi	1	113
1	111	76	-			-			-			-		
1	112	126	-			bi	1	114	-			-		
1	113	784	-			bi	1	115	bi	1	2183	-		
1	114	120	bi	1	496	bi	1	116	-			bi	1	114
1	115	391	uni	1	2146	-			-			bi	1	1007
1	116	206	-			bi	1	117	-			-		
1	117	192	-			-			-			-		
1	118	213	uni	1	2344	uni	1	1707	-			-		
1	119	125	uni	1	1316	uni	1	1197	uni	1	1122	uni	1	1193
1	120	61	-			-			-			-		
1	121	84	-			-			-			-		
1	122	109	-			-			-			-		
1	123	136	-			-			-			-		
1	124	75	-			-			-			-		
1	125	81	-			-			-			-		
1	126	135	-			-			-			-		
1	127	111	-			-			-			-		
1	128	128	-			-			-			-		
1	129	504	uni	1	2202	uni	1	1720	uni	1	769	uni	1	1718
1	130	178	-			-			-			-		
1	131	96	-			-			-			-		
1	132	135	-			-			-			-		
1	133	448	uni	1	2481	uni	1	1999	uni	1	772	-		
1	134	205	-			-			-			-		
1	135	102	uni	3	2938	bi	1	1852	-			-		
1	136	902	uni	4	3045	-			-			-		
1	137	47	-			-			-			-		
1	138	1306	uni	3	2942	-			-			uni	1	397
1	139	286	-			-			-			-		
1	140	185	-			-			-			-		
1	141	148	-			-			-			-		
1	142	283	uni	4	3038	-			-			uni	1	395
1	143	330	uni	4	3033	-			-			-		
1	144	75	-			-			-			-		
1	145	130	-			-			-			-		
1	146	73	bi	2	2927	-			-			-		
1	147	836	uni	4	3028	-			uni	1	780	-		
1	148	655	-			-			-			-		
1	149	346	uni	1	2305	uni	3	2989	-			uni	1	386
1	150	97	-			-			-			bi	1	379
1	151	75	-			-			-			-		
1	152	173	-			-			-			-		
1	153	125	-			-			-			-		
1	154	71	bi	1	529	-			-			-		
1	155	389	bi	1	530	uni	1	1841	uni	1	1321	uni	1	2361
1	156	521	bi	1	531	bi	1	118	bi	1	112	bi	1	115

1	157	308	bi	1	532	bi	1	119	bi	1	113	bi	1	116
1	158	196	bi	1	533	bi	1	120	bi	1	114	bi	1	117
1	159	203	bi	1	534	bi	1	121	bi	1	115	bi	1	118
1	160	338	bi	1	535	bi	1	122	bi	1	116	bi	1	119
1	161	336	bi	1	536	bi	1	123	bi	1	117	bi	1	120
1	162	434	bi	1	537	bi	1	125	bi	1	118	bi	1	121
1	163	221	bi	1	538	bi	1	126	bi	1	119	bi	1	122
1	164	132	bi	1	539	bi	1	127	bi	1	120	bi	1	123
1	165	358	bi	1	540	bi	1	128	bi	1	121	bi	1	124
1	166	362	bi	1	541	bi	1	129	bi	1	122	bi	1	125
1	167	523	bi	1	542	bi	1	130	bi	1	123	bi	1	126
1	168	381	bi	1	543	bi	1	131	bi	1	124	bi	1	127
1	169	318	bi	1	544	bi	1	132	bi	1	125	bi	1	128
1	170	97	bi	4	3002	bi	1	133	uni	1	1104	bi	1	129
1	171	104	uni	4	3002	uni	1	133	-			uni	1	129
1	172	389	bi	1	551	bi	1	134	bi	1	131	bi	1	130
1	173	273	bi	1	552	bi	1	135	bi	1	132	bi	1	131
1	174	236	bi	1	553	bi	1	136	bi	1	133	bi	1	132
1	175	313	bi	1	554	bi	1	137	bi	1	134	bi	1	133
1	176	267	bi	1	555	bi	1	138	bi	1	135	bi	1	134
1	177	337	bi	1	556	bi	1	139	bi	1	136	bi	1	135
1	178	335	bi	1	557	bi	1	140	bi	1	137	bi	1	136
1	179	396	bi	1	558	bi	1	141	bi	1	138	bi	1	137
1	180	229	bi	1	559	bi	1	142	bi	1	139	bi	1	138
1	181	192	bi	1	560	bi	1	143	bi	1	140	bi	1	139
1	182	227	bi	1	561	bi	1	144	bi	1	141	bi	1	140
1	183	138	bi	1	562	bi	1	145	bi	1	142	bi	1	141
1	184	157	bi	1	563	bi	1	146	bi	1	143	bi	1	142
1	185	694	bi	1	564	bi	1	147	bi	1	144	bi	1	143
1	186	396	bi	1	565	bi	1	148	bi	1	145	bi	1	144
1	187	275	bi	1	566	bi	1	149	bi	1	146	bi	1	145
1	188	389	bi	1	567	bi	1	150	bi	1	147	bi	1	146
1	189	103	bi	1	568	bi	1	151	bi	1	148	bi	1	148
1	190	210	bi	1	569	bi	1	152	bi	1	149	bi	1	149
1	191	208	bi	1	570	bi	1	153	bi	1	150	bi	1	150
1	192	97	bi	1	571	bi	1	154	bi	1	151	bi	1	151
1	193	277	bi	1	572	bi	1	155	bi	1	152	bi	1	152
1	194	93	bi	1	573	bi	1	156	bi	1	153	bi	1	153
1	195	116	bi	1	574	bi	1	157	bi	1	154	bi	1	154
1	196	219	bi	1	575	bi	1	158	bi	1	155	bi	1	155
1	197	145	bi	1	576	bi	1	159	bi	1	156	bi	1	156
1	198	63	bi	1	577	bi	1	160	bi	1	157	bi	1	157
1	199	89	bi	1	578	bi	1	161	bi	1	158	bi	1	158
1	200	123	bi	1	579	bi	1	162	bi	1	159	bi	1	159
1	201	104	bi	1	580	bi	1	163	bi	1	160	bi	1	160
1	202	180	bi	1	581	bi	1	164	bi	1	161	bi	1	161
1	203	62	bi	1	582	bi	1	165	bi	1	162	bi	1	162
1	204	133	bi	1	583	bi	1	166	bi	1	163	bi	1	163
1	205	179	bi	1	584	bi	1	167	bi	1	164	bi	1	164
1	206	119	bi	1	585	bi	1	168	bi	1	165	bi	1	165
1	207	167	bi	1	586	bi	1	169	bi	1	166	bi	1	166
1	208	60	bi	1	587	bi	1	170	bi	1	167	bi	1	167
1	209	147	bi	1	588	bi	1	171	bi	1	168	bi	1	168
1	210	433	bi	1	589	bi	1	172	bi	1	169	bi	1	169
1	211	217	bi	1	590	bi	1	173	bi	1	170	bi	1	170
1	212	73	bi	1	591	bi	1	174	bi	1	171	bi	1	171
1	213	39	bi	1	592	bi	1	175	bi	1	172	bi	1	172

1	214	122	bi	1	593	bi	1	176	bi	1	173	bi	1	173
1	215	130	bi	1	594	bi	1	177	bi	1	174	bi	1	174
1	216	313	bi	1	595	bi	1	178	bi	1	175	bi	1	175
1	217	128	bi	1	596	bi	1	179	bi	1	176	bi	1	176
1	218	400	bi	1	597	bi	1	180	bi	1	177	bi	1	177
1	219	378	bi	1	598	bi	1	181	bi	1	178	bi	1	178
1	220	280	bi	1	599	bi	1	182	bi	1	179	bi	1	179
1	221	292	bi	1	600	bi	1	183	bi	1	180	bi	1	180
1	222	266	bi	1	601	bi	1	184	bi	1	181	bi	1	181
1	3266	250	bi	1	602	bi	1	185	bi	1	182	bi	1	182
1	223	104	bi	1	603	bi	1	186	bi	1	183	bi	1	183
1	224	452	bi	1	604	bi	1	187	bi	1	184	bi	1	184
1	225	164	bi	1	605	bi	1	188	bi	1	185	bi	1	185
1	226	192	bi	1	606	bi	1	189	bi	1	186	bi	1	186
1	227	247	bi	1	607	bi	1	190	bi	1	187	bi	1	187
1	228	528	bi	1	608	bi	1	191	bi	1	188	bi	1	188
1	229	99	bi	1	609	bi	1	192	bi	1	189	bi	1	189
1	230	238	bi	1	610	bi	1	193	bi	1	190	bi	1	190
1	231	282	bi	1	611	bi	1	194	bi	1	191	bi	1	191
1	232	171	bi	1	612	bi	1	195	bi	1	192	bi	1	192
1	233	504	bi	1	613	bi	1	196	bi	1	193	bi	1	193
1	234	497	bi	1	614	bi	1	197	bi	1	194	bi	1	194
1	235	328	bi	1	615	bi	1	198	bi	1	195	bi	1	195
1	236	189	bi	1	616	bi	1	199	bi	1	196	bi	1	196
1	237	1190	bi	1	617	bi	1	200	bi	1	197	bi	1	197
1	238	530	bi	1	618	bi	1	201	bi	1	198	bi	1	198
1	239	88	bi	1	619	bi	1	202	bi	1	199	bi	1	199
1	240	56	bi	1	620	bi	1	203	bi	1	200	bi	1	200
1	241	160	bi	1	621	bi	1	204	bi	1	201	bi	1	201
1	242	160	bi	1	622	bi	1	205	bi	1	202	bi	1	202
1	243	463	bi	1	623	bi	1	206	bi	1	203	bi	1	203
1	244	182	bi	1	624	bi	1	207	bi	1	204	bi	1	204
1	245	719	bi	1	625	bi	1	208	bi	1	205	bi	1	205
1	246	299	bi	1	626	bi	1	209	bi	1	206	bi	1	206
1	247	333	bi	1	627	bi	1	210	bi	1	207	bi	1	207
1	248	499	bi	1	628	bi	1	211	bi	1	208	bi	1	208
1	3267	208	bi	1	630	bi	1	213	bi	1	209	uni	1	2075
1	3268	38	uni	1	630	-			uni	1	209	-		
1	249	633	bi	1	631	uni	1	213	bi	1	210	bi	1	209
1	250	477	bi	1	632	bi	1	214	bi	1	211	bi	1	210
1	251	489	bi	1	633	bi	1	215	bi	1	212	bi	1	211
1	252	273	bi	1	634	bi	1	216	bi	1	213	bi	1	212
1	253	248	bi	1	635	bi	1	217	bi	1	214	bi	1	215
1	254	189	bi	1	636	bi	1	218	bi	1	215	bi	1	216
1	255	144	bi	1	637	bi	1	219	bi	1	216	bi	1	217
1	256	171	bi	1	638	bi	1	220	bi	1	217	bi	1	218
1	257	225	bi	1	639	bi	1	221	bi	1	218	bi	1	219
1	258	312	bi	1	640	bi	1	222	bi	1	219	bi	1	220
1	259	133	bi	1	641	bi	1	223	bi	1	220	bi	1	221
1	260	251	bi	1	642	bi	1	224	bi	1	221	bi	1	222
1	261	413	bi	1	643	bi	1	225	bi	1	222	bi	1	223
1	262	145	bi	1	644	bi	1	226	bi	1	223	bi	1	224
1	263	312	bi	1	646	bi	1	227	bi	1	224	bi	1	226
1	264	216	bi	1	647	bi	1	228	bi	1	226	bi	1	227
1	265	187	bi	1	648	bi	1	229	bi	1	227	bi	1	228
1	266	46	bi	1	649	bi	1	230	bi	1	228	bi	1	229
1	267	306	bi	1	650	bi	1	231	bi	1	229	bi	1	230

1	268	379	bi	1	651	bi	1	232	bi	1	230	bi	1	231
1	269	477	bi	1	652	bi	1	233	bi	1	231	bi	1	232
1	270	445	bi	1	653	bi	1	234	bi	1	232	bi	1	233
1	271	254	bi	1	654	bi	1	235	bi	1	233	bi	1	234
1	272	181	bi	1	655	bi	1	236	bi	1	234	bi	1	235
1	273	456	bi	1	656	bi	1	237	bi	1	235	bi	1	236
1	274	388	bi	1	657	bi	1	238	bi	1	236	bi	1	237
1	275	147	bi	1	658	bi	1	239	bi	1	237	bi	1	238
1	276	829	bi	1	659	bi	1	240	bi	1	238	bi	1	239
1	277	69	bi	1	660	bi	1	241	bi	1	239	bi	1	240
1	278	457	bi	1	661	bi	1	242	bi	1	240	bi	1	241
1	279	130	bi	1	662	bi	1	243	bi	1	241	bi	1	242
1	280	462	bi	1	663	bi	1	244	bi	1	242	bi	1	243
1	281	405	uni	1	1861	bi	1	245	uni	1	1219	uni	1	1546
1	282	177	-			bi	1	246	-			-		
1	283	226	uni	1	2344	bi	1	247	-			-		
1	284	121	uni	1	1316	bi	1	248	-			-		
1	285	71	-			bi	1	249	-			-		
1	286	112	-			bi	1	250	-			-		
1	287	91	-			bi	1	251	-			bi	1	1856
1	288	47	-			bi	1	252	-			bi	1	1536
1	289	67	-			bi	1	253	-			-		
1	290	73	-			-			-			-		
1	291	60	-			bi	1	2480	-			bi	1	1855
1	292	65	-			bi	1	255	-			-		
1	293	230	-			bi	1	256	-			-		
1	294	84	-			-			-			-		
1	295	64	-			bi	1	257	-			-		
1	296	160	-			bi	1	258	-			-		
1	297	391	-			bi	1	259	-			-		
1	298	83	-			bi	1	260	-			-		
1	299	110	-			bi	1	261	-			-		
1	300	192	-			bi	1	262	-			-		
1	301	74	-			bi	1	263	-			-		
1	302	183	-			bi	1	264	-			-		
1	303	654	-			bi	1	265	-			-		
1	304	80	-			-			-			-		
1	305	101	-			-			-			-		
1	306	813	-			bi	1	267	-			-		
1	307	107	-			bi	1	268	-			-		
1	308	458	uni	1	2785	bi	1	269	uni	1	2004	uni	1	2158
1	309	147	-			bi	1	270	-			-		
1	310	150	-			bi	1	271	-			-		
1	311	419	-			bi	1	272	-			-		
1	312	511	-			bi	1	275	-			-		
1	313	488	uni	1	2319	bi	1	276	-			-		
1	314	68	-			-			-			-		
1	315	70	-			bi	1	278	-			-		
1	316	191	-			bi	1	279	-			-		
1	317	288	-			bi	1	280	-			bi	1	1507
1	318	51	-			bi	1	281	-			-		
1	319	181	-			bi	1	282	-			-		
1	320	113	-			bi	1	283	-			-		
1	321	144	-			bi	1	284	-			-		
1	322	128	-			bi	1	285	-			-		
1	323	209	-			bi	1	286	-			-		
1	324	163	-			-			-			-		

1	3269	147	-			bi	1	287	-			-		
1	325	96	-			bi	1	288	-			-		
1	326	1050	uni	1	2307	bi	1	289	uni	1	1025	uni	1	1498
1	327	292	-			bi	1	290	-			-		
1	328	362	-			bi	1	291	-			-		
1	329	345	bi	1	1362	bi	1	292	-			-		
1	330	264	-			bi	1	293	-			-		
1	331	85	-			bi	1	294	-			uni	1	2337
1	332	69	-			uni	1	2435	-			uni	1	2336
1	333	434	uni	1	2296	bi	1	2434	uni	1	518	bi	1	2335
1	334	42	-			-			-			-		
1	335	207	bi	1	664	bi	1	297	bi	1	243	bi	1	244
1	336	325	bi	1	665	bi	1	298	bi	1	244	bi	1	245
1	337	112	bi	1	666	bi	1	299	bi	1	245	bi	1	246
1	338	104	bi	1	667	bi	1	300	bi	1	246	bi	1	247
1	339	349	bi	1	668	bi	1	301	bi	1	247	bi	1	248
1	340	195	bi	1	669	bi	1	302	bi	1	248	bi	1	249
1	341	393	uni	1	1025	-			-			uni	1	424
1	342	245	bi	1	670	bi	1	303	bi	1	249	bi	1	250
1	343	278	bi	1	671	bi	1	304	bi	1	250	bi	1	251
1	344	179	bi	1	672	bi	1	305	bi	1	251	bi	1	252
1	345	451	bi	1	673	bi	1	306	bi	1	252	bi	1	253
1	346	229	bi	1	674	bi	1	307	bi	1	253	bi	1	254
1	347	579	bi	1	675	bi	1	308	bi	1	254	bi	1	255
1	348	382	bi	1	676	bi	1	309	bi	1	255	bi	1	256
1	349	231	bi	1	677	bi	1	310	bi	1	256	bi	1	257
1	350	494	bi	1	678	bi	1	311	bi	1	257	bi	1	258
1	351	326	bi	1	679	bi	1	312	bi	1	258	bi	1	259
1	352	322	bi	1	680	bi	1	313	bi	1	259	bi	1	260
1	353	348	bi	1	681	bi	1	314	bi	1	260	bi	1	261
1	354	202	bi	1	683	bi	1	316	bi	1	261	bi	1	262
1	3270	404	bi	1	684	bi	1	317	bi	1	262	bi	1	263
1	355	140	bi	1	685	bi	1	318	bi	1	263	-		
1	356	80	bi	1	686	bi	1	319	bi	1	264	-		
1	357	273	bi	1	687	bi	1	320	bi	1	265	bi	1	264
1	358	531	bi	1	688	bi	1	321	bi	1	266	bi	1	265
1	359	1004	bi	1	689	bi	1	322	bi	1	267	bi	1	266
1	360	278	bi	1	690	bi	1	323	bi	1	268	bi	1	267
1	361	418	bi	1	691	bi	1	324	bi	1	269	bi	1	268
1	362	313	bi	1	692	bi	1	325	bi	1	270	bi	1	269
1	363	407	bi	1	693	bi	1	326	bi	1	271	bi	1	270
1	364	354	bi	1	694	bi	1	327	bi	1	272	bi	1	271
1	365	452	bi	1	695	bi	1	328	bi	1	273	bi	1	272
1	366	411	bi	1	696	bi	1	329	bi	1	274	bi	1	273
1	367	765	bi	1	697	bi	1	330	bi	1	275	bi	1	274
1	368	55	-			-			-			-		
1	369	450	bi	1	699	bi	1	331	bi	1	276	bi	1	275
1	370	376	bi	1	701	bi	1	332	bi	1	277	bi	1	276
1	371	395	bi	1	702	bi	1	333	bi	1	278	bi	1	277
1	372	172	bi	1	703	bi	1	334	bi	1	279	bi	1	278
1	373	34	-			-			-			-		
1	374	229	bi	1	705	bi	1	336	bi	1	280	bi	1	280
1	375	315	bi	1	706	bi	1	337	bi	1	281	bi	1	281
1	376	214	bi	1	707	bi	1	338	bi	1	282	bi	1	282
1	377	468	bi	1	708	bi	1	339	bi	1	283	bi	1	283
1	378	145	bi	1	709	bi	1	340	bi	1	284	bi	1	284
1	379	212	bi	1	710	bi	1	341	bi	1	285	bi	1	285

1	380	278	bi	1	711	bi	1	342	bi	1	286	bi	1	286
1	381	576	bi	1	712	bi	1	343	bi	1	287	bi	1	287
1	382	678	bi	1	713	bi	1	344	bi	1	288	bi	1	288
1	383	145	bi	1	714	bi	1	345	bi	1	289	bi	1	289
1	384	52	bi	1	715	-			bi	1	290	bi	1	290
1	385	591	bi	1	717	bi	1	346	bi	1	291	bi	1	291
1	386	146	bi	1	718	bi	1	347	bi	1	292	bi	1	292
1	387	385	bi	1	719	bi	1	348	bi	1	293	bi	1	293
1	388	372	bi	1	720	bi	1	349	bi	1	294	bi	1	294
1	389	213	bi	1	721	bi	1	350	bi	1	295	bi	1	295
1	390	393	bi	1	722	bi	1	351	bi	1	296	bi	1	297
1	3271	237	bi	1	723	bi	1	352	bi	1	297	bi	1	298
1	3272	410	bi	1	724	bi	1	353	bi	1	298	bi	1	299
1	391	235	bi	1	725	bi	1	355	bi	1	300	bi	1	300
1	392	506	bi	1	727	bi	1	356	bi	1	301	bi	1	301
1	393	152	bi	1	728	bi	1	357	bi	1	302	bi	1	302
1	394	251	bi	1	729	bi	1	358	bi	1	303	bi	1	303
1	395	216	bi	1	730	bi	1	359	bi	1	304	bi	1	304
1	396	336	bi	1	731	bi	1	360	bi	1	305	bi	1	305
1	397	277	bi	1	732	bi	1	361	bi	1	306	bi	1	306
1	398	263	bi	1	733	bi	1	362	bi	1	307	bi	1	307
1	399	371	-			bi	1	363	bi	1	308	bi	1	308
1	400	336	-			bi	1	364	bi	1	309	bi	1	309
1	401	159	-			bi	1	365	bi	1	310	bi	1	310
1	402	432	-			bi	1	366	bi	1	311	bi	1	311
1	403	291	uni	1	1913	bi	1	367	bi	1	312	bi	1	312
1	404	495	uni	1	898	bi	1	368	bi	1	313	bi	1	313
1	405	429	-			bi	1	369	bi	1	314	bi	1	314
1	406	280	-			bi	1	370	bi	1	315	bi	1	315
1	407	104	-			bi	1	371	bi	1	316	bi	1	316
1	408	307	uni	1	868	bi	1	372	bi	1	317	bi	1	317
1	409	282	-			bi	1	373	bi	1	318	bi	1	319
1	410	67	bi	1	734	bi	1	374	bi	1	319	bi	1	320
1	411	494	bi	1	735	bi	1	375	bi	1	320	bi	1	321
1	412	37	-			-			-			-		
1	413	74	-			bi	1	376	-			bi	1	322
1	414	200	bi	1	736	bi	1	377	bi	1	321	bi	1	323
1	3273	97	bi	1	737	bi	1	378	bi	1	322	bi	1	324
1	415	272	bi	1	738	bi	1	379	bi	1	323	bi	1	325
1	416	486	bi	1	739	bi	1	380	bi	1	324	bi	1	326
1	417	456	bi	1	740	bi	1	381	bi	1	325	bi	1	327
1	418	578	bi	1	741	bi	1	382	bi	1	326	bi	1	328
1	419	268	bi	1	742	bi	1	383	bi	1	327	bi	1	329
1	420	368	bi	1	743	bi	1	384	bi	1	328	bi	1	330
1	421	233	bi	1	744	bi	1	385	bi	1	329	bi	1	331
1	422	509	bi	1	745	bi	1	386	bi	1	330	bi	1	332
1	423	134	bi	1	746	bi	1	387	bi	1	331	bi	1	333
1	424	260	bi	1	747	bi	1	388	bi	1	332	bi	1	334
1	425	268	bi	1	749	bi	1	389	bi	1	333	bi	1	335
1	426	273	bi	1	750	bi	1	390	bi	1	334	bi	1	336
1	427	159	bi	1	752	bi	1	391	bi	1	335	bi	1	337
1	428	284	bi	1	753	bi	1	392	bi	1	336	bi	1	338
1	429	301	bi	1	754	bi	1	393	bi	1	337	bi	1	339
1	430	330	bi	1	756	bi	1	394	bi	1	338	bi	1	340
1	431	140	bi	1	757	bi	1	395	bi	1	339	bi	1	341
1	432	270	bi	1	758	bi	1	396	bi	1	340	bi	1	342
1	433	203	bi	1	759	bi	1	397	bi	1	341	bi	1	343

1	434	327	bi	1	760	bi	1	398	bi	1	342	bi	1	344
1	435	403	bi	1	761	bi	1	399	bi	1	343	bi	1	345
1	436	234	bi	1	762	bi	1	400	bi	1	344	bi	1	346
1	437	234	bi	1	763	bi	1	401	bi	1	345	bi	1	347
1	438	190	bi	1	764	bi	1	402	bi	1	346	bi	1	348
1	439	429	bi	1	765	bi	1	403	bi	1	347	bi	1	349
1	440	322	bi	1	766	bi	1	404	bi	1	348	bi	1	350
1	441	720	bi	1	767	bi	1	405	bi	1	349	bi	1	351
1	442	126	bi	1	768	bi	1	406	bi	1	350	bi	1	352
1	443	75	bi	1	769	bi	1	407	bi	1	351	bi	1	353
1	444	158	bi	1	770	bi	1	409	bi	1	352	bi	1	355
1	445	717	bi	1	771	bi	1	410	bi	1	353	bi	1	356
1	446	46	bi	1	772	bi	1	411	bi	1	354	bi	1	357
1	447	32	-			-			-			-		
1	448	390	bi	1	773	bi	1	412	uni	1	1321	bi	1	358
1	449	77	bi	1	774	bi	1	413	-			bi	1	359
1	450	82	uni	1	528	bi	1	414	uni	1	794	-		
1	451	134	bi	1	775	bi	1	415	uni	1	793	bi	1	360
1	452	1306	uni	3	2942	-			-			uni	1	397
1	453	119	uni	4	3042	-			-			uni	1	396
1	454	129	uni	4	3041	-			-			-		
1	455	87	uni	4	3040	-			-			-		
1	456	293	uni	4	3039	-			-			uni	1	394
1	457	272	uni	4	3038	-			-			uni	1	395
1	458	424	uni	4	3037	uni	1	1563	uni	1	1486	uni	1	193
1	459	206	uni	4	3036	-			-			-		
1	460	123	uni	4	3035	-			-			-		
1	461	105	uni	4	3034	-			-			-		
1	462	325	uni	4	3033	-			-			-		
1	463	87	uni	4	3032	-			-			-		
1	464	131	uni	4	3031	-			-			-		
1	465	161	-			-			-			-		
1	466	153	uni	4	3030	uni	1	1948	uni	1	8	uni	1	8
1	467	831	bi	4	3028	-			uni	1	780	-		
1	468	115	uni	4	3027	-			-			-		
1	469	782	bi	4	3025	-			-			-		
1	470	753	bi	4	3024	-			-			-		
1	471	57	bi	4	3023	-			-			-		
1	472	243	bi	4	3022	-			-			-		
1	473	163	bi	4	3021	-			-			-		
1	474	89	bi	4	3020	bi	3	2996	-			-		
1	475	167	bi	4	3019	-			-			-		
1	476	83	bi	4	3018	-			-			bi	1	1842
1	477	139	bi	4	3017	uni	1	8	uni	1	8	uni	1	8
1	478	195	bi	4	3016	bi	1	1946	-			-		
1	479	89	bi	4	3015	-			-			-		
1	480	92	bi	4	3014	-			-			-		
1	3274	215	uni	1	2520	bi	2	2938	-			uni	1	372
1	3275	232	uni	1	1025	-			-			uni	1	424
1	481	346	bi	1	783	uni	1	1405	uni	1	1330	bi	1	400
1	482	313	bi	1	784	uni	1	1403	uni	1	1328	bi	1	401
1	483	110	uni	4	3038	-			-			uni	1	395
1	484	62	-			-			-			-		
1	3276	236	uni	1	798	uni	2	2938	-			uni	1	410
1	485	325	bi	1	848	bi	1	444	uni	1	2232	bi	1	461
1	3277	215	uni	1	2520	uni	2	2938	-			uni	1	372
1	486	140	uni	3	2976	uni	1	1749	uni	1	1638	uni	1	1746

1	487	67	uni	1	1210	uni	1	691	uni	1	581	uni	1	695
1	488	69	-			-			-			-		
1	489	64	-			-			-			-		
1	490	994	-			-			-			-		
1	491	391	uni	1	2146	-			-			uni	1	1007
1	492	306	bi	1	868	uni	1	372	uni	1	317	uni	1	317
1	493	139	bi	1	869	-			-			-		
1	494	41	bi	1	870	-			-			-		
1	495	222	bi	1	871	-			-			-		
1	496	185	uni	1	874	uni	2	2930	-			uni	1	406
1	3278	218	bi	1	873	uni	2	2930	-			uni	1	456
1	497	273	bi	1	875	uni	1	235	uni	1	233	uni	1	234
1	498	236	bi	1	876	uni	1	67	uni	1	61	uni	1	66
1	3279	501	bi	1	877	uni	1	28	uni	1	25	uni	1	25
1	3280	161	bi	1	878	uni	1	2076	uni	1	1818	uni	1	1963
1	499	255	bi	1	879	-			-			-		
1	500	348	bi	1	880	-			-			-		
1	501	58	bi	1	881	uni	1	869	uni	1	761	uni	1	876
1	502	129	bi	1	882	-			-			-		
1	503	135	bi	1	883	uni	1	2842	uni	1	2450	uni	1	2667
1	504	357	bi	1	884	-			-			-		
1	505	378	bi	1	885	uni	1	2532	uni	1	2147	uni	1	2306
1	506	389	bi	1	891	uni	1	2424	uni	1	2095	uni	1	2251
1	507	689	bi	1	892	-			uni	1	1487	-		
1	508	262	bi	1	893	uni	1	634	uni	1	533	uni	1	335
1	509	271	bi	1	894	uni	1	635	uni	1	534	uni	1	1676
1	510	161	bi	1	895	uni	1	1546	uni	1	1469	uni	1	1612
1	511	136	bi	1	896	uni	1	395	uni	1	339	uni	1	341
1	512	436	bi	1	897	-			-			-		
1	513	497	bi	1	898	uni	1	2823	uni	1	2431	uni	1	2648
1	514	528	uni	1	1006	uni	1	490	uni	1	392	uni	1	516
1	515	97	-			bi	1	1572	-			-		
1	516	98	-			-			-			-		
1	517	51	-			bi	1	1571	-			-		
1	518	82	-			uni	1	1571	-			-		
1	519	559	-			-			-			-		
1	520	677	uni	1	659	uni	1	240	uni	1	238	uni	1	239
1	521	177	-			-			-			-		
1	522	851	uni	1	1584	uni	1	1011	uni	1	937	uni	1	1019
1	523	230	uni	1	1583	uni	1	1010	uni	1	936	uni	1	1018
1	3281	40	-			-			-			-		
1	524	537	-			-			-			-		
1	525	35	-			-			-			-		
1	526	240	bi	1	930	uni	1	1740	uni	1	1629	uni	1	1736
1	527	281	bi	1	931	uni	1	1741	uni	1	1630	uni	1	1737
1	528	317	bi	1	932	uni	1	1742	uni	1	1631	uni	1	1738
1	529	78	bi	1	933	uni	1	827	uni	1	719	uni	1	832
1	530	173	bi	1	934	-			-			-		
1	531	223	bi	1	935	-			-			-		
1	532	475	bi	1	936	uni	1	190	uni	1	187	uni	1	187
1	533	190	bi	1	937	-			-			-		
1	534	585	bi	1	938	uni	1	1478	uni	1	1717	uni	1	1887
1	535	581	bi	1	939	uni	1	2256	uni	1	1996	uni	1	2150
1	536	90	bi	1	940	uni	1	2804	uni	1	2410	uni	1	2624
1	537	60	bi	1	941	uni	1	869	uni	1	761	uni	1	876
1	538	126	bi	1	942	-			-			-		
1	539	50	bi	1	943	uni	1	2805	uni	1	2411	uni	1	2625

1	540	55	bi	1	944	uni	1	2806	uni	1	2412	uni	1	2626
1	541	517	bi	1	946	-			uni	1	1517	-		
1	3282	1570	-			-			-			-		
1	3283	552	-			-			-			-		
1	542	125	-			-			-			-		
1	543	393	uni	1	1025	-			-			uni	1	424
1	3284	35	-			-			-			-		
1	3285	140	-			-			-			-		
1	3286	177	-			-			-			-		
1	544	77	-			-			-			-		
1	545	208	uni	1	1713	uni	1	1140	uni	1	1063	uni	1	1144
1	546	215	-			uni	1	609	uni	1	510	-		
1	3287	141	uni	1	2166	uni	1	1929	-			uni	1	362
1	3288	64	uni	1	825	uni	1	1928	-			uni	1	438
1	547	162	bi	1	860	-			-			-		
1	548	193	uni	1	454	uni	1	76	uni	1	69	uni	1	76
1	549	187	uni	1	863	uni	1	73	uni	1	66	uni	1	73
1	550	156	uni	1	290	uni	1	2821	uni	1	2429	uni	1	2646
1	551	178	-			bi	1	1847	-			uni	1	1835
1	552	142	-			bi	1	1849	-			-		
1	553	424	-			bi	1	1851	-			-		
1	554	38	-			-			-			-		
1	555	132	-			-			-			-		
1	556	92	bi	1	951	-			-			-		
1	557	57	bi	1	952	-			-			-		
1	558	133	bi	1	499	-			-			bi	1	2005
1	559	327	bi	1	954	uni	1	111	uni	1	109	uni	1	111
1	560	293	bi	1	955	-			-			-		
1	561	267	bi	1	956	-			-			-		
1	562	32	bi	1	958	-			-			-		
1	563	57	bi	1	960	-			-			-		
1	564	55	-			-			-			-		
1	3289	135	uni	1	961	uni	1	1544	uni	1	1467	uni	1	1610
1	3290	107	bi	1	962	uni	1	1620	uni	1	1567	uni	1	1676
1	565	89	bi	1	963	-			-			-		
1	566	229	bi	1	964	-			-			-		
1	567	58	-			-			-			-		
1	568	85	-			-			-			-		
1	569	139	bi	1	965	bi	1	451	uni	1	2265	uni	1	2481
1	570	280	bi	1	966	bi	1	452	bi	1	355	bi	1	478
1	571	161	bi	1	967	bi	1	453	bi	1	356	bi	1	479
1	572	482	bi	1	968	bi	1	454	bi	1	357	bi	1	480
1	573	419	bi	1	969	bi	1	455	bi	1	358	bi	1	481
1	574	637	bi	1	970	bi	1	456	bi	1	359	bi	1	482
1	575	473	bi	1	971	bi	1	457	bi	1	360	bi	1	483
1	576	469	bi	1	972	bi	1	458	bi	1	361	bi	1	484
1	577	306	bi	1	974	bi	1	459	bi	1	362	bi	1	485
1	578	150	bi	1	975	bi	1	460	bi	1	363	bi	1	486
1	579	169	bi	1	976	bi	1	461	bi	1	364	bi	1	487
1	580	36	-			-			-			-		
1	581	318	bi	1	977	bi	1	462	bi	1	365	bi	1	488
1	582	121	bi	1	978	bi	1	463	bi	1	366	bi	1	489
1	583	137	bi	1	979	bi	1	464	bi	1	367	bi	1	490
1	584	276	bi	1	980	bi	1	465	bi	1	368	bi	1	491
1	585	323	bi	1	981	bi	1	466	bi	1	369	bi	1	492
1	586	320	bi	1	982	bi	1	467	bi	1	370	bi	1	493
1	587	110	bi	1	983	bi	1	468	bi	1	371	bi	1	494

1	588	206	bi	1	984	bi	1	469	bi	1	372	bi	1	495
1	589	338	bi	1	985	bi	1	470	bi	1	373	bi	1	496
1	3291	172	bi	1	986	bi	1	471	bi	1	374	bi	1	497
1	3292	143	uni	1	986	uni	1	471	uni	1	374	uni	1	497
1	590	109	bi	1	987	bi	1	472	bi	1	375	bi	1	498
1	591	93	bi	1	988	bi	1	473	bi	1	376	bi	1	499
1	592	312	bi	1	989	bi	1	474	bi	1	377	bi	1	500
1	593	199	bi	1	990	bi	1	475	bi	1	378	bi	1	501
1	594	282	bi	1	991	bi	1	476	bi	1	379	bi	1	502
1	595	254	bi	1	992	bi	1	477	bi	1	380	bi	1	503
1	596	467	bi	1	993	bi	1	478	bi	1	381	bi	1	504
1	597	217	bi	1	994	bi	1	479	bi	1	382	bi	1	505
1	598	447	bi	1	996	bi	1	480	bi	1	383	bi	1	506
1	599	603	bi	1	997	bi	1	481	bi	1	384	bi	1	507
1	600	170	bi	1	998	bi	1	482	bi	1	385	bi	1	508
1	601	243	bi	1	999	bi	1	483	bi	1	386	bi	1	509
1	602	157	bi	1	1000	bi	1	484	bi	1	387	bi	1	510
1	603	58	bi	1	1001	bi	1	485	bi	1	388	bi	1	511
1	604	325	bi	1	1002	bi	1	486	bi	1	389	bi	1	512
1	605	191	bi	1	1003	bi	1	487	bi	1	390	bi	1	513
1	606	509	bi	1	1005	bi	1	489	bi	1	391	bi	1	515
1	607	550	bi	1	1006	bi	1	490	bi	1	392	bi	1	516
1	3293	247	bi	1	1007	bi	1	491	bi	1	393	bi	1	517
1	608	472	bi	1	1008	bi	1	492	bi	1	394	bi	1	518
1	609	78	bi	1	1009	bi	1	493	bi	1	395	bi	1	519
1	610	340	bi	1	1010	bi	1	498	bi	1	400	bi	1	520
1	611	318	bi	1	1013	bi	1	499	bi	1	401	bi	1	521
1	612	506	bi	1	1014	bi	1	500	bi	1	402	bi	1	522
1	613	152	bi	1	1015	bi	1	501	bi	1	403	bi	1	523
1	614	503	bi	1	1016	bi	1	502	bi	1	404	bi	1	524
1	615	185	bi	1	1017	bi	1	503	bi	1	405	bi	1	525
1	3294	288	bi	1	1018	bi	1	504	bi	1	406	bi	1	526
1	616	729	bi	1	1019	bi	1	505	bi	1	407	bi	1	527
1	617	114	bi	1	1020	bi	1	506	bi	1	408	bi	1	528
1	618	412	bi	1	1021	bi	1	507	bi	1	409	bi	1	529
1	619	904	bi	1	1022	bi	1	508	bi	1	410	bi	1	530
1	620	315	bi	1	1023	bi	1	509	bi	1	411	bi	1	531
1	621	343	bi	1	1024	bi	1	510	bi	1	412	bi	1	532
1	622	115	bi	1	1026	bi	1	511	bi	1	413	bi	1	533
1	623	142	bi	1	1027	bi	1	512	bi	1	414	bi	1	534
1	624	246	bi	1	1028	bi	1	513	bi	1	415	bi	1	535
1	625	404	bi	1	1029	bi	1	514	bi	1	416	bi	1	536
1	626	260	bi	1	1030	bi	1	515	bi	1	417	bi	1	537
1	627	215	bi	1	1031	bi	1	516	bi	1	418	bi	1	538
1	628	249	bi	1	1032	bi	1	517	bi	1	419	bi	1	539
1	629	309	bi	1	1033	bi	1	518	bi	1	420	bi	1	540
1	630	473	bi	1	1034	bi	1	519	bi	1	421	bi	1	541
1	631	154	bi	1	1035	bi	1	520	bi	1	422	bi	1	542
1	632	326	bi	1	1036	bi	1	521	bi	1	423	bi	1	543
1	633	178	bi	1	1037	bi	1	522	bi	1	424	bi	1	544
1	634	144	bi	1	1038	bi	1	523	bi	1	425	bi	1	545
1	635	382	bi	1	1039	bi	1	524	bi	1	426	bi	1	546
1	636	460	bi	1	1040	bi	1	525	bi	1	427	bi	1	547
1	637	525	bi	1	1041	bi	1	526	bi	1	428	bi	1	548
1	638	148	bi	1	1054	bi	1	527	bi	1	429	bi	1	549
1	639	216	bi	1	1055	bi	1	528	bi	1	430	bi	1	550
1	640	357	bi	1	1056	bi	1	529	bi	1	431	bi	1	551

1	641	105	bi	1	1057	bi	1	530	bi	1	432	bi	1	552
1	642	751	bi	1	1058	bi	1	531	bi	1	433	bi	1	553
1	643	59	bi	1	1059	bi	1	532	-			bi	1	554
1	644	64	bi	1	1060	bi	1	533	bi	1	434	bi	1	555
1	645	89	bi	1	1061	bi	1	534	bi	1	435	bi	1	556
1	646	576	bi	1	1062	bi	1	535	bi	1	436	bi	1	557
1	647	315	bi	1	1063	bi	1	536	bi	1	437	bi	1	558
1	648	246	bi	1	1065	bi	1	537	bi	1	438	bi	1	559
1	649	764	bi	1	1066	bi	1	538	bi	1	439	bi	1	560
1	650	428	bi	1	1068	bi	1	539	bi	1	440	bi	1	561
1	651	221	bi	1	1069	bi	1	540	bi	1	441	bi	1	562
1	652	641	bi	1	1070	bi	1	541	bi	1	442	bi	1	563
1	653	306	bi	1	1071	bi	1	542	bi	1	443	bi	1	564
1	654	251	bi	1	1072	bi	1	543	bi	1	444	bi	1	565
1	655	520	bi	1	1073	bi	1	544	bi	1	445	bi	1	566
1	656	753	bi	1	1074	bi	1	545	bi	1	446	bi	1	567
1	657	677	bi	1	1075	bi	1	546	bi	1	447	bi	1	568
1	658	39	bi	1	1076	bi	1	547	bi	1	448	bi	1	569
1	659	102	bi	1	1077	bi	1	548	bi	1	449	bi	1	570
1	660	490	bi	1	1078	bi	1	549	bi	1	450	bi	1	571
1	661	477	bi	1	1079	bi	1	550	bi	1	451	bi	1	572
1	662	363	bi	1	1080	bi	1	551	bi	1	452	bi	1	573
1	663	458	bi	1	1081	bi	1	552	bi	1	453	bi	1	574
1	664	106	bi	1	1083	bi	1	555	bi	1	455	bi	1	576
1	665	282	bi	1	1084	bi	1	556	bi	1	456	bi	1	577
1	666	340	bi	1	1085	bi	1	557	bi	1	457	bi	1	578
1	667	459	bi	1	1086	bi	1	558	bi	1	458	bi	1	579
1	668	338	bi	1	1087	bi	1	559	bi	1	459	bi	1	580
1	669	310	bi	1	1088	bi	1	560	bi	1	460	bi	1	581
1	670	529	bi	1	1089	bi	1	561	bi	1	461	bi	1	582
1	671	48	bi	1	1090	-			bi	1	462	-		
1	672	227	bi	1	1091	bi	1	562	bi	1	463	bi	1	583
1	673	215	bi	1	1092	bi	1	563	bi	1	464	bi	1	584
1	674	144	bi	1	1093	bi	1	564	bi	1	465	bi	1	585
1	675	129	bi	1	1094	bi	1	565	bi	1	466	bi	1	586
1	676	112	bi	1	1095	bi	1	566	bi	1	467	bi	1	587
1	677	464	bi	1	1096	bi	1	567	bi	1	468	bi	1	588
1	678	127	bi	1	1097	bi	1	568	bi	1	469	bi	1	589
1	679	389	bi	1	1098	bi	1	569	bi	1	470	bi	1	590
1	680	237	bi	1	1099	bi	1	570	bi	1	471	bi	1	591
1	681	317	bi	1	1100	bi	1	571	bi	1	472	bi	1	592
1	682	113	bi	1	1101	bi	1	572	bi	1	473	bi	1	594
1	683	244	bi	1	1102	bi	1	573	bi	1	474	bi	1	595
1	684	260	bi	1	1103	bi	1	574	bi	1	475	bi	1	596
1	685	700	bi	1	1104	bi	1	575	bi	1	476	bi	1	597
1	686	247	bi	1	1105	bi	1	576	bi	1	477	bi	1	598
1	687	365	bi	1	1106	bi	1	577	bi	1	478	bi	1	599
1	688	150	bi	1	1107	bi	1	578	bi	1	479	bi	1	600
1	689	483	bi	1	1108	bi	1	579	bi	1	480	bi	1	601
1	690	635	bi	1	1109	bi	1	580	bi	1	481	bi	1	602
1	691	240	bi	1	1110	bi	1	581	bi	1	482	bi	1	603
1	692	246	bi	1	1111	bi	1	582	bi	1	483	bi	1	604
1	693	723	bi	1	1112	bi	1	583	bi	1	484	bi	1	605
1	694	666	bi	1	1113	bi	1	584	bi	1	485	bi	1	606
1	695	940	bi	1	1114	bi	1	585	bi	1	486	bi	1	607
1	696	189	uni	1	1115	bi	1	586	uni	1	487	bi	1	608
1	697	52	bi	1	1115	uni	1	586	bi	1	487	uni	1	608

1	698	296	bi	1	1116	bi	1	587	bi	1	488	bi	1	609
1	699	332	bi	1	1117	bi	1	588	bi	1	489	bi	1	610
1	700	312	bi	1	1118	bi	1	589	bi	1	490	bi	1	611
1	701	308	bi	1	1119	bi	1	590	bi	1	491	bi	1	612
1	702	173	bi	1	1120	bi	1	591	bi	1	492	bi	1	613
1	703	198	bi	1	1121	bi	1	592	bi	1	493	bi	1	614
1	704	173	bi	1	1122	bi	1	593	bi	1	494	bi	1	615
1	705	135	bi	1	1123	bi	1	594	bi	1	495	bi	1	616
1	706	963	bi	1	1124	bi	1	595	bi	1	496	bi	1	617
1	707	128	bi	1	1125	bi	1	596	bi	1	497	bi	1	618
1	708	170	bi	1	1126	bi	1	597	bi	1	498	bi	1	619
1	709	596	bi	1	1127	bi	1	598	bi	1	499	bi	1	620
1	710	136	bi	1	1128	bi	1	599	bi	1	500	bi	1	621
1	711	67	bi	1	1129	bi	1	600	bi	1	501	bi	1	622
1	712	438	bi	1	1130	bi	1	601	bi	1	502	bi	1	623
1	713	626	bi	1	1131	bi	1	602	bi	1	503	bi	1	624
1	714	395	bi	1	1132	bi	1	603	bi	1	504	bi	1	625
1	715	489	bi	1	1133	bi	1	604	bi	1	505	bi	1	626
1	716	264	bi	1	1134	bi	1	605	bi	1	506	bi	1	627
1	717	221	bi	1	1136	bi	1	606	bi	1	507	bi	1	628
1	718	572	bi	1	1137	bi	1	607	bi	1	508	bi	1	629
1	719	590	bi	1	1138	bi	1	608	bi	1	509	bi	1	630
1	720	202	bi	1	1139	bi	1	609	bi	1	510	bi	1	631
1	721	360	bi	1	1140	bi	1	610	bi	1	511	bi	1	632
1	722	223	bi	1	1141	bi	1	611	bi	1	512	bi	1	633
1	723	188	bi	1	1142	bi	1	612	bi	1	513	bi	1	634
1	724	323	bi	1	1143	bi	1	613	bi	1	514	bi	1	635
1	725	217	bi	1	1144	bi	1	614	bi	1	515	bi	1	636
1	726	342	bi	1	1145	bi	1	615	bi	1	516	bi	1	637
1	727	220	bi	1	1146	bi	1	616	bi	1	517	bi	1	638
1	728	738	bi	1	1148	bi	1	617	bi	1	518	bi	1	639
1	729	805	bi	1	1149	bi	1	618	bi	1	519	bi	1	640
1	730	97	bi	1	1150	bi	1	619	bi	1	520	bi	1	641
1	731	148	bi	1	1151	bi	1	620	bi	1	521	bi	1	642
1	732	277	bi	1	1152	bi	1	623	bi	1	522	bi	1	643
1	733	212	bi	1	1153	bi	1	624	bi	1	523	bi	1	644
1	734	214	bi	1	1154	bi	1	625	bi	1	524	bi	1	645
1	735	544	bi	1	1155	bi	1	626	bi	1	525	bi	1	646
1	736	32	-			-			-			-		
1	737	83	bi	1	1156	bi	1	627	bi	1	526	bi	1	647
1	738	575	uni	1	2471	bi	1	628	bi	1	527	-		
1	739	276	uni	1	2474	bi	1	629	bi	1	528	-		
1	740	388	uni	1	2470	bi	1	630	bi	1	529	-		
1	741	593	uni	1	2144	bi	1	631	bi	1	530	uni	1	1613
1	742	236	bi	1	2157	bi	1	632	bi	1	531	bi	1	370
1	743	309	uni	1	2121	bi	1	633	bi	1	532	uni	1	1612
1	744	264	bi	1	2152	bi	1	634	bi	1	533	uni	1	335
1	745	270	uni	1	2119	bi	1	635	bi	1	534	uni	1	1610
1	746	1005	uni	1	109	bi	1	636	bi	1	535	uni	1	2467
1	747	108	bi	1	1157	bi	1	637	bi	1	536	bi	1	648
1	748	203	bi	1	1158	bi	1	638	bi	1	537	bi	1	649
1	3295	261	bi	1	1159	bi	1	639	bi	1	538	bi	1	650
1	749	227	bi	1	1160	bi	1	640	bi	1	539	bi	1	651
1	750	47	-			-			bi	1	225	-		
1	751	152	bi	1	1161	bi	1	641	bi	1	540	bi	1	652
1	752	210	bi	1	1162	bi	1	642	bi	1	541	bi	1	653
1	753	114	bi	1	1163	bi	1	643	bi	1	542	bi	1	654

1	754	322	bi	1	1164	bi	1	650	bi	1	543	bi	1	655
1	755	640	bi	1	1165	bi	1	651	bi	1	544	bi	1	656
1	756	112	bi	1	1166	bi	1	652	bi	1	545	bi	1	657
1	757	119	bi	1	1167	bi	1	653	bi	1	546	bi	1	658
1	758	119	bi	1	1168	bi	1	654	bi	1	547	bi	1	659
1	759	260	bi	1	1169	bi	1	655	bi	1	548	bi	1	660
1	760	232	bi	1	1170	bi	1	656	bi	1	549	bi	1	661
1	761	438	bi	1	1171	bi	1	657	bi	1	550	bi	1	662
1	762	83	bi	1	1172	-			-			bi	1	663
1	763	363	bi	1	1173	bi	1	658	bi	1	551	bi	1	664
1	764	370	bi	1	1174	bi	1	659	bi	1	552	bi	1	665
1	765	369	bi	1	1175	bi	1	660	bi	1	553	bi	1	666
1	766	248	uni	1	1176	bi	1	2586	bi	1	554	bi	1	2410
1	767	337	bi	1	1178	bi	1	662	bi	1	555	bi	1	668
1	768	343	bi	1	1179	bi	1	663	bi	1	556	bi	1	669
1	769	149	bi	1	1180	bi	1	664	bi	1	557	bi	1	670
1	770	349	bi	1	1181	bi	1	665	bi	1	558	bi	1	671
1	771	31	-			-			-			-		
1	772	458	bi	1	1182	bi	1	666	bi	1	559	bi	1	672
1	773	537	bi	1	1183	bi	1	667	bi	1	560	bi	1	673
1	774	89	-			-			-			-		
1	775	118	bi	1	1186	bi	1	669	bi	1	561	bi	1	675
1	776	372	bi	1	1187	bi	1	670	bi	1	562	bi	1	676
1	777	126	bi	1	1188	bi	1	671	bi	1	563	bi	1	677
1	778	232	bi	1	1190	bi	1	672	bi	1	564	bi	1	678
1	779	432	bi	1	1191	bi	1	673	bi	1	565	bi	1	679
1	780	109	-			bi	1	674	-			-		
1	781	242	bi	1	1192	bi	1	675	bi	1	566	bi	1	680
1	782	493	bi	1	1194	bi	1	677	bi	1	567	bi	1	681
1	783	251	bi	1	1195	bi	1	678	bi	1	568	bi	1	682
1	784	77	bi	1	1196	bi	1	679	bi	1	569	bi	1	683
1	785	889	bi	1	1197	bi	1	680	bi	1	570	bi	1	684
1	786	390	bi	1	1198	bi	1	681	bi	1	571	bi	1	685
1	787	608	bi	1	1199	bi	1	682	bi	1	572	bi	1	686
1	788	155	bi	1	1202	bi	1	683	bi	1	573	bi	1	687
1	789	221	bi	1	1203	bi	1	684	bi	1	574	bi	1	688
1	790	308	bi	1	1204	bi	1	685	bi	1	575	bi	1	689
1	791	212	bi	1	1205	bi	1	686	bi	1	576	bi	1	690
1	792	398	bi	1	1206	bi	1	687	bi	1	577	bi	1	691
1	793	297	bi	1	1208	bi	1	689	bi	1	579	bi	1	693
1	794	344	bi	1	1209	bi	1	690	bi	1	580	bi	1	694
1	795	83	bi	1	1210	bi	1	691	bi	1	581	bi	1	695
1	796	882	bi	1	1212	bi	1	692	bi	1	582	bi	1	696
1	797	669	bi	1	1213	bi	1	693	bi	1	583	bi	1	697
1	798	233	bi	1	1214	bi	1	694	bi	1	584	bi	1	698
1	799	702	bi	1	1215	bi	1	695	bi	1	585	bi	1	699
1	800	475	bi	1	1216	bi	1	696	bi	1	586	bi	1	701
1	801	304	bi	1	1217	bi	1	697	bi	1	587	bi	1	702
1	802	883	bi	1	1218	bi	1	698	bi	1	588	bi	1	703
1	803	281	bi	1	1219	bi	1	699	bi	1	589	bi	1	704
1	804	200	bi	1	1220	bi	1	700	bi	1	590	bi	1	705
1	805	161	bi	1	1221	bi	1	701	bi	1	591	bi	1	706
1	806	470	bi	1	1222	bi	1	702	bi	1	592	bi	1	707
1	807	311	bi	1	1223	bi	1	703	bi	1	593	bi	1	708
1	808	358	bi	1	1224	bi	1	704	bi	1	594	bi	1	709
1	809	507	bi	1	1225	bi	1	705	bi	1	595	bi	1	710
1	810	528	bi	1	1226	bi	1	706	bi	1	596	bi	1	711

1	811	421	bi	1	1227	bi	1	708	bi	1	597	bi	1	712
1	812	689	bi	1	1228	bi	1	709	bi	1	598	bi	1	714
1	813	283	bi	1	1229	bi	1	710	bi	1	599	bi	1	715
1	814	104	bi	1	1230	bi	1	711	bi	1	600	bi	1	716
1	815	385	bi	1	1231	bi	1	712	bi	1	601	bi	1	717
1	816	241	bi	1	1232	bi	1	713	bi	1	602	bi	1	718
1	817	488	bi	1	1233	bi	1	714	bi	1	603	bi	1	719
1	818	363	bi	1	1235	bi	1	716	bi	1	605	bi	1	721
1	819	382	bi	1	1236	bi	1	717	bi	1	606	bi	1	722
1	820	126	bi	1	1237	bi	1	718	bi	1	607	bi	1	723
1	821	80	uni	1	2129	-			-			bi	1	2654
1	822	96	bi	1	1238	bi	1	719	bi	1	608	bi	1	724
1	823	849	bi	1	1239	bi	1	720	bi	1	609	bi	1	725
1	824	348	bi	1	1240	bi	1	721	bi	1	610	bi	1	726
1	825	369	bi	1	1241	bi	1	722	bi	1	611	bi	1	727
1	826	332	bi	1	1242	bi	1	723	bi	1	612	bi	1	728
1	827	315	bi	1	1243	bi	1	724	bi	1	613	bi	1	729
1	828	212	bi	1	1244	bi	1	725	bi	1	614	bi	1	730
1	829	358	bi	1	1245	bi	1	726	bi	1	615	bi	1	731
1	830	557	bi	1	1246	bi	1	727	bi	1	616	bi	1	732
1	831	117	bi	1	1247	bi	1	728	bi	1	617	bi	1	733
1	832	314	bi	1	1248	bi	1	729	bi	1	618	bi	1	734
1	833	351	bi	1	1249	bi	1	730	bi	1	619	bi	1	735
1	834	353	bi	1	1250	bi	1	731	bi	1	620	bi	1	736
1	835	319	bi	1	1251	bi	1	732	bi	1	621	bi	1	737
1	836	357	bi	1	1252	bi	1	733	bi	1	622	bi	1	738
1	837	174	bi	1	1253	bi	1	734	bi	1	623	bi	1	739
1	838	67	bi	1	1254	bi	1	735	bi	1	624	bi	1	740
1	839	120	bi	1	1255	bi	1	736	bi	1	625	bi	1	741
1	840	117	bi	1	1256	bi	1	737	bi	1	626	bi	1	742
1	841	120	bi	1	1257	bi	1	738	bi	1	627	bi	1	743
1	842	145	bi	1	1258	bi	1	739	bi	1	628	bi	1	744
1	843	172	bi	1	1259	bi	1	740	bi	1	629	bi	1	745
1	844	392	bi	1	1260	bi	1	741	bi	1	630	bi	1	746
1	845	343	bi	1	1261	bi	1	742	bi	1	631	bi	1	747
1	846	277	bi	1	1262	bi	1	743	bi	1	632	bi	1	748
1	847	291	bi	1	1263	bi	1	744	bi	1	633	bi	1	749
1	848	75	bi	1	1264	bi	1	745	bi	1	634	bi	1	750
1	849	225	bi	1	1265	bi	1	746	bi	1	635	bi	1	751
1	850	342	bi	1	1266	bi	1	747	bi	1	636	bi	1	752
1	851	288	bi	1	1267	bi	1	748	bi	1	637	bi	1	753
1	852	464	bi	1	1268	bi	1	749	bi	1	638	bi	1	754
1	853	670	bi	1	1269	bi	1	750	bi	1	639	bi	1	755
1	854	92	bi	1	1270	bi	1	751	-			-		
1	855	202	bi	1	1271	bi	1	752	bi	1	640	bi	1	757
1	856	733	bi	1	1272	bi	1	753	bi	1	641	bi	1	758
1	857	257	bi	1	1273	bi	1	754	bi	1	642	bi	1	759
1	858	191	bi	1	1274	bi	1	755	bi	1	643	bi	1	760
1	859	296	bi	1	1275	bi	1	756	bi	1	644	bi	1	761
1	860	206	bi	1	1276	bi	1	757	bi	1	645	bi	1	762
1	861	368	bi	1	1277	bi	1	758	bi	1	646	bi	1	763
1	862	141	bi	1	1278	bi	1	759	bi	1	647	bi	1	764
1	863	180	bi	1	1279	bi	1	760	bi	1	648	bi	1	765
1	864	589	bi	1	1280	bi	1	761	bi	1	649	bi	1	766
1	865	576	bi	1	1281	bi	1	762	bi	1	650	bi	1	767
1	866	262	bi	1	1282	bi	1	763	bi	1	651	bi	1	768
1	867	403	bi	1	1283	bi	1	764	bi	1	652	bi	1	769

1	868	188	bi	1	1284	bi	1	765	bi	1	653	bi	1	770
1	869	175	bi	1	1285	bi	1	766	bi	1	654	bi	1	771
1	870	290	bi	1	1286	bi	1	767	bi	1	655	bi	1	772
1	871	227	bi	1	1287	bi	1	768	bi	1	656	bi	1	773
1	872	328	bi	1	1288	bi	1	769	bi	1	657	bi	1	774
1	873	160	bi	1	1289	bi	1	770	bi	1	658	bi	1	775
1	874	174	bi	1	1290	bi	1	771	bi	1	659	bi	1	776
1	875	70	bi	1	1291	bi	1	772	bi	1	660	bi	1	777
1	876	337	bi	1	1292	bi	1	773	bi	1	661	bi	1	778
1	877	336	bi	1	1293	bi	1	774	bi	1	662	bi	1	779
1	878	223	bi	1	1294	bi	1	775	bi	1	663	bi	1	780
1	879	765	bi	1	1295	bi	1	776	bi	1	664	bi	1	781
1	880	723	bi	1	1296	bi	1	777	bi	1	665	bi	1	782
1	881	45	-			-			uni	1	225	-		
1	882	275	bi	1	1297	bi	1	778	bi	1	666	bi	1	783
1	883	272	bi	1	1298	bi	1	779	bi	1	667	bi	1	784
1	884	321	bi	1	1299	bi	1	780	bi	1	668	bi	1	785
1	885	100	bi	1	1300	bi	1	781	bi	1	669	bi	1	786
1	886	97	bi	1	1301	bi	1	782	bi	1	670	bi	1	787
1	887	238	-			-			-			-		
1	888	259	bi	1	1302	bi	1	783	bi	1	671	bi	1	788
1	889	631	uni	1	765	uni	1	403	uni	1	347	uni	1	349
1	890	103	bi	1	1311	bi	1	792	bi	1	683	bi	1	795
1	891	115	bi	1	1312	bi	1	793	bi	1	684	bi	1	796
1	892	96	bi	1	1313	bi	1	794	bi	1	685	bi	1	797
1	893	151	bi	1	1368	bi	1	795	bi	1	686	bi	1	798
1	894	272	bi	1	1369	bi	1	796	bi	1	687	bi	1	799
1	895	355	bi	1	1370	bi	1	797	bi	1	688	bi	1	800
1	896	107	bi	1	1371	bi	1	798	bi	1	689	bi	1	801
1	897	144	bi	1	1372	bi	1	799	bi	1	690	bi	1	802
1	898	155	bi	1	1373	bi	1	800	bi	1	691	bi	1	803
1	899	282	bi	1	1374	bi	1	801	bi	1	692	bi	1	804
1	900	449	bi	1	1375	bi	1	802	bi	1	693	bi	1	805
1	901	77	bi	1	1376	bi	1	803	bi	1	694	bi	1	806
1	902	294	bi	1	1377	bi	1	804	bi	1	695	bi	1	807
1	903	272	bi	1	1378	bi	1	805	bi	1	696	bi	1	808
1	904	150	bi	1	1379	bi	1	806	bi	1	697	bi	1	811
1	905	558	bi	1	1380	bi	1	807	bi	1	698	bi	1	812
1	906	57	bi	1	1381	bi	1	808	bi	1	699	bi	1	813
1	907	322	bi	1	1382	bi	1	809	bi	1	700	bi	1	814
1	908	131	-			-			bi	1	701	-		
1	909	144	bi	1	1383	bi	1	810	bi	1	702	bi	1	815
1	910	320	bi	1	1384	bi	1	811	bi	1	703	bi	1	816
1	911	136	bi	1	1385	bi	1	812	bi	1	704	bi	1	817
1	912	743	bi	1	1386	bi	1	813	bi	1	705	bi	1	818
1	913	322	bi	1	1387	bi	1	814	bi	1	706	bi	1	819
1	914	457	bi	1	1388	bi	1	815	bi	1	707	bi	1	820
1	915	364	bi	1	1389	bi	1	816	bi	1	708	bi	1	821
1	916	375	bi	1	1390	bi	1	817	bi	1	709	bi	1	822
1	917	441	bi	1	1391	bi	1	818	bi	1	710	bi	1	823
1	918	411	bi	1	1392	bi	1	819	bi	1	711	bi	1	824
1	919	226	bi	1	1393	bi	1	820	bi	1	712	bi	1	825
1	920	215	bi	1	1394	bi	1	821	bi	1	713	bi	1	826
1	921	70	bi	1	1395	bi	1	822	bi	1	714	bi	1	827
1	922	261	bi	1	1396	bi	1	823	bi	1	715	bi	1	828
1	923	234	bi	1	1397	bi	1	824	bi	1	716	bi	1	829
1	924	927	bi	1	1398	bi	1	825	bi	1	717	bi	1	830

1	925	508	bi	1	1399	bi	1	826	bi	1	718	bi	1	831
1	926	223	bi	1	1400	bi	1	827	bi	1	719	bi	1	832
1	927	123	bi	1	1401	bi	1	828	bi	1	720	bi	1	833
1	3296	194	bi	1	1402	bi	1	829	bi	1	721	bi	1	834
1	928	333	bi	1	1403	bi	1	830	bi	1	722	bi	1	835
1	929	434	bi	1	1404	bi	1	831	bi	1	723	bi	1	836
1	930	902	bi	1	1405	bi	1	832	bi	1	724	bi	1	837
1	931	106	bi	1	1406	bi	1	833	bi	1	725	bi	1	839
1	932	488	bi	1	1407	bi	1	834	bi	1	726	bi	1	840
1	933	73	bi	1	1408	bi	1	835	bi	1	727	bi	1	841
1	934	65	bi	1	1409	bi	1	836	bi	1	728	bi	1	842
1	935	137	bi	1	1410	bi	1	837	bi	1	729	bi	1	843
1	936	109	bi	1	1411	bi	1	838	bi	1	730	bi	1	844
1	937	107	bi	1	1412	bi	1	839	bi	1	731	bi	1	845
1	938	426	bi	1	1413	bi	1	840	bi	1	732	bi	1	846
1	939	465	bi	1	1414	bi	1	841	bi	1	733	bi	1	847
1	940	398	bi	1	1415	bi	1	842	bi	1	734	bi	1	848
1	941	40	bi	1	1416	bi	1	843	bi	1	735	bi	1	849
1	942	111	bi	1	1417	bi	1	844	bi	1	736	bi	1	850
1	943	882	bi	1	1418	bi	1	845	bi	1	737	bi	1	851
1	944	210	bi	1	1419	bi	1	846	bi	1	738	bi	1	852
1	945	271	bi	1	1420	bi	1	847	bi	1	739	bi	1	853
1	3297	844	bi	1	1421	bi	1	848	bi	1	740	bi	1	854
1	946	245	bi	1	1422	bi	1	849	bi	1	741	bi	1	855
1	947	195	bi	1	1423	bi	1	850	bi	1	742	bi	1	856
1	948	352	bi	1	1424	bi	1	851	bi	1	743	bi	1	857
1	949	219	uni	1	2791	uni	1	2335	bi	1	744	bi	1	858
1	950	746	bi	1	1425	bi	1	852	bi	1	745	bi	1	860
1	951	730	bi	1	1426	bi	1	853	bi	1	746	bi	1	861
1	952	310	bi	1	1427	bi	1	854	bi	1	747	bi	1	862
1	953	218	bi	1	1432	bi	1	855	bi	1	748	bi	1	863
1	954	134	bi	1	1433	bi	1	856	bi	1	749	bi	1	864
1	955	537	bi	1	1434	bi	1	857	bi	1	750	bi	1	865
1	956	362	bi	1	1435	bi	1	858	bi	1	751	bi	1	866
1	957	268	bi	1	1436	bi	1	859	bi	1	752	bi	1	867
1	958	210	bi	1	1438	bi	1	861	bi	1	753	bi	1	868
1	959	469	bi	1	1439	bi	1	862	bi	1	754	bi	1	869
1	960	399	bi	1	1440	bi	1	863	bi	1	755	bi	1	870
1	961	62	bi	1	1441	bi	1	864	bi	1	756	bi	1	871
1	962	1110	bi	1	1442	bi	1	865	bi	1	757	bi	1	872
1	963	321	bi	1	1443	bi	1	866	bi	1	758	bi	1	873
1	964	586	bi	1	1444	bi	1	867	bi	1	759	bi	1	874
1	965	190	bi	1	1445	bi	1	868	bi	1	760	bi	1	875
1	966	60	bi	1	1446	bi	1	869	bi	1	761	bi	1	876
1	967	474	bi	1	1447	bi	1	870	bi	1	762	bi	1	877
1	968	229	bi	1	1448	bi	1	871	bi	1	763	bi	1	878
1	969	503	bi	1	1449	bi	1	872	bi	1	764	bi	1	879
1	970	55	-			-			-			-		
1	971	270	bi	1	1451	bi	1	874	bi	1	808	bi	1	880
1	972	586	bi	1	1452	bi	1	875	bi	1	809	bi	1	881
1	973	171	bi	1	1454	bi	1	877	bi	1	810	bi	1	882
1	974	32	-			-			-			-		
1	975	533	bi	1	1455	bi	1	878	bi	1	811	bi	1	883
1	976	142	bi	1	1456	bi	1	879	bi	1	812	bi	1	884
1	977	557	bi	1	1457	bi	1	880	bi	1	813	bi	1	885
1	978	554	bi	1	1458	bi	1	881	bi	1	814	bi	1	887
1	979	408	bi	1	1459	bi	1	882	bi	1	815	bi	1	888

1	980	406	bi	1	1460	bi	1	883	bi	1	816	bi	1	889
1	981	160	bi	1	1461	bi	1	884	bi	1	817	bi	1	890
1	3298	124	bi	1	1462	bi	1	885	bi	1	818	bi	1	891
1	982	36	-			-			-			-		
1	983	213	bi	1	1463	bi	1	886	bi	1	819	bi	1	892
1	984	68	bi	1	1464	bi	1	887	bi	1	820	bi	1	893
1	985	346	bi	1	1465	bi	1	888	bi	1	821	bi	1	894
1	986	388	bi	1	1466	bi	1	889	bi	1	822	bi	1	895
1	987	330	bi	1	1467	bi	1	890	bi	1	823	bi	1	896
1	988	498	bi	1	1468	bi	1	891	bi	1	824	bi	1	897
1	989	335	bi	1	1469	bi	1	892	bi	1	825	bi	1	898
1	990	165	bi	1	1470	bi	1	893	bi	1	826	bi	1	899
1	991	91	bi	1	1471	bi	1	894	bi	1	827	bi	1	900
1	992	169	bi	1	1472	bi	1	895	bi	1	828	bi	1	901
1	993	262	-			-			bi	1	829	bi	1	902
1	994	150	bi	1	1473	bi	1	896	bi	1	830	bi	1	903
1	995	385	bi	1	1474	bi	1	897	bi	1	831	bi	1	904
1	996	439	bi	1	1476	bi	1	898	bi	1	832	bi	1	905
1	997	130	bi	1	1477	bi	1	899	bi	1	833	bi	1	906
1	998	61	bi	1	1478	bi	1	900	bi	1	834	bi	1	907
1	999	102	bi	1	1479	bi	1	901	bi	1	835	bi	1	908
1	1000	144	bi	1	1480	bi	1	902	bi	1	836	bi	1	909
1	1001	81	bi	1	1481	bi	1	903	bi	1	837	bi	1	910
1	1002	149	bi	1	1482	bi	1	904	bi	1	838	bi	1	911
1	1003	238	bi	1	1483	bi	1	906	bi	1	839	bi	1	913
1	1004	132	bi	1	1484	bi	1	907	bi	1	840	bi	1	914
1	1005	474	bi	1	1485	bi	1	908	bi	1	841	bi	1	915
1	1006	1104	bi	1	1486	bi	1	909	bi	1	842	bi	1	916
1	1007	477	bi	1	1487	bi	1	910	bi	1	843	bi	1	917
1	1008	626	bi	1	1488	bi	1	911	bi	1	844	bi	1	919
1	1009	285	bi	1	1489	bi	1	912	bi	1	845	bi	1	920
1	1010	98	-			bi	1	913	-			bi	1	922
1	1011	68	bi	1	1490	bi	1	914	bi	1	846	bi	1	923
1	1012	171	bi	1	1491	bi	1	915	bi	1	847	bi	1	924
1	1013	102	bi	1	1493	bi	1	921	bi	1	848	bi	1	926
1	1014	675	bi	1	1494	bi	1	922	bi	1	849	bi	1	927
1	1015	532	bi	1	1495	bi	1	923	bi	1	850	bi	1	928
1	1016	245	bi	1	1496	bi	1	924	bi	1	851	bi	1	929
1	1017	134	bi	1	1497	bi	1	925	bi	1	852	bi	1	930
1	1018	451	bi	1	1498	bi	1	926	bi	1	853	bi	1	931
1	1019	62	bi	1	1499	bi	1	927	bi	1	854	bi	1	932
1	1020	102	bi	1	1500	bi	1	928	bi	1	855	bi	1	933
1	1021	43	bi	1	1501	bi	1	929	bi	1	856	bi	1	934
1	1022	52	-			-			-			-		
1	1023	254	bi	1	1502	bi	1	930	bi	1	857	bi	1	935
1	1024	491	bi	1	1503	bi	1	931	bi	1	858	bi	1	936
1	1025	240	bi	1	1504	bi	1	932	bi	1	859	bi	1	937
1	1026	183	bi	1	1506	bi	1	934	bi	1	860	bi	1	939
1	1027	1197	bi	1	1507	bi	1	935	bi	1	861	bi	1	940
1	1028	1265	bi	1	1508	bi	1	936	bi	1	862	bi	1	941
1	1029	115	bi	1	1509	bi	1	937	bi	1	863	bi	1	942
1	1030	349	bi	1	1510	bi	1	938	bi	1	864	bi	1	943
1	1031	808	bi	1	1511	bi	1	939	bi	1	865	bi	1	944
1	1032	222	bi	1	1512	bi	1	940	bi	1	866	bi	1	945
1	1033	238	bi	1	1513	bi	1	941	bi	1	867	bi	1	946
1	1034	276	bi	1	1514	bi	1	942	bi	1	868	bi	1	947
1	1035	244	bi	1	1515	bi	1	943	bi	1	869	bi	1	948

1	1036	274	bi	1	1516	bi	1	944	bi	1	870	bi	1	949
1	1037	452	bi	1	1517	bi	1	945	bi	1	871	bi	1	950
1	1038	171	bi	1	1518	bi	1	946	bi	1	872	bi	1	951
1	1039	255	bi	1	1519	bi	1	947	bi	1	873	bi	1	952
1	1040	356	bi	1	1520	bi	1	948	bi	1	874	bi	1	953
1	1041	153	bi	1	1521	bi	1	949	bi	1	875	bi	1	954
1	1042	496	bi	1	1522	bi	1	950	bi	1	876	bi	1	955
1	1043	100	bi	1	1523	bi	1	951	bi	1	877	bi	1	956
1	1044	213	bi	1	1524	bi	1	952	bi	1	878	bi	1	957
1	1045	287	bi	1	1525	bi	1	953	bi	1	879	bi	1	958
1	1046	237	bi	1	1526	bi	1	954	bi	1	880	bi	1	959
1	1047	163	bi	1	1527	bi	1	955	bi	1	881	bi	1	960
1	1048	234	bi	1	1528	bi	1	956	bi	1	882	bi	1	961
1	1049	390	bi	1	1529	bi	1	957	bi	1	883	bi	1	962
1	1050	307	bi	1	1530	bi	1	958	bi	1	884	bi	1	963
1	1051	49	bi	1	1531	-			bi	1	885	-		
1	1052	92	-			bi	1	959	-			bi	1	964
1	1053	275	bi	1	1532	bi	1	960	bi	1	886	bi	1	965
1	1054	241	bi	1	1533	bi	1	961	bi	1	887	bi	1	966
1	1055	125	bi	1	1534	bi	1	962	bi	1	888	bi	1	967
1	1056	149	bi	1	1535	bi	1	963	bi	1	889	bi	1	968
1	1057	271	bi	1	1536	bi	1	964	bi	1	890	bi	1	969
1	1058	457	bi	1	1537	bi	1	965	bi	1	891	bi	1	970
1	1059	284	bi	1	1538	bi	1	966	bi	1	892	bi	1	971
1	1060	146	bi	1	1539	bi	1	967	bi	1	893	bi	1	972
1	1061	209	bi	1	1540	bi	1	968	bi	1	894	bi	1	973
1	1062	537	bi	1	1541	bi	1	969	bi	1	895	bi	1	974
1	1063	779	bi	1	1542	bi	1	970	bi	1	896	bi	1	975
1	1064	206	bi	1	1543	bi	1	971	bi	1	897	bi	1	976
1	1065	181	bi	1	1544	bi	1	972	bi	1	898	bi	1	977
1	1066	137	bi	1	1545	bi	1	973	bi	1	899	bi	1	978
1	1067	388	bi	1	1547	bi	1	974	bi	1	900	bi	1	980
1	1068	499	bi	1	1548	bi	1	975	bi	1	901	bi	1	981
1	1069	239	bi	1	1549	bi	1	976	bi	1	902	bi	1	982
1	1070	216	bi	1	1550	bi	1	977	bi	1	903	bi	1	983
1	1071	255	bi	1	1551	bi	1	978	bi	1	904	bi	1	984
1	1072	433	bi	1	1552	bi	1	979	bi	1	905	bi	1	985
1	1073	308	bi	1	1553	bi	1	980	bi	1	906	bi	1	986
1	1074	110	bi	1	1554	bi	1	981	bi	1	907	bi	1	987
1	1075	452	bi	1	1555	bi	1	982	bi	1	908	bi	1	988
1	1076	173	bi	1	1556	bi	1	983	bi	1	910	bi	1	989
1	1077	794	bi	1	1557	bi	1	984	bi	1	911	bi	1	990
1	1078	322	bi	1	1558	bi	1	985	bi	1	912	bi	1	991
1	1079	228	bi	1	1559	bi	1	986	bi	1	913	bi	1	992
1	1080	165	bi	1	1560	bi	1	987	bi	1	914	bi	1	993
1	1081	319	bi	1	1561	bi	1	988	bi	1	915	bi	1	994
1	1082	290	bi	1	1562	bi	1	989	bi	1	916	bi	1	995
1	1083	52	bi	1	1563	bi	1	990	-			bi	1	996
1	1084	431	bi	1	1564	bi	1	991	bi	1	917	bi	1	997
1	1085	431	bi	1	1565	bi	1	992	bi	1	918	bi	1	998
1	1086	90	bi	1	1566	bi	1	993	bi	1	919	bi	1	999
1	1087	371	-			-			uni	1	1664	-		
1	1088	249	-			-			-			-		
1	1089	198	-			-			-			-		
1	1090	133	-			-			bi	1	1663	-		
1	1091	355	bi	1	1567	bi	1	994	bi	1	920	bi	1	1000
1	1092	71	bi	1	1568	bi	1	995	bi	1	921	bi	1	1001

1	1093	293	bi	1	1569	bi	1	996	bi	1	922	bi	1	1002
1	1094	83	bi	1	1570	bi	1	997	bi	1	923	bi	1	1003
1	1095	245	bi	1	1571	bi	1	998	bi	1	924	bi	1	1004
1	1096	153	bi	1	1572	bi	1	999	bi	1	925	bi	1	1005
1	1097	356	bi	1	1573	bi	1	1000	bi	1	926	bi	1	1008
1	1098	291	bi	1	1574	bi	1	1001	bi	1	927	bi	1	1009
1	1099	563	bi	1	1575	bi	1	1002	bi	1	928	bi	1	1010
1	1100	461	bi	1	1576	bi	1	1003	bi	1	929	bi	1	1011
1	1101	210	bi	1	1577	bi	1	1004	bi	1	930	bi	1	1012
1	1102	257	bi	1	1578	bi	1	1005	bi	1	931	bi	1	1013
1	1103	218	bi	1	1579	bi	1	1006	bi	1	932	bi	1	1014
1	1104	190	bi	1	1580	bi	1	1007	bi	1	933	bi	1	1015
1	1105	287	bi	1	1581	bi	1	1008	bi	1	934	bi	1	1016
1	1106	222	bi	1	1582	bi	1	1009	bi	1	935	bi	1	1017
1	1107	235	bi	1	1583	bi	1	1010	bi	1	936	bi	1	1018
1	1108	610	bi	1	1584	bi	1	1011	bi	1	937	bi	1	1019
1	1109	437	bi	1	1585	bi	1	1012	bi	1	938	bi	1	1020
1	1110	285	bi	1	1586	bi	1	1013	bi	1	939	bi	1	1021
1	1111	269	bi	1	1587	bi	1	1014	bi	1	940	bi	1	1022
1	1112	305	bi	1	1588	bi	1	1015	bi	1	941	bi	1	1023
1	1113	289	bi	1	1589	bi	1	1016	bi	1	942	bi	1	1024
1	1114	164	bi	1	1590	bi	1	1017	bi	1	943	bi	1	1025
1	1115	251	bi	1	1591	bi	1	1018	bi	1	944	bi	1	1026
1	1116	90	bi	1	1592	bi	1	1019	bi	1	945	bi	1	1027
1	1117	141	bi	1	1593	bi	1	1020	bi	1	946	bi	1	1028
1	1118	102	bi	1	1594	bi	1	1021	bi	1	947	bi	1	1029
1	3299	121	uni	1	1595	uni	1	1022	uni	1	948	uni	1	1030
1	3300	154	bi	1	1595	bi	1	1022	bi	1	948	bi	1	1030
1	1119	390	bi	1	1596	bi	1	1023	bi	1	949	bi	1	1031
1	1120	442	bi	1	1597	bi	1	1024	bi	1	950	bi	1	1032
1	1121	517	bi	1	1598	bi	1	1025	bi	1	951	bi	1	1033
1	1122	231	bi	1	1599	bi	1	1026	bi	1	952	bi	1	1034
1	1123	448	bi	1	1600	bi	1	1027	bi	1	953	bi	1	1035
1	1124	304	bi	1	1601	bi	1	1028	bi	1	954	bi	1	1036
1	1125	551	bi	1	1602	bi	1	1029	bi	1	955	bi	1	1037
1	1126	235	bi	1	1603	bi	1	1030	bi	1	956	bi	1	1038
1	1127	361	bi	1	1605	bi	1	1031	bi	1	957	bi	1	1039
1	1128	240	bi	1	1606	bi	1	1032	bi	1	958	bi	1	1040
1	1129	135	bi	1	1607	bi	1	1033	bi	1	959	bi	1	1041
1	1130	279	bi	1	1608	bi	1	1034	bi	1	960	bi	1	1042
1	1131	260	bi	1	1609	bi	1	1035	bi	1	961	bi	1	1043
1	1132	347	bi	1	1610	bi	1	1036	bi	1	962	bi	1	1044
1	1133	355	bi	1	1611	bi	1	1037	bi	1	963	bi	1	1045
1	1134	580	bi	1	1612	bi	1	1038	bi	1	964	bi	1	1046
1	1135	443	bi	1	1613	bi	1	1039	bi	1	965	bi	1	1047
1	1136	295	bi	1	1614	bi	1	1040	bi	1	966	bi	1	1048
1	1137	312	bi	1	1615	bi	1	1041	bi	1	967	bi	1	1049
1	1138	203	bi	1	1616	bi	1	1042	bi	1	968	bi	1	1050
1	1139	419	bi	1	1617	bi	1	1043	bi	1	969	bi	1	1051
1	1140	497	bi	1	1618	bi	1	1044	bi	1	970	bi	1	1052
1	1141	228	bi	1	1619	bi	1	1045	bi	1	971	bi	1	1053
1	1142	35	-			-			-			-		
1	1143	275	bi	1	1620	bi	1	1046	bi	1	972	bi	1	1054
1	1144	304	bi	1	1621	bi	1	1047	bi	1	973	bi	1	1055
1	1145	294	bi	1	1622	bi	1	1048	bi	1	974	bi	1	1056
1	1146	533	bi	1	1623	bi	1	1049	bi	1	975	bi	1	1057
1	1147	198	bi	1	1624	bi	1	1050	bi	1	976	bi	1	1058

1	1148	306	bi	1	1625	bi	1	1051	bi	1	977	bi	1	1059
1	1149	255	bi	1	1626	bi	1	1052	bi	1	978	bi	1	1060
1	1150	717	bi	1	1627	bi	1	1053	bi	1	979	bi	1	1061
1	1151	1092	bi	1	1628	bi	1	1054	bi	1	980	bi	1	1062
1	1152	322	bi	1	1629	bi	1	1055	bi	1	981	bi	1	1063
1	1153	177	bi	1	1630	bi	1	1056	bi	1	982	bi	1	1064
1	1154	238	bi	1	1631	bi	1	1057	bi	1	983	bi	1	1065
1	1155	478	bi	1	1632	bi	1	1058	bi	1	984	bi	1	1066
1	1156	350	bi	1	1633	bi	1	1059	bi	1	985	bi	1	1067
1	1157	153	bi	1	1634	bi	1	1060	bi	1	986	bi	1	1068
1	1158	114	bi	1	1635	bi	1	1061	bi	1	987	bi	1	1069
1	1159	88	bi	1	1636	bi	1	1062	bi	1	988	bi	1	1070
1	1160	571	bi	1	1637	bi	1	1063	bi	1	989	bi	1	1071
1	1161	224	bi	1	1638	bi	1	1064	bi	1	990	bi	1	1072
1	1162	36	-			-			-			-		
1	1163	330	bi	1	1640	bi	1	1066	bi	1	992	bi	1	1073
1	1164	296	bi	1	1641	bi	1	1067	bi	1	993	bi	1	1074
1	1165	256	bi	1	1642	bi	1	1068	bi	1	994	bi	1	1075
1	3301	154	bi	1	1645	bi	1	1071	bi	1	995	bi	1	1076
1	3302	259	bi	1	1646	bi	1	1072	bi	1	996	bi	1	1077
1	1166	69	bi	1	1647	bi	1	1073	bi	1	997	bi	1	1078
1	1167	260	bi	1	1648	bi	1	1074	bi	1	998	bi	1	1079
1	1168	240	bi	1	1649	bi	1	1075	bi	1	999	bi	1	1080
1	1169	490	bi	1	1650	bi	1	1076	bi	1	1000	bi	1	1081
1	1170	184	bi	1	1651	bi	1	1077	bi	1	1001	bi	1	1082
1	1171	234	bi	1	1652	bi	1	1078	bi	1	1002	bi	1	1083
1	1172	703	bi	1	1653	bi	1	1079	bi	1	1003	bi	1	1084
1	1173	391	bi	1	1654	bi	1	1080	bi	1	1004	bi	1	1085
1	1174	34	-			-			-			-		
1	1175	306	bi	1	1655	bi	1	1081	bi	1	1005	bi	1	1086
1	1176	807	bi	1	1656	bi	1	1082	bi	1	1006	bi	1	1087
1	1177	629	bi	1	1657	bi	1	1083	bi	1	1007	bi	1	1088
1	1178	158	bi	1	1658	bi	1	1084	bi	1	1008	bi	1	1089
1	1179	400	bi	1	1659	bi	1	1085	bi	1	1009	bi	1	1090
1	1180	100	bi	1	1660	bi	1	1086	bi	1	1010	bi	1	1091
1	1181	102	bi	1	1661	bi	1	1087	bi	1	1011	bi	1	1092
1	1182	799	bi	1	1662	bi	1	1088	bi	1	1012	bi	1	1093
1	1183	117	bi	1	1663	bi	1	1089	bi	1	1013	bi	1	1094
1	1184	158	bi	1	1664	bi	1	1090	bi	1	1014	bi	1	1095
1	1185	161	bi	1	1665	bi	1	1091	bi	1	1015	bi	1	1096
1	1186	61	bi	1	1666	bi	1	1092	bi	1	1016	bi	1	1097
1	1187	257	bi	1	1667	bi	1	1093	bi	1	1017	bi	1	1098
1	1188	281	bi	1	1668	bi	1	1094	bi	1	1018	bi	1	1099
1	1189	31	-			-			-			-		
1	1190	124	bi	1	1669	bi	1	1095	bi	1	1019	bi	1	1100
1	1191	136	bi	1	1670	bi	1	1096	bi	1	1020	bi	1	1101
1	1192	131	bi	1	1671	bi	1	1097	bi	1	1021	bi	1	1102
1	1193	171	bi	1	1672	bi	1	1098	bi	1	1022	bi	1	1103
1	1194	120	bi	1	1673	bi	1	1099	bi	1	1023	bi	1	1104
1	1195	123	bi	1	1674	bi	1	1100	bi	1	1024	bi	1	1105
1	1196	978	bi	1	1675	bi	1	1101	bi	1	1025	bi	1	1106
1	1197	309	bi	1	1676	bi	1	1102	bi	1	1026	bi	1	1107
1	1198	486	bi	1	1677	bi	1	1103	bi	1	1027	bi	1	1108
1	1199	590	bi	1	1678	bi	1	1104	bi	1	1028	bi	1	1109
1	1200	132	bi	1	1679	bi	1	1105	bi	1	1029	bi	1	1110
1	1201	366	bi	1	1680	bi	1	1106	bi	1	1030	bi	1	1111
1	3303	307	bi	1	1681	bi	1	1107	bi	1	1031	bi	1	1112

1	1202	319	bi	1	1682	bi	1	1108	bi	1	1032	bi	1	1113
1	1203	199	bi	1	1683	bi	1	1109	bi	1	1033	bi	1	1114
1	1204	114	bi	1	1684	bi	1	1110	bi	1	1034	bi	1	1115
1	1205	114	bi	1	1685	bi	1	1111	bi	1	1035	bi	1	1116
1	1206	87	bi	1	1686	bi	1	1112	bi	1	1036	bi	1	1117
1	1207	392	bi	1	1687	bi	1	1113	bi	1	1037	bi	1	1118
1	1208	383	bi	1	1688	bi	1	1114	bi	1	1038	bi	1	1119
1	1209	296	bi	1	1689	bi	1	1115	bi	1	1039	bi	1	1120
1	1210	292	bi	1	1690	bi	1	1116	bi	1	1040	bi	1	1121
1	1211	887	bi	1	1691	bi	1	1118	bi	1	1041	bi	1	1122
1	1212	395	bi	1	1692	bi	1	1119	bi	1	1042	bi	1	1123
1	1213	348	bi	1	1693	bi	1	1120	bi	1	1043	bi	1	1124
1	1214	180	bi	1	1694	bi	1	1121	bi	1	1044	bi	1	1125
1	1215	610	bi	1	1695	bi	1	1122	bi	1	1045	bi	1	1126
1	1216	41	bi	1	1696	bi	1	1123	bi	1	1046	bi	1	1127
1	1217	390	bi	1	1697	bi	1	1124	bi	1	1047	bi	1	1128
1	1218	245	bi	1	1698	bi	1	1125	bi	1	1048	bi	1	1129
1	1219	731	bi	1	1699	bi	1	1126	bi	1	1049	bi	1	1130
1	1220	154	bi	1	1700	bi	1	1127	bi	1	1050	bi	1	1131
1	1221	406	bi	1	1701	bi	1	1128	bi	1	1051	bi	1	1132
1	1222	59	bi	1	1702	bi	1	1129	bi	1	1052	bi	1	1133
1	1223	276	bi	1	1703	bi	1	1130	bi	1	1053	bi	1	1134
1	1224	383	bi	1	1704	bi	1	1131	bi	1	1054	bi	1	1135
1	1225	129	bi	1	1705	bi	1	1132	bi	1	1055	bi	1	1136
1	1226	220	bi	1	1706	bi	1	1133	bi	1	1056	bi	1	1137
1	1227	234	bi	1	1707	bi	1	1134	bi	1	1057	bi	1	1138
1	1228	879	bi	1	1708	bi	1	1135	bi	1	1058	bi	1	1139
1	1229	175	bi	1	1709	bi	1	1136	bi	1	1059	bi	1	1140
1	3304	159	uni	1	1710	bi	1	1137	uni	1	1060	uni	1	1141
1	3305	316	bi	1	1710	uni	1	1137	bi	1	1060	bi	1	1141
1	1230	78	bi	1	1711	bi	1	1138	bi	1	1061	bi	1	1142
1	1231	60	bi	1	1712	bi	1	1139	bi	1	1062	bi	1	1143
1	1232	203	bi	1	1713	bi	1	1140	bi	1	1063	bi	1	1144
1	1233	1416	bi	1	1714	bi	1	1141	bi	1	1064	bi	1	1145
1	1234	236	bi	1	1715	bi	1	1142	bi	1	1065	bi	1	1146
1	1235	378	-			-			-			bi	1	1148
1	1236	267	-			-			-			bi	1	1149
1	1237	315	uni	1	723	uni	1	352	uni	1	297	bi	1	1150
1	1238	198	-			-			-			bi	1	1152
1	1239	238	bi	1	2061	bi	1	1486	bi	1	1411	bi	1	1153
1	1240	244	-			-			-			bi	1	1154
1	1241	437	-			-			-			bi	1	1155
1	1242	235	uni	1	2451	uni	1	1562	uni	1	1374	bi	1	1156
1	1243	144	-			-			-			bi	1	1157
1	1244	305	bi	1	1733	bi	1	1166	bi	1	1093	bi	1	1158
1	1245	154	bi	1	1734	bi	1	1167	bi	1	1094	bi	1	1159
1	1246	167	bi	1	1735	bi	1	1168	bi	1	1095	bi	1	1160
1	1247	780	bi	1	1736	bi	1	1169	bi	1	1096	bi	1	1161
1	1248	163	bi	1	1737	bi	1	1170	bi	1	1097	bi	1	1162
1	1249	288	bi	1	1738	bi	1	1171	bi	1	1098	bi	1	1163
1	1250	448	bi	1	1739	bi	1	1172	bi	1	1099	bi	1	1164
1	1251	417	bi	1	1740	bi	1	1173	bi	1	1100	bi	1	1165
1	1252	38	-			-			-			bi	1	1166
1	1253	589	bi	1	1741	bi	1	1174	bi	1	1101	bi	1	1167
1	1254	542	bi	1	1742	bi	1	1175	bi	1	1102	bi	1	1168
1	1255	538	bi	1	1743	bi	1	1176	bi	1	1103	bi	1	1169
1	1256	194	bi	1	1744	bi	1	1177	bi	1	1104	bi	1	1170

1	1257	31	-			-			-			-		
1	1258	871	bi	1	1745	bi	1	1178	bi	1	1105	bi	1	1171
1	1259	372	bi	1	1746	bi	1	1179	bi	1	1106	bi	1	1172
1	1260	326	bi	1	1747	bi	1	1180	bi	1	1107	bi	1	1173
1	1261	540	bi	1	1748	bi	1	1181	bi	1	1108	bi	1	1174
1	1262	469	bi	1	1749	bi	1	1182	bi	1	1109	bi	1	1175
1	1263	329	bi	1	1750	bi	1	1183	bi	1	1110	bi	1	1177
1	1264	380	bi	1	1751	bi	1	1184	bi	1	1111	bi	1	1178
1	1265	133	bi	1	1752	bi	1	1185	bi	1	1112	bi	1	1179
1	1266	328	bi	1	1753	bi	1	1186	bi	1	1113	bi	1	1180
1	1267	201	bi	1	1754	bi	1	1187	bi	1	1114	bi	1	1181
1	1268	256	bi	1	1755	bi	1	1188	bi	1	1115	bi	1	1182
1	1269	384	bi	1	1756	bi	1	1189	bi	1	1116	bi	1	1183
1	1270	804	bi	1	1758	bi	1	1191	bi	1	1117	bi	1	1184
1	1271	351	bi	1	1759	bi	1	1192	bi	1	1118	bi	1	1185
1	1272	360	bi	1	1760	bi	1	1193	bi	1	1119	bi	1	1186
1	1273	67	bi	1	1761	bi	1	1194	bi	1	1120	bi	1	1187
1	1274	374	bi	1	1762	bi	1	1195	bi	1	1121	bi	1	1188
1	1275	117	bi	1	1764	bi	1	1197	bi	1	1122	bi	1	1193
1	1276	478	bi	1	1765	bi	1	1198	bi	1	1123	bi	1	1194
1	1277	225	bi	1	1766	bi	1	1199	bi	1	1124	bi	1	1195
1	1278	440	bi	1	1767	bi	1	1200	bi	1	1125	bi	1	1196
1	1279	318	bi	1	1768	bi	1	1201	bi	1	1126	bi	1	1197
1	1280	113	bi	1	1769	bi	1	1202	bi	1	1127	bi	1	1198
1	1281	104	bi	1	1770	bi	1	1203	bi	1	1128	bi	1	1199
1	1282	394	bi	1	1771	bi	1	1204	bi	1	1129	bi	1	1200
1	1283	450	bi	1	1772	bi	1	1205	bi	1	1130	bi	1	1201
1	1284	228	bi	1	1773	bi	1	1206	bi	1	1131	bi	1	1202
1	1285	881	bi	1	1774	bi	1	1207	bi	1	1132	bi	1	1203
1	1286	239	bi	1	1775	bi	1	1208	bi	1	1133	bi	1	1204
1	1287	373	bi	1	1776	bi	1	1209	bi	1	1134	bi	1	1205
1	1288	410	bi	1	1777	bi	1	1210	bi	1	1135	bi	1	1206
1	1289	89	bi	1	1778	bi	1	1211	bi	1	1136	bi	1	1207
1	1290	343	bi	1	1779	bi	1	1212	bi	1	1137	bi	1	1208
1	1291	182	bi	1	1780	bi	1	1213	bi	1	1138	bi	1	1209
1	1292	275	bi	1	1781	bi	1	1214	bi	1	1139	bi	1	1210
1	1293	194	bi	1	1782	bi	1	1215	bi	1	1140	bi	1	1211
1	1294	163	bi	1	1783	bi	1	1216	bi	1	1141	bi	1	1212
1	1295	418	bi	1	1784	bi	1	1217	bi	1	1142	bi	1	1214
1	1296	907	bi	1	1785	bi	1	1218	bi	1	1143	bi	1	1216
1	1297	391	bi	1	1786	bi	1	1219	bi	1	1144	bi	1	1217
1	1298	170	bi	1	1787	bi	1	1220	bi	1	1145	bi	1	1218
1	1299	325	bi	1	1788	bi	1	1221	bi	1	1146	bi	1	1219
1	1300	145	bi	1	1789	bi	1	1222	bi	1	1147	bi	1	1220
1	1301	161	bi	1	1790	bi	1	1223	bi	1	1148	bi	1	1221
1	1302	324	bi	1	1791	bi	1	1224	bi	1	1149	bi	1	1222
1	1303	267	bi	1	1792	bi	1	1225	bi	1	1150	bi	1	1223
1	1304	224	bi	1	1793	bi	1	1226	bi	1	1151	bi	1	1224
1	1305	335	bi	1	1794	bi	1	1227	bi	1	1152	bi	1	1225
1	1306	701	bi	1	1795	bi	1	1228	bi	1	1153	bi	1	1226
1	1307	31	-			-			-			-		
1	1308	148	bi	1	1796	bi	1	1229	bi	1	1154	bi	1	1227
1	1309	183	bi	1	1797	bi	1	1230	bi	1	1155	bi	1	1228
1	1310	789	bi	1	1798	bi	1	1231	bi	1	1156	bi	1	1229
1	1311	105	bi	1	1799	bi	1	1232	bi	1	1157	bi	1	1230
1	1312	599	bi	1	1800	bi	1	1233	bi	1	1158	bi	1	1231
1	1313	86	bi	1	1801	bi	1	1234	bi	1	1159	bi	1	1232

1	1314	300	bi	1	1802	bi	1	1235	bi	1	1160	bi	1	1233
1	1315	421	bi	1	1803	bi	1	1236	bi	1	1161	bi	1	1234
1	1316	333	bi	1	1804	bi	1	1237	bi	1	1162	bi	1	1235
1	1317	465	bi	1	1805	bi	1	1238	bi	1	1163	bi	1	1236
1	1318	145	bi	1	1806	bi	1	1239	bi	1	1164	bi	1	1237
1	1319	483	bi	1	1807	bi	1	1240	bi	1	1165	bi	1	1238
1	1320	388	bi	1	1808	bi	1	1241	bi	1	1166	bi	1	1239
1	1321	449	bi	1	1809	bi	1	1242	bi	1	1167	bi	1	1240
1	1322	450	bi	1	1810	bi	1	1243	bi	1	1168	bi	1	1241
1	1323	409	uni	1	2550	uni	1	1710	uni	1	1321	uni	1	1546
1	1324	94	-			-			-			-		
1	1325	198	-			-			-			-		
1	1326	251	uni	1	2886	uni	1	2431	uni	1	2102	uni	1	2259
1	1327	155	uni	1	1664	bi	1	2316	uni	1	1014	uni	1	1860
1	1328	108	uni	1	2343	uni	1	248	uni	1	1122	uni	1	2024
1	1329	62	-			-			-			-		
1	1330	49	-			-			-			-		
1	1331	187	-			-			-			-		
1	1332	77	-			-			-			-		
1	1333	56	-			-			-			-		
1	1334	72	bi	1	1321	bi	1	1701	-			-		
1	1335	97	-			-			-			-		
1	1336	708	-			-			-			-		
1	1337	85	-			-			-			-		
1	1338	396	-			-			-			-		
1	1339	234	uni	1	1587	uni	1	1014	uni	1	940	uni	1	1022
1	1340	255	uni	1	1549	uni	1	976	uni	1	902	uni	1	982
1	1341	167	uni	1	1543	uni	1	971	uni	1	897	uni	1	976
1	1342	105	-			-			-			-		
1	1343	250	uni	3	2938	bi	1	1690	-			-		
1	1344	76	-			-			-			bi	1	1525
1	1345	76	-			-			-			-		
1	3306	142	-			uni	1	2467	-			bi	1	1520
1	1346	146	-			bi	1	2291	-			uni	1	1520
1	1347	113	-			bi	1	2290	-			bi	1	1519
1	1348	118	-			-			-			-		
1	1349	196	bi	1	1338	bi	1	2287	-			bi	1	1518
1	1350	162	-			-			-			bi	1	1841
1	1351	106	-			-			-			-		
1	1352	69	-			-			-			bi	1	1517
1	1353	60	-			-			-			-		
1	1354	37	-			-			-			-		
1	1355	60	-			-			-			-		
1	1356	195	-			-			-			-		
1	1357	90	-			-			-			-		
1	1358	423	bi	1	1341	uni	3	3006	-			bi	1	1835
1	1359	77	bi	1	1343	-			-			bi	1	1833
1	1360	242	-			-			-			-		
1	1361	479	-			-			-			-		
1	1362	465	-			-			-			-		
1	1363	415	-			-			-			-		
1	1364	55	-			-			-			-		
1	1365	96	-			-			-			-		
1	1366	70	-			-			-			-		
1	1367	367	-			-			-			-		
1	1368	157	-			-			-			-		
1	1369	294	-			-			-			-		

1	1370	129	-			-			-			-		
1	1371	114	-			-			-			-		
1	1372	202	-			-			-			-		
1	1373	120	-			-			-			-		
1	1374	159	-			-			-			-		
1	1375	346	-			-			-			-		
1	1376	133	-			-			-			-		
1	1377	111	-			-			-			-		
1	1378	67	-			-			-			-		
1	1379	1722	-			-			-			-		
1	1380	186	-			-			-			-		
1	1381	121	-			-			-			-		
1	1382	277	-			-			-			-		
1	1383	111	-			-			-			-		
1	1384	116	-			-			-			-		
1	1385	385	-			-			-			-		
1	1386	210	-			-			-			-		
1	1387	548	uni	1	1678	uni	1	1104	uni	1	1028	uni	1	1109
1	1388	145	uni	1	1364	uni	1	1660	-			-		
1	1389	43	bi	1	1365	uni	1	1659	-			-		
1	1390	119	-			-			-			-		
1	1391	66	-			-			-			-		
1	1392	369	bi	1	1367	bi	1	2261	uni	1	1030	uni	1	1111
1	1393	135	-			-			-			-		
1	1394	388	-			-			-			-		
1	1395	83	-			bi	1	2257	-			-		
1	1396	60	bi	1	1811	bi	1	1244	bi	1	1169	bi	1	1242
1	1397	148	bi	1	1812	bi	1	1245	bi	1	1170	bi	1	1243
1	1398	108	bi	1	1813	bi	1	1246	bi	1	1171	bi	1	1244
1	1399	660	bi	1	1814	bi	1	1247	bi	1	1172	bi	1	1245
1	1400	158	bi	1	1815	bi	1	1248	bi	1	1173	bi	1	1246
1	1401	194	bi	1	1816	bi	1	1249	bi	1	1174	bi	1	1247
1	1402	334	bi	1	1817	bi	1	1250	bi	1	1175	bi	1	1248
1	1403	104	bi	1	1818	bi	1	1251	bi	1	1176	bi	1	1249
1	1404	596	bi	1	1819	bi	1	1252	bi	1	1177	bi	1	1250
1	1405	459	bi	1	1820	bi	1	1253	bi	1	1178	bi	1	1251
1	1406	212	bi	1	1821	bi	1	1254	bi	1	1179	bi	1	1252
1	1407	107	bi	1	1822	bi	1	1255	bi	1	1180	bi	1	1253
1	1408	314	bi	1	1825	bi	1	1258	bi	1	1183	bi	1	1256
1	1409	627	bi	1	1826	bi	1	1259	bi	1	1184	bi	1	1257
1	1410	431	bi	1	1827	bi	1	1260	bi	1	1185	bi	1	1258
1	1411	333	bi	1	1828	bi	1	1261	bi	1	1186	bi	1	1259
1	1412	178	bi	1	1829	bi	1	1262	bi	1	1187	bi	1	1260
1	1413	128	uni	1	1830	uni	1	1263	uni	1	1188	bi	1	1261
1	1414	161	bi	1	1830	bi	1	1263	bi	1	1188	uni	1	1261
1	1415	180	bi	1	1831	bi	1	1264	bi	1	1189	bi	1	1262
1	1416	115	bi	1	1832	bi	1	1265	bi	1	1190	bi	1	1263
1	1417	355	bi	1	1833	bi	1	1266	bi	1	1191	bi	1	1264
1	1418	459	bi	1	1834	bi	1	1267	bi	1	1192	bi	1	1265
1	1419	551	bi	1	1835	bi	1	1268	bi	1	1193	bi	1	1266
1	1420	278	bi	1	1836	bi	1	1269	bi	1	1194	bi	1	1267
1	1421	666	bi	1	1837	bi	1	1270	bi	1	1195	bi	1	1268
1	1422	49	bi	1	1844	bi	1	1277	bi	1	1202	bi	1	1275
1	1423	210	bi	1	1845	bi	1	1278	bi	1	1203	bi	1	1276
1	1424	779	bi	1	1846	bi	1	1279	bi	1	1204	bi	1	1277
1	1425	629	bi	1	1847	bi	1	1280	bi	1	1205	bi	1	1278
1	1426	369	bi	1	1848	bi	1	1281	bi	1	1206	bi	1	1279

1	1427	692	bi	1	1849	bi	1	1282	bi	1	1207	bi	1	1280
1	1428	294	bi	1	1850	bi	1	1283	bi	1	1208	bi	1	1281
1	1429	150	bi	1	1851	bi	1	1284	bi	1	1209	bi	1	1282
1	1430	337	bi	1	1852	bi	1	1285	bi	1	1210	bi	1	1283
1	1431	439	bi	1	1853	bi	1	1286	bi	1	1211	bi	1	1284
1	1432	101	bi	1	1854	bi	1	1287	bi	1	1212	bi	1	1285
1	1433	433	bi	1	1855	bi	1	1288	bi	1	1213	bi	1	1286
1	1434	180	bi	1	1856	bi	1	1289	bi	1	1214	bi	1	1287
1	1435	48	-			-			-			-		
1	1436	126	bi	1	1857	bi	1	1290	bi	1	1215	bi	1	1290
1	1437	250	bi	1	1858	bi	1	1291	bi	1	1216	bi	1	1291
1	1438	364	bi	1	1859	bi	1	1292	bi	1	1217	bi	1	1292
1	1439	310	bi	1	1860	bi	1	1293	bi	1	1218	bi	1	1293
1	1440	297	bi	1	1861	bi	1	1294	bi	1	1219	bi	1	1294
1	1441	265	bi	1	1862	bi	1	1295	bi	1	1220	bi	1	1295
1	1442	202	bi	1	1863	bi	1	1296	bi	1	1221	bi	1	1296
1	3307	239	bi	1	1864	bi	1	1297	bi	1	1222	bi	1	1297
1	1443	202	bi	1	1865	bi	1	1298	bi	1	1223	bi	1	1298
1	1444	469	bi	1	1866	bi	1	1299	bi	1	1224	bi	1	1299
1	1445	77	bi	1	1867	-			bi	1	1225	bi	1	1300
1	1446	345	bi	1	1868	bi	1	1300	bi	1	1226	bi	1	1301
1	1447	481	bi	1	1869	bi	1	1301	bi	1	1227	bi	1	1302
1	1448	209	bi	1	1870	bi	1	1302	bi	1	1228	bi	1	1303
1	1449	227	bi	1	1871	bi	1	1303	bi	1	1229	bi	1	1304
1	1450	404	bi	1	1872	bi	1	1304	bi	1	1230	bi	1	1305
1	1451	437	bi	1	1873	bi	1	1305	bi	1	1231	bi	1	1306
1	1452	92	bi	1	1874	bi	1	1306	bi	1	1232	bi	1	1307
1	1453	37	-			-			-			-		
1	1454	347	bi	1	1875	bi	1	1307	bi	1	1233	bi	1	1308
1	1455	398	bi	1	1876	bi	1	1308	bi	1	1234	bi	1	1309
1	1456	180	bi	1	1877	bi	1	1309	bi	1	1235	bi	1	1310
1	1457	295	bi	1	1878	bi	1	1310	bi	1	1236	bi	1	1311
1	1458	117	bi	1	1879	bi	1	1311	bi	1	1237	bi	1	1312
1	1459	260	bi	1	1880	bi	1	1312	bi	1	1238	bi	1	1313
1	1460	407	bi	1	1881	bi	1	1313	bi	1	1239	bi	1	1314
1	1461	125	bi	1	1883	bi	1	1315	bi	1	1240	bi	1	1315
1	1462	152	bi	1	1884	bi	1	1316	bi	1	1241	bi	1	1316
1	1463	289	bi	1	1885	bi	1	1317	bi	1	1242	bi	1	1317
1	1464	342	bi	1	1886	bi	1	1318	bi	1	1243	bi	1	1318
1	1465	359	bi	1	1887	bi	1	1319	bi	1	1244	bi	1	1319
1	1466	389	bi	1	1888	bi	1	1320	bi	1	1245	bi	1	1320
1	1467	365	bi	1	1889	bi	1	1321	bi	1	1246	bi	1	1321
1	1468	429	bi	1	1890	bi	1	1322	bi	1	1247	bi	1	1322
1	1469	169	bi	1	1891	bi	1	1323	bi	1	1248	bi	1	1323
1	1470	283	bi	1	1892	bi	1	1324	bi	1	1249	bi	1	1324
1	1471	391	bi	1	1893	bi	1	1325	bi	1	1250	bi	1	1325
1	1472	283	bi	1	1894	bi	1	1326	bi	1	1251	bi	1	1326
1	1473	65	bi	1	1895	bi	1	1327	bi	1	1252	bi	1	1327
1	1474	159	bi	1	1896	bi	1	1328	bi	1	1253	bi	1	1328
1	1475	111	bi	1	1897	bi	1	1329	bi	1	1254	bi	1	1329
1	1476	685	bi	1	1898	bi	1	1330	bi	1	1255	bi	1	1330
1	1477	632	bi	1	1899	bi	1	1331	bi	1	1256	bi	1	1331
1	1478	316	bi	1	1900	bi	1	1332	bi	1	1257	bi	1	1332
1	1479	165	bi	1	1901	bi	1	1333	bi	1	1258	bi	1	1333
1	1480	390	bi	1	1902	bi	1	1334	bi	1	1259	bi	1	1334
1	1481	210	bi	1	1903	bi	1	1335	bi	1	1260	bi	1	1335
1	1482	81	bi	1	1904	bi	1	1336	bi	1	1261	bi	1	1336

1	3308	592	bi	1	1905	bi	1	1337	bi	1	1262	bi	1	1337
1	3309	64	-			-			-			-		
1	1483	397	bi	1	1906	bi	1	1338	bi	1	1263	bi	1	1338
1	1484	287	bi	1	1907	bi	1	1339	bi	1	1264	bi	1	1339
1	1485	311	bi	1	1908	bi	1	1340	bi	1	1265	bi	1	1340
1	1486	148	bi	1	1909	bi	1	1341	bi	1	1266	bi	1	1341
1	1487	447	bi	1	1910	bi	1	1342	bi	1	1267	bi	1	1342
1	1488	191	bi	1	1911	bi	1	1343	bi	1	1268	bi	1	1343
1	1489	167	-			-			bi	1	1269	bi	1	1344
1	1490	176	bi	1	1912	bi	1	1344	bi	1	1270	bi	1	1345
1	1491	273	bi	1	1913	bi	1	1345	bi	1	1271	bi	1	1346
1	1492	583	bi	1	1914	bi	1	1346	bi	1	1272	bi	1	1347
1	1493	593	bi	1	1915	bi	1	1347	bi	1	1273	bi	1	1348
1	1494	35	-			-			-			-		
1	1495	142	bi	1	1916	bi	1	1348	bi	1	1274	bi	1	1349
1	1496	337	bi	1	1917	bi	1	1349	bi	1	1275	bi	1	1350
1	1497	479	bi	1	1918	bi	1	1350	bi	1	1276	bi	1	1352
1	1498	478	bi	1	1919	bi	1	1351	bi	1	1277	bi	1	1353
1	1499	282	bi	1	1920	bi	1	1352	bi	1	1278	bi	1	1354
1	1500	643	bi	1	1921	bi	1	1353	bi	1	1279	bi	1	1355
1	1501	558	bi	1	1922	bi	1	1354	bi	1	1280	bi	1	1356
1	1502	487	bi	1	1923	bi	1	1355	bi	1	1281	bi	1	1357
1	1503	327	bi	1	1924	bi	1	1356	bi	1	1282	bi	1	1358
1	1504	54	bi	1	1925	bi	1	1357	-			bi	1	1359
1	1505	470	bi	1	1926	bi	1	1358	bi	1	1283	bi	1	1360
1	3310	200	uni	1	1927	uni	1	1359	uni	1	1284	uni	1	1361
1	3311	443	bi	1	1927	bi	1	1359	bi	1	1284	bi	1	1361
1	1506	487	bi	1	1935	bi	1	1360	bi	1	1285	bi	1	1368
1	1507	184	bi	1	1936	bi	1	1361	bi	1	1286	bi	1	1369
1	1508	295	bi	1	1937	bi	1	1362	bi	1	1287	bi	1	1370
1	1509	309	bi	1	1938	bi	1	1363	bi	1	1288	bi	1	1371
1	1510	255	bi	1	1939	bi	1	1364	bi	1	1289	bi	1	1372
1	1511	749	bi	1	1940	bi	1	1365	bi	1	1290	bi	1	1373
1	1512	821	bi	1	1941	bi	1	1366	bi	1	1291	bi	1	1374
1	1513	686	bi	1	1942	bi	1	1367	bi	1	1292	bi	1	1375
1	1514	143	bi	1	1943	bi	1	1368	bi	1	1293	bi	1	1376
1	1515	148	bi	1	1944	bi	1	1369	bi	1	1294	bi	1	1377
1	1516	367	bi	1	1945	bi	1	1370	bi	1	1295	bi	1	1378
1	1517	88	bi	1	1946	bi	1	1371	bi	1	1296	bi	1	1379
1	1518	200	bi	1	1947	bi	1	1372	bi	1	1297	bi	1	1380
1	1519	212	bi	1	1948	bi	1	1373	bi	1	1298	bi	1	1381
1	1520	181	bi	1	1949	bi	1	1374	bi	1	1299	bi	1	1382
1	1521	97	bi	1	1950	bi	1	1375	bi	1	1300	bi	1	1383
1	1522	493	bi	1	1951	bi	1	1376	bi	1	1301	bi	1	1384
1	1523	173	bi	1	1952	bi	1	1377	bi	1	1302	bi	1	1385
1	1524	218	bi	1	1953	bi	1	1378	bi	1	1303	bi	1	1386
1	1525	304	bi	1	1954	bi	1	1379	bi	1	1304	bi	1	1387
1	1526	455	bi	1	1955	bi	1	1380	bi	1	1305	bi	1	1388
1	3312	475	bi	1	1956	bi	1	1381	bi	1	1306	bi	1	1389
1	1527	478	bi	1	1957	bi	1	1382	bi	1	1307	bi	1	1390
1	1528	191	bi	1	1958	bi	1	1383	bi	1	1308	bi	1	1391
1	1529	116	bi	1	1959	bi	1	1384	bi	1	1309	bi	1	1392
1	1530	377	bi	1	1960	bi	1	1385	bi	1	1310	bi	1	1393
1	1531	194	bi	1	1961	bi	1	1386	bi	1	1311	bi	1	1394
1	1532	148	bi	1	1962	bi	1	1387	bi	1	1312	bi	1	1395
1	1533	256	bi	1	1963	bi	1	1388	bi	1	1313	bi	1	1396
1	1534	336	bi	1	1964	bi	1	1389	bi	1	1314	bi	1	1397

1	1535	320	bi	1	1965	bi	1	1390	bi	1	1315	bi	1	1398
1	1536	218	bi	1	1966	bi	1	1391	bi	1	1316	bi	1	1399
1	1537	292	bi	1	1967	bi	1	1392	bi	1	1317	bi	1	1400
1	1538	261	bi	1	1968	bi	1	1393	bi	1	1318	bi	1	1401
1	1539	468	bi	1	1969	bi	1	1394	bi	1	1319	bi	1	1402
1	1540	183	bi	1	1970	bi	1	1395	bi	1	1320	bi	1	1403
1	1541	300	bi	1	1971	bi	1	1396	bi	1	1321	bi	1	1404
1	1542	436	bi	1	1972	bi	1	1397	bi	1	1322	bi	1	1405
1	1543	693	bi	1	1973	bi	1	1398	bi	1	1323	bi	1	1406
1	1544	218	bi	1	1974	bi	1	1399	bi	1	1324	bi	1	1407
1	1545	288	bi	1	1975	bi	1	1400	bi	1	1325	bi	1	1408
1	1546	256	bi	1	1976	bi	1	1401	bi	1	1326	bi	1	1409
1	1547	284	bi	1	1977	bi	1	1402	bi	1	1327	bi	1	1410
1	1548	314	bi	1	1978	bi	1	1403	bi	1	1328	bi	1	1411
1	1549	297	bi	1	1979	bi	1	1404	bi	1	1329	bi	1	1412
1	1550	351	bi	1	1980	bi	1	1405	bi	1	1330	bi	1	1413
1	1551	433	bi	1	1981	bi	1	1406	bi	1	1331	bi	1	1414
1	1552	329	bi	1	1982	bi	1	1407	bi	1	1332	bi	1	1415
1	1553	331	bi	1	1983	bi	1	1408	bi	1	1333	bi	1	1416
1	1554	470	bi	1	1984	bi	1	1409	bi	1	1334	bi	1	1417
1	1555	361	bi	1	1985	bi	1	1410	bi	1	1335	bi	1	1418
1	1556	274	bi	1	1986	bi	1	1411	bi	1	1336	bi	1	1419
1	1557	345	bi	1	1987	bi	1	1412	bi	1	1337	bi	1	1420
1	1558	241	bi	1	1988	bi	1	1413	bi	1	1338	bi	1	1421
1	1559	233	bi	1	1989	bi	1	1414	bi	1	1339	bi	1	1422
1	1560	265	bi	1	1990	bi	1	1415	bi	1	1340	bi	1	1423
1	1561	143	bi	1	1991	bi	1	1416	bi	1	1341	bi	1	1424
1	1562	321	bi	1	1992	bi	1	1417	bi	1	1342	bi	1	1425
1	1563	201	bi	1	1993	bi	1	1418	bi	1	1343	bi	1	1426
1	1564	314	bi	1	1994	bi	1	1419	bi	1	1344	bi	1	1427
1	1565	428	bi	1	1995	bi	1	1420	bi	1	1345	bi	1	1428
1	1566	227	bi	1	1996	bi	1	1421	bi	1	1346	bi	1	1429
1	1567	244	bi	1	1997	bi	1	1422	bi	1	1347	bi	1	1431
1	1568	285	bi	1	1998	bi	1	1423	bi	1	1348	bi	1	1432
1	1569	123	bi	1	1999	bi	1	1424	bi	1	1349	bi	1	1433
1	1570	148	bi	1	2000	bi	1	1425	bi	1	1350	bi	1	1434
1	1571	185	bi	1	2001	bi	1	1426	bi	1	1351	bi	1	1435
1	1572	478	bi	1	2002	bi	1	1427	bi	1	1352	bi	1	1436
1	1573	73	bi	1	2003	bi	1	1428	bi	1	1353	bi	1	1437
1	1574	172	bi	1	2004	bi	1	1429	bi	1	1354	bi	1	1438
1	1575	215	bi	1	2005	bi	1	1430	bi	1	1355	bi	1	1439
1	1576	276	bi	1	2006	bi	1	1431	bi	1	1356	bi	1	1440
1	1577	281	bi	1	2007	bi	1	1432	bi	1	1357	bi	1	1441
1	1578	221	bi	1	2008	bi	1	1433	bi	1	1358	bi	1	1442
1	1579	66	bi	1	2009	bi	1	1434	bi	1	1359	bi	1	1443
1	1580	171	bi	1	2010	bi	1	1435	bi	1	1360	bi	1	1444
1	1581	771	bi	1	2011	bi	1	1436	bi	1	1361	bi	1	1445
1	1582	156	bi	1	2012	bi	1	1437	bi	1	1362	bi	1	1446
1	1583	263	bi	1	2013	bi	1	1438	bi	1	1363	bi	1	1447
1	1584	314	bi	1	2014	bi	1	1439	bi	1	1364	bi	1	1448
1	1585	189	bi	1	2015	bi	1	1440	bi	1	1365	bi	1	1449
1	1586	79	bi	1	2016	bi	1	1441	bi	1	1366	bi	1	1450
1	1587	92	bi	1	2017	bi	1	1442	bi	1	1367	bi	1	1451
1	1588	242	bi	1	2018	bi	1	1443	bi	1	1368	bi	1	1452
1	1589	161	bi	1	2019	bi	1	1444	bi	1	1369	bi	1	1453
1	1590	251	bi	1	2020	bi	1	1445	bi	1	1370	bi	1	1454
1	1591	473	bi	1	2021	bi	1	1446	bi	1	1371	bi	1	1455

1	1592	113	bi	1	2022	bi	1	1447	bi	1	1372	bi	1	1456
1	1593	155	bi	1	2023	bi	1	1448	bi	1	1373	bi	1	1457
1	1594	237	bi	1	2024	bi	1	1449	bi	1	1374	bi	1	1458
1	1595	592	bi	1	2025	bi	1	1450	bi	1	1375	bi	1	1459
1	1596	297	bi	1	2026	bi	1	1451	bi	1	1376	bi	1	1460
1	1597	393	bi	1	2027	bi	1	1452	bi	1	1377	bi	1	1461
1	1598	897	bi	1	2028	bi	1	1453	bi	1	1378	bi	1	1462
1	1599	430	bi	1	2029	bi	1	1454	bi	1	1379	bi	1	1463
1	1600	349	bi	1	2030	bi	1	1455	bi	1	1380	bi	1	1464
1	1601	310	bi	1	2031	bi	1	1456	bi	1	1381	bi	1	1465
1	1602	233	bi	1	2032	bi	1	1457	bi	1	1382	bi	1	1466
1	1603	211	bi	1	2033	bi	1	1458	bi	1	1383	bi	1	1467
1	1604	238	bi	1	2034	bi	1	1459	bi	1	1384	bi	1	1468
1	1605	313	bi	1	2035	bi	1	1460	bi	1	1385	bi	1	1469
1	1606	264	bi	1	2036	bi	1	1461	bi	1	1386	bi	1	1470
1	1607	1061	bi	1	2037	bi	1	1462	bi	1	1387	bi	1	1471
1	1608	360	bi	1	2038	bi	1	1463	bi	1	1388	bi	1	1472
1	1609	428	bi	1	2039	bi	1	1464	bi	1	1389	bi	1	1473
1	1610	309	bi	1	2040	bi	1	1465	bi	1	1390	bi	1	1474
1	1611	427	bi	1	2041	bi	1	1466	bi	1	1391	bi	1	1475
1	1612	179	bi	1	2042	bi	1	1467	bi	1	1392	bi	1	1476
1	3313	302	bi	1	2043	bi	1	1468	bi	1	1393	bi	1	1477
1	1613	162	bi	1	2044	bi	1	1469	bi	1	1394	bi	1	1478
1	1614	235	bi	1	2045	bi	1	1470	bi	1	1395	bi	1	1479
1	1615	557	bi	1	2046	bi	1	1471	bi	1	1396	bi	1	1480
1	1616	69	bi	1	2047	bi	1	1472	bi	1	1397	bi	1	1481
1	1617	154	bi	1	2048	bi	1	1473	bi	1	1398	bi	1	1482
1	1618	136	bi	1	2049	bi	1	1474	bi	1	1399	bi	1	1483
1	1619	165	bi	1	2050	bi	1	1475	bi	1	1400	bi	1	1484
1	1620	254	bi	1	2051	bi	1	1476	bi	1	1401	bi	1	1485
1	1621	595	bi	1	2052	bi	1	1477	bi	1	1402	bi	1	1486
1	1622	584	bi	1	2053	bi	1	1478	bi	1	1403	bi	1	1487
1	1623	73	bi	1	2054	bi	1	1479	bi	1	1404	bi	1	1488
1	1624	32	-			-			-			-		
1	1625	298	bi	1	2055	bi	1	1480	bi	1	1405	bi	1	1489
1	1626	156	bi	1	2056	bi	1	1481	bi	1	1406	bi	1	1490
1	1627	221	bi	1	2057	bi	1	1482	bi	1	1407	bi	1	1491
1	1628	421	bi	1	2058	bi	1	1483	bi	1	1408	bi	1	1547
1	1629	804	bi	1	2059	bi	1	1484	bi	1	1409	bi	1	1548
1	1630	334	bi	1	2062	bi	1	1487	bi	1	1412	bi	1	1552
1	1631	368	bi	1	2063	bi	1	1488	bi	1	1413	bi	1	1553
1	1632	196	bi	1	2064	bi	1	1489	bi	1	1414	bi	1	1554
1	1633	144	bi	1	2065	bi	1	1490	bi	1	1415	bi	1	1555
1	1634	299	bi	1	2066	bi	1	1491	bi	1	1416	bi	1	1556
1	1635	341	bi	1	2067	bi	1	1492	bi	1	1417	bi	1	1557
1	1636	279	bi	1	2068	bi	1	1493	bi	1	1418	bi	1	1558
1	1637	312	bi	1	2069	bi	1	1494	bi	1	1419	bi	1	1559
1	1638	321	bi	1	2070	bi	1	1495	bi	1	1420	bi	1	1560
1	1639	119	bi	1	2071	bi	1	1496	bi	1	1421	bi	1	1561
1	1640	108	bi	1	2072	bi	1	1497	bi	1	1422	bi	1	1562
1	1641	534	bi	1	2073	bi	1	1498	bi	1	1423	bi	1	1563
1	1642	226	bi	1	2074	bi	1	1499	bi	1	1424	bi	1	1564
1	1643	253	bi	1	2075	bi	1	1500	bi	1	1425	bi	1	1565
1	1644	270	bi	1	2076	bi	1	1501	bi	1	1426	bi	1	1566
1	1645	295	bi	1	2077	bi	1	1502	bi	1	1427	bi	1	1567
1	1646	307	bi	1	2078	bi	1	1503	bi	1	1428	bi	1	1568
1	1647	285	bi	1	2079	bi	1	1504	bi	1	1429	bi	1	1569

1	1648	295	bi	1	2080	bi	1	1505	bi	1	1430	bi	1	1570
1	1649	229	bi	1	2081	bi	1	1506	bi	1	1431	bi	1	1571
1	3362	367	uni	1	2082	uni	1	1507	uni	1	1432	uni	1	1572
1	3363	25	bi	1	2083	-			-			bi	1	1573
1	1650	846	bi	1	2084	bi	1	1508	bi	1	1433	bi	1	1574
1	1651	184	bi	1	2085	bi	1	1509	bi	1	1434	bi	1	1575
1	1652	229	bi	1	2086	bi	1	1510	bi	1	1435	bi	1	1576
1	1653	439	bi	1	2087	bi	1	1511	bi	1	1436	bi	1	1577
1	1654	239	uni	1	542	bi	1	1512	bi	1	1437	bi	1	1578
1	1655	112	bi	1	2088	bi	1	1513	bi	1	1438	bi	1	1579
1	1656	96	bi	1	2089	bi	1	1514	bi	1	1439	bi	1	1580
1	1657	221	bi	1	2090	bi	1	1515	bi	1	1440	bi	1	1581
1	1658	78	bi	1	2091	bi	1	1516	bi	1	1441	bi	1	1582
1	1659	255	bi	1	2092	bi	1	1517	bi	1	1442	bi	1	1583
1	1660	174	bi	1	2093	bi	1	1518	bi	1	1443	bi	1	1584
1	1661	245	bi	1	2095	bi	1	1519	bi	1	1444	bi	1	1585
1	1662	110	bi	1	2096	bi	1	1521	-			bi	1	1586
1	1663	416	bi	1	2097	bi	1	1522	bi	1	1445	bi	1	1587
1	1664	514	bi	1	2098	bi	1	1523	bi	1	1446	bi	1	1588
1	1665	191	bi	1	2099	bi	1	1524	bi	1	1447	bi	1	1589
1	1666	344	bi	1	2100	bi	1	1525	bi	1	1448	bi	1	1590
1	1667	480	bi	1	2101	bi	1	1526	bi	1	1449	bi	1	1591
1	1668	740	bi	1	2102	bi	1	1527	bi	1	1450	bi	1	1592
1	1669	225	bi	1	2103	bi	1	1528	bi	1	1451	bi	1	1593
1	1670	84	bi	1	2104	bi	1	1529	bi	1	1452	bi	1	1594
1	1671	238	bi	1	2105	bi	1	1530	bi	1	1453	bi	1	1595
1	1672	375	bi	1	2106	bi	1	1531	bi	1	1454	bi	1	1596
1	1673	163	bi	1	2107	bi	1	1532	bi	1	1455	bi	1	1597
1	1674	289	bi	1	2108	bi	1	1533	bi	1	1456	bi	1	1599
1	1675	292	bi	1	2109	bi	1	1534	bi	1	1457	bi	1	1600
1	1676	547	bi	1	2110	bi	1	1535	bi	1	1458	bi	1	1601
1	1677	81	bi	1	2111	bi	1	1536	bi	1	1459	bi	1	1602
1	1678	340	bi	1	2112	bi	1	1537	bi	1	1460	bi	1	1603
1	1679	151	bi	1	2113	bi	1	1538	bi	1	1461	bi	1	1604
1	1680	204	bi	1	2114	bi	1	1539	bi	1	1462	bi	1	1605
1	1681	215	bi	1	2115	bi	1	1540	bi	1	1463	bi	1	1606
1	1682	385	bi	1	2116	bi	1	1541	bi	1	1464	bi	1	1607
1	1683	1325	bi	1	2117	bi	1	1542	bi	1	1465	bi	1	1608
1	1684	136	bi	1	2118	bi	1	1543	bi	1	1466	bi	1	1609
1	1685	270	bi	1	2119	bi	1	1544	bi	1	1467	bi	1	1610
1	1686	300	bi	1	2120	bi	1	1545	bi	1	1468	bi	1	1611
1	1687	160	bi	1	2121	bi	1	1546	bi	1	1469	bi	1	1612
1	1688	595	bi	1	2122	bi	1	1547	bi	1	1470	bi	1	1613
1	1689	314	bi	1	2123	bi	1	1548	bi	1	1471	bi	1	1614
1	1690	332	bi	1	2124	bi	1	1549	bi	1	1472	bi	1	1615
1	1691	391	bi	1	2125	bi	1	1550	bi	1	1473	bi	1	1616
1	1692	244	bi	1	2126	bi	1	1551	bi	1	1474	bi	1	1617
1	1693	301	bi	1	2127	bi	1	1552	bi	1	1475	bi	1	1618
1	1694	282	bi	1	2128	bi	1	1553	bi	1	1476	bi	1	1619
1	1695	80	bi	1	2129	-			-			-		
1	1696	687	bi	1	2130	bi	1	1554	bi	1	1477	uni	1	1084
1	1697	470	bi	1	2131	bi	1	1555	bi	1	1478	uni	1	1669
1	1698	285	bi	1	2132	bi	1	1556	bi	1	1479	uni	1	1412
1	1699	277	bi	1	2133	bi	1	1557	bi	1	1480	-		
1	1700	285	bi	1	2134	bi	1	1558	bi	1	1481	-		
1	1701	511	bi	1	2135	bi	1	1559	bi	1	1482	-		
1	1702	448	bi	1	2136	bi	1	1560	bi	1	1483	bi	1	2541

1	1703	243	-			bi	1	1561	bi	1	1484	-		
1	1704	244	bi	1	181	bi	1	1562	bi	1	1485	uni	1	1156
1	1705	653	uni	1	2730	bi	1	1563	bi	1	1486	uni	1	2100
1	1706	1867	uni	1	892	uni	1	2637	bi	1	1487	uni	1	2467
1	1707	129	-			-			-			-		
1	1708	339	uni	1	1994	uni	1	1419	bi	1	1488	uni	1	1427
1	1709	327	-			-			bi	1	1502	-		
1	1710	237	uni	1	2237	uni	1	1599	bi	1	1503	uni	1	1653
1	1711	281	uni	1	894	uni	1	2642	bi	1	1504	uni	1	2477
1	1712	284	uni	1	120	uni	1	1621	bi	1	1505	uni	1	2478
1	3315	166	uni	1	279	uni	1	2813	bi	1	1506	uni	1	2633
1	3316	144	uni	1	2118	bi	1	1564	bi	1	1507	bi	1	1620
1	1713	96	uni	1	496	uni	1	116	bi	1	1508	uni	1	114
1	1714	172	bi	1	834	bi	1	431	bi	1	1509	bi	1	447
1	1715	143	bi	1	833	bi	1	430	bi	1	1510	bi	1	446
1	1716	165	bi	1	905	-			bi	1	1511	-		
1	1717	101	-			-			bi	1	1512	-		
1	1718	468	bi	1	907	-			bi	1	1513	-		
1	1719	251	bi	1	831	bi	1	428	bi	1	1514	bi	1	444
1	3317	282	uni	1	1432	uni	1	855	bi	1	1515	uni	1	863
1	1720	195	bi	1	2137	uni	1	2504	bi	1	1516	uni	1	2279
1	1721	302	bi	1	2139	-			bi	1	1517	-		
1	1722	139	bi	1	2140	-			-			-		
1	1723	34	-			-			-			-		
1	1724	393	bi	1	2141	uni	1	2294	uni	1	1321	bi	1	1864
1	1725	104	bi	1	2142	-			-			-		
1	1726	82	bi	1	528	uni	1	414	bi	1	794	-		
1	1727	134	bi	1	2143	uni	1	415	bi	1	793	uni	1	360
1	1728	612	bi	1	2144	uni	1	631	uni	1	530	uni	1	1613
1	3318	421	bi	1	2145	uni	1	834	uni	1	726	uni	1	840
1	1729	75	-			-			-			-		
1	1730	431	bi	1	2146	-			-			uni	1	424
1	3319	110	bi	1	2164	bi	1	1928	-			bi	1	363
1	3320	279	bi	1	2165	bi	1	421	-			bi	1	437
1	1731	132	bi	1	2168	-			-			-		
1	1732	283	bi	1	2169	-			-			-		
1	1733	276	bi	1	2170	-			-			-		
1	1734	282	bi	1	2171	-			-			-		
1	3321	49	bi	1	2172	-			-			uni	1	424
1	3322	318	bi	1	2173	-			-			uni	1	424
1	1735	440	bi	1	2174	uni	1	1450	uni	1	1375	uni	1	1459
1	1736	218	bi	1	2175	uni	1	2877	uni	1	2474	uni	1	2690
1	3323	130	bi	1	2176	-			bi	1	1667	-		
1	3324	207	bi	1	2177	bi	1	2031	-			bi	1	420
1	1737	459	bi	1	2178	uni	1	1937	-			-		
1	1738	219	bi	1	2179	bi	1	1936	uni	1	2215	uni	1	2421
1	1739	426	bi	1	2180	bi	1	1937	uni	1	298	uni	1	299
1	1740	162	bi	1	2181	-			-			-		
1	1741	137	bi	1	2182	-			-			-		
1	1742	397	bi	1	1025	-			-			bi	1	424
1	1743	304	bi	1	2185	-			bi	1	783	-		
1	1744	335	bi	1	2186	uni	1	1962	uni	1	782	uni	1	1276
1	1745	708	bi	1	2187	-			uni	1	781	-		
1	1746	816	bi	1	2188	-			bi	1	780	-		
1	1747	130	bi	1	2189	-			bi	1	778	-		
1	1748	243	bi	1	2190	-			-			-		
1	1749	276	bi	1	2191	-			-			-		

1	1750	168	bi	1	2192	-			bi	1	774	-		
1	1751	74	bi	1	2193	-			bi	1	773	-		
1	1752	417	bi	1	2194	-			-			-		
1	1753	45	bi	1	2196	-			-			-		
1	1754	395	bi	1	2197	-			bi	1	772	-		
1	1755	56	bi	1	2198	-			-			-		
1	1756	44	bi	1	2199	-			-			-		
1	1757	147	bi	1	2201	-			-			bi	1	364
1	1758	119	bi	1	2202	-			bi	1	769	-		
1	3325	269	bi	1	2203	-			-			-		
1	3326	405	bi	1	2204	-			-			-		
1	1759	339	bi	1	2205	-			uni	1	769	-		
1	1760	118	bi	1	2206	-			-			-		
1	1761	125	bi	1	2207	-			bi	1	768	-		
1	1762	105	bi	1	2208	-			uni	1	767	-		
1	1763	1076	bi	1	2209	uni	1	1985	bi	1	765	bi	1	1623
1	1764	221	bi	1	2210	-			-			bi	1	1625
1	1765	116	bi	1	2211	bi	1	1573	bi	1	1518	bi	1	1626
1	1766	247	bi	1	2212	bi	1	1574	bi	1	1519	bi	1	1627
1	1767	175	bi	1	2213	bi	1	1575	bi	1	1520	bi	1	1628
1	1768	454	bi	1	2214	bi	1	1576	bi	1	1521	bi	1	1629
1	1769	125	bi	1	2215	bi	1	1577	bi	1	1522	bi	1	1630
1	1770	203	bi	1	2216	bi	1	1578	bi	1	1523	bi	1	1631
1	1771	601	bi	1	2217	bi	1	1579	bi	1	1524	bi	1	1632
1	1772	42	-			-			bi	1	2167	-		
1	1773	229	bi	1	2218	bi	1	1580	bi	1	1525	bi	1	1633
1	1774	162	bi	1	2219	bi	1	1581	bi	1	1526	bi	1	1634
1	1775	446	bi	1	2220	bi	1	1582	bi	1	1527	bi	1	1636
1	1776	315	bi	1	2221	bi	1	1583	bi	1	1528	bi	1	1637
1	1777	320	bi	1	2222	bi	1	1584	bi	1	1529	bi	1	1638
1	1778	204	bi	1	2223	bi	1	1585	bi	1	1530	bi	1	1639
1	1779	294	bi	1	2224	bi	1	1586	bi	1	1531	bi	1	1640
1	1780	101	bi	1	2225	bi	1	1587	bi	1	1532	bi	1	1641
1	1781	33	-			-			-			-		
1	1782	51	-			bi	1	1588	bi	1	1533	bi	1	1642
1	1783	194	bi	1	2226	bi	1	1589	bi	1	1534	bi	1	1643
1	1784	418	bi	1	2227	bi	1	1590	bi	1	1535	bi	1	1644
1	1785	343	bi	1	2228	bi	1	1591	bi	1	1536	bi	1	1645
1	1786	163	bi	1	2229	bi	1	1592	bi	1	1537	bi	1	1646
1	1787	458	bi	1	2230	bi	1	1593	bi	1	1538	bi	1	1647
1	1788	307	bi	1	2231	bi	1	1594	bi	1	1539	bi	1	1648
1	1789	637	bi	1	2232	bi	1	1595	bi	1	1540	bi	1	1649
1	1790	481	bi	1	2233	bi	1	1596	bi	1	1541	bi	1	1650
1	1791	282	bi	1	2235	bi	1	1597	bi	1	1542	bi	1	1651
1	1792	180	bi	1	2236	bi	1	1598	bi	1	1543	bi	1	1652
1	1793	235	bi	1	2237	bi	1	1599	bi	1	1544	bi	1	1653
1	1794	610	bi	1	2238	bi	1	1600	bi	1	1545	bi	1	1654
1	1795	502	bi	1	2239	bi	1	1601	bi	1	1546	bi	1	1655
1	1796	489	bi	1	2241	bi	1	1602	bi	1	1547	bi	1	1656
1	1797	445	bi	1	2242	bi	1	1603	bi	1	1548	bi	1	1657
1	1798	119	bi	1	2243	bi	1	1604	bi	1	1549	bi	1	1660
1	1799	54	bi	1	2244	bi	1	1605	bi	1	1550	bi	1	1661
1	1800	50	bi	1	2245	bi	1	1606	-			bi	1	1662
1	1801	145	bi	1	2246	bi	1	1607	bi	1	1554	bi	1	1663
1	1802	179	bi	1	2247	bi	1	1608	bi	1	1555	bi	1	1664
1	1803	902	bi	1	2249	bi	1	1609	bi	1	1556	bi	1	1665
1	1804	217	bi	1	2250	bi	1	1610	bi	1	1557	bi	1	1666

1	1805	222	bi	1	2251	bi	1	1611	bi	1	1558	bi	1	1667
1	1806	132	bi	1	2252	bi	1	1612	bi	1	1559	bi	1	1668
1	1807	394	bi	1	2253	bi	1	1613	bi	1	1560	bi	1	1669
1	1808	48	-			-			bi	1	105	-		
1	1809	612	bi	1	2254	bi	1	1614	bi	1	1561	bi	1	1670
1	1810	34	-			-			-			-		
1	1811	50	bi	1	2255	bi	1	1615	bi	1	1562	bi	1	1671
1	1812	117	bi	1	2256	bi	1	1616	bi	1	1563	bi	1	1672
1	1813	117	bi	1	2257	bi	1	1617	bi	1	1564	bi	1	1673
1	1814	335	bi	1	2258	bi	1	1618	bi	1	1565	bi	1	1674
1	1815	359	bi	1	2259	bi	1	1619	bi	1	1566	bi	1	1675
1	1816	277	bi	1	2260	bi	1	1620	bi	1	1567	bi	1	1676
1	1817	264	bi	1	2261	bi	1	1621	bi	1	1568	bi	1	1677
1	3327	157	bi	1	2262	bi	1	1622	bi	1	1569	bi	1	1679
1	3328	140	bi	1	2263	bi	1	1623	bi	1	1570	bi	1	1680
1	1818	924	bi	1	2264	bi	1	1624	bi	1	1571	bi	1	1681
1	1819	72	-			-			-			-		
1	1820	454	bi	1	2265	bi	1	1625	-			bi	1	1682
1	1821	275	bi	1	2266	bi	1	1626	-			bi	1	1683
1	1822	433	bi	1	2267	bi	1	1627	bi	1	1576	bi	1	1684
1	1823	252	bi	1	2268	bi	1	1628	bi	1	1577	bi	1	1685
1	1824	398	bi	1	2269	bi	1	1629	bi	1	1578	bi	1	1686
1	1825	334	bi	1	2270	bi	1	1630	bi	1	1579	bi	1	1687
1	1826	346	bi	1	2271	bi	1	1631	bi	1	1580	bi	1	1688
1	1827	293	bi	1	2272	bi	1	1632	bi	1	1581	bi	1	1689
1	1828	190	bi	1	2273	bi	1	1633	bi	1	1582	bi	1	1690
1	1829	173	bi	1	2274	bi	1	1634	bi	1	1583	bi	1	1691
1	1830	272	bi	1	2275	bi	1	1635	bi	1	1584	bi	1	1692
1	1831	590	bi	1	2276	bi	1	1636	bi	1	1585	bi	1	1693
1	1832	434	bi	1	2277	bi	1	1637	bi	1	1586	bi	1	1694
1	1833	83	-			-			-			-		
1	1834	149	bi	1	2278	bi	1	1638	bi	1	1587	bi	1	1695
1	1835	738	bi	1	2279	bi	1	1639	bi	1	1588	bi	1	1696
1	1836	251	bi	1	2280	bi	1	1640	bi	1	1589	bi	1	1697
1	1837	316	bi	1	2281	bi	1	1641	bi	1	1590	bi	1	1698
1	1838	162	bi	1	2282	bi	1	1642	bi	1	1591	bi	1	1699
1	1839	230	bi	1	2283	bi	1	1643	bi	1	1592	bi	1	1700
1	1840	426	bi	1	2284	bi	1	1644	bi	1	1593	bi	1	1701
1	1841	111	bi	1	2285	bi	1	1645	bi	1	1594	bi	1	1702
1	1842	98	-			-			uni	1	672	-		
1	1843	158	bi	1	2286	bi	1	1646	bi	1	1595	bi	1	1704
1	1844	396	bi	1	2287	bi	1	1647	bi	1	1596	bi	1	1705
1	3329	65	uni	1	2288	bi	1	1648	uni	1	1597	uni	1	1706
1	3330	262	bi	1	2288	uni	1	1648	bi	1	1597	bi	1	1706
1	1845	118	bi	1	2289	bi	1	1649	bi	1	1598	bi	1	1707
1	1846	145	bi	1	2290	bi	1	1650	bi	1	1599	bi	1	1708
1	1847	156	bi	1	2291	bi	1	1651	-			-		
1	1848	42	bi	1	2292	bi	1	1652	-			-		
1	1849	314	bi	1	2293	bi	1	1653	-			-		
1	1850	74	bi	1	2294	bi	1	1654	-			-		
1	1851	67	bi	1	2295	bi	1	1656	uni	1	501	uni	1	622
1	1852	414	bi	1	2296	bi	1	1657	uni	1	518	uni	1	2335
1	1853	78	bi	1	2297	-			-			-		
1	3331	74	bi	1	2298	-			-			-		
1	1854	52	bi	1	2299	bi	1	2437	-			bi	1	2338
1	1855	107	bi	1	2300	bi	1	2438	-			bi	1	2339
1	3332	164	bi	1	2301	bi	1	2439	-			bi	1	2340

1	3333	96	bi	1	2302	bi	1	1662	-			uni	1	2341
1	3334	199	bi	1	2303	bi	1	1663	-			uni	1	2342
1	3335	226	bi	1	2304	bi	1	1664	-			uni	1	2343
1	1856	931	bi	1	2305	bi	1	1665	-			uni	1	2344
1	1857	245	bi	1	2306	bi	1	1666	-			bi	1	1497
1	1858	975	bi	1	2307	bi	1	1667	uni	1	1025	uni	1	1813
1	1859	117	bi	1	2308	bi	1	1668	-			-		
1	1860	283	bi	1	2309	bi	1	1669	-			-		
1	1861	125	bi	1	2310	bi	1	1670	-			-		
1	1862	133	bi	1	2311	bi	1	1671	-			-		
1	1863	100	bi	1	2312	bi	1	1672	-			-		
1	3336	115	bi	1	2313	bi	1	1673	-			-		
1	1864	61	bi	1	2314	bi	1	1674	-			-		
1	1865	296	bi	1	2315	bi	1	1675	-			-		
1	1866	208	bi	1	2316	bi	1	1676	-			-		
1	1867	107	bi	1	2317	bi	1	1677	-			-		
1	1868	77	bi	1	2318	-			-			-		
1	1869	635	bi	1	2319	bi	1	1679	-			-		
1	1870	491	bi	1	2320	bi	1	1680	-			-		
1	1871	464	-			-			-			-		
1	1872	154	-			uni	1	271	-			-		
1	1873	65	-			-			-			-		
1	1874	271	-			-			-			-		
1	1875	56	bi	1	1340	-			-			-		
1	1876	308	-			-			-			-		
1	1877	97	-			-			-			-		
1	1878	139	bi	1	1339	bi	1	1684	-			bi	1	1516
1	1879	87	-			-			-			-		
1	3337	86	bi	1	2328	bi	1	1691	-			-		
1	1880	286	bi	1	1330	bi	1	1694	-			bi	1	1848
1	1881	67	-			-			-			-		
1	3338	128	bi	1	1329	-			-			bi	1	1849
1	1882	245	bi	1	1327	-			-			bi	1	1531
1	1883	106	-			-			-			-		
1	1884	38	bi	1	1325	-			-			bi	1	1534
1	1885	113	bi	1	1324	bi	1	1699	-			uni	1	2374
1	1886	63	bi	1	1320	bi	1	1702	-			-		
1	1887	241	bi	1	1319	bi	1	1703	-			bi	1	1537
1	1888	104	bi	1	2341	bi	1	1704	-			-		
1	1889	64	bi	1	2342	bi	1	1705	-			-		
1	1890	114	bi	1	2343	bi	1	1706	uni	1	1122	uni	1	2378
1	1891	217	bi	1	2344	bi	1	1707	-			-		
1	1892	205	-			-			-			-		
1	1893	383	bi	1	2347	bi	1	1710	uni	1	1219	uni	1	1546
1	3339	168	bi	1	2348	bi	1	1711	bi	1	1600	bi	1	1709
1	1894	92	bi	1	2349	bi	1	1712	bi	1	1601	bi	1	1710
1	1895	349	bi	1	2350	bi	1	1713	bi	1	1602	bi	1	1711
1	1896	323	bi	1	2351	bi	1	1714	bi	1	1603	bi	1	1712
1	1897	500	bi	1	2352	bi	1	1715	bi	1	1604	bi	1	1713
1	1898	358	bi	1	2353	bi	1	1716	bi	1	1605	bi	1	1714
1	1899	610	bi	1	2354	bi	1	1717	bi	1	1606	bi	1	1715
1	1900	250	bi	1	2355	bi	1	1718	bi	1	1607	bi	1	1716
1	1901	234	bi	1	2356	bi	1	1719	bi	1	1608	bi	1	1717
1	1902	808	bi	1	2357	bi	1	1720	bi	1	1609	bi	1	1718
1	1903	404	bi	1	2358	bi	1	1722	bi	1	1611	bi	1	1719
1	1904	302	bi	1	2359	bi	1	1723	bi	1	1612	bi	1	1720
1	1905	327	bi	1	2360	bi	1	1724	bi	1	1613	bi	1	1721

1	1906	589	bi	1	2361	bi	1	1725	bi	1	1614	bi	1	1722
1	1907	578	bi	1	2362	bi	1	1726	bi	1	1615	bi	1	1723
1	1908	338	bi	1	2363	bi	1	1727	bi	1	1616	bi	1	1724
1	1909	473	bi	1	2364	bi	1	1728	bi	1	1617	bi	1	1725
1	1910	33	-			-			-			-		
1	1911	288	bi	1	2365	bi	1	1730	bi	1	1619	bi	1	1726
1	1912	694	bi	1	2366	bi	1	1731	bi	1	1620	bi	1	1727
1	1913	122	bi	1	2367	bi	1	1732	bi	1	1621	bi	1	1728
1	1914	190	bi	1	2368	bi	1	1733	bi	1	1622	bi	1	1729
1	1915	172	bi	1	2369	bi	1	1734	bi	1	1623	bi	1	1730
1	1916	413	bi	1	2370	bi	1	1735	bi	1	1624	bi	1	1731
1	1917	50	-			-			-			-		
1	1918	375	bi	1	2372	bi	1	1736	bi	1	1625	bi	1	1732
1	1919	115	bi	1	2373	bi	1	1737	bi	1	1626	bi	1	1733
1	1920	382	bi	1	2374	bi	1	1738	bi	1	1627	bi	1	1734
1	1921	325	bi	1	2375	bi	1	1739	bi	1	1628	bi	1	1735
1	1922	250	bi	1	2376	bi	1	1740	bi	1	1629	bi	1	1736
1	1923	290	bi	1	2377	bi	1	1741	bi	1	1630	bi	1	1737
1	1924	309	bi	1	2378	bi	1	1742	bi	1	1631	bi	1	1738
1	1925	513	bi	1	2379	bi	1	1743	bi	1	1632	bi	1	1739
1	1926	36	bi	1	2380	bi	1	1744	bi	1	1633	bi	1	1740
1	1927	398	bi	1	2381	bi	1	1745	bi	1	1634	bi	1	1741
1	1928	278	bi	1	2382	bi	1	1746	bi	1	1635	bi	1	1742
1	1929	227	bi	1	2383	bi	1	1747	bi	1	1636	bi	1	1743
1	1930	357	bi	1	2384	bi	1	1748	bi	1	1637	bi	1	1744
1	1931	69	-			-			-			-		
1	1932	83	-			uni	1	2257	-			-		
1	1933	323	-			-			-			-		
1	1934	329	uni	1	2412	uni	1	1657	-			bi	1	1492
1	1935	93	-			-			-			-		
1	1936	85	-			-			-			-		
1	1937	140	-			-			-			-		
1	1938	286	-			-			-			-		
1	1939	163	-			-			-			-		
1	1940	230	-			-			-			-		
1	1941	859	uni	1	2305	uni	1	2443	-			uni	1	2344
1	1942	301	-			-			-			-		
1	1943	372	-			-			-			-		
1	1944	1721	uni	1	2307	uni	1	1667	-			bi	1	1813
1	1945	102	-			-			-			-		
1	1946	187	-			-			-			-		
1	1947	445	-			-			-			-		
1	1948	147	-			-			-			-		
1	1949	130	-			-			-			-		
1	1950	72	-			-			-			-		
1	1951	236	-			-			-			-		
1	1952	393	-			-			-			-		
1	1953	344	-			-			-			-		
1	1954	103	-			-			-			-		
1	1955	60	-			-			-			-		
1	1956	110	-			-			-			-		
1	1957	194	-			-			-			-		
1	1958	177	-			-			-			-		
1	1959	427	-			-			-			-		
1	1960	520	-			-			-			-		
1	1961	140	-			-			-			-		
1	1962	401	uni	1	1341	bi	3	3006	-			uni	1	1835

1	1963	142	uni	1	1339	uni	1	1684	-			uni	1	1837
1	1964	42	-			-			-			-		
1	1965	67	bi	3	2940	-			-			-		
1	1966	185	uni	4	3018	bi	1	1686	-			uni	1	1842
1	1967	106	-			-			-			-		
1	1968	181	-			bi	1	2465	-			-		
1	1969	75	-			bi	3	2949	-			-		
1	1970	40	-			bi	3	2948	-			-		
1	1971	71	-			bi	3	2947	-			-		
1	1972	161	-			-			-			bi	1	1845
1	1973	57	-			-			-			-		
1	1974	117	-			-			-			-		
1	1975	111	-			-			-			bi	1	1526
1	1976	142	uni	1	1333	uni	1	1691	-			-		
1	1977	290	uni	1	1668	uni	1	2472	uni	1	1018	uni	1	2367
1	1978	284	uni	1	2331	uni	1	1694	-			-		
1	1979	271	-			uni	1	2474	-			uni	1	2370
1	1980	308	-			uni	1	2475	-			uni	1	2371
1	1981	111	-			-			-			-		
1	1982	97	-			-			-			-		
1	1983	109	-			-			-			uni	1	2374
1	1984	70	uni	1	1321	bi	1	254	-			-		
1	1985	37	-			bi	1	2309	-			-		
1	1986	84	-			-			-			-		
1	1987	176	-			-			-			-		
1	1988	101	-			-			-			-		
1	1989	71	-			-			-			-		
1	1990	120	uni	1	2343	uni	1	1706	-			-		
1	1991	156	uni	1	1664	uni	1	1090	uni	1	1014	uni	1	1095
1	1992	287	-			-			-			-		
1	1993	381	uni	1	2347	uni	1	1710	uni	1	807	uni	1	2447
1	1994	497	bi	1	2385	bi	1	1749	bi	1	1638	bi	1	1746
1	1995	64	bi	1	2386	bi	1	1750	bi	1	1639	bi	1	1747
1	1996	322	bi	1	2387	bi	1	1751	bi	1	1640	bi	1	1748
1	1997	97	bi	1	2388	bi	1	1752	bi	1	1641	bi	1	1749
1	1998	427	bi	1	2389	bi	1	1753	bi	1	1642	bi	1	1750
1	1999	603	bi	1	2390	bi	1	1754	bi	1	1643	bi	1	1751
1	2000	278	bi	1	2391	bi	1	1755	bi	1	1644	bi	1	1752
1	2001	569	bi	1	2392	bi	1	1756	bi	1	1645	bi	1	1753
1	2002	184	bi	1	2393	bi	1	1757	bi	1	1646	bi	1	1754
1	3340	131	bi	1	2394	bi	1	1758	bi	1	1647	bi	1	1755
1	2003	388	bi	1	2395	bi	1	1759	bi	1	1648	bi	1	1756
1	2004	295	bi	1	2396	bi	1	1760	bi	1	1649	bi	1	1757
1	2005	1230	bi	1	2397	bi	1	1761	bi	1	1650	bi	1	1758
1	2006	447	bi	1	2399	bi	1	1762	bi	1	1652	bi	1	1760
1	2007	128	bi	1	2400	bi	1	1763	bi	1	1653	bi	1	1761
1	2008	414	bi	1	2401	bi	1	1764	bi	1	1654	bi	1	1762
1	2009	310	bi	1	2402	bi	1	1765	bi	1	1655	bi	1	1763
1	2010	249	bi	1	2403	bi	1	1766	bi	1	1656	bi	1	1764
1	2011	604	-			-			-			-		
1	2012	325	bi	1	2405	bi	1	1767	bi	1	1658	bi	1	1766
1	2013	474	-			bi	1	1768	-			-		
1	2014	324	uni	1	2429	uni	1	1794	uni	1	1685	uni	1	1790
1	2015	275	uni	1	2413	bi	1	1770	-			uni	1	1774
1	2016	468	bi	1	2406	bi	1	1771	bi	1	1661	bi	1	1767
1	2017	325	bi	1	2409	bi	1	1773	bi	1	1665	bi	1	1770
1	2018	353	bi	1	2410	bi	1	1774	uni	1	1682	bi	1	1771

1	2019	235	bi	1	2411	bi	1	1775	-			bi	1	1772
1	2020	393	uni	1	1025	-			-			uni	1	424
1	2021	894	bi	1	2412	bi	1	1777	bi	1	1668	bi	1	1773
1	2022	283	bi	1	2413	bi	1	1778	bi	1	1669	bi	1	1774
1	2023	253	bi	1	2414	uni	1	1783	bi	1	1670	bi	1	1775
1	2024	466	bi	1	2415	bi	1	1780	bi	1	1671	bi	1	1776
1	2025	434	bi	1	2416	bi	1	1781	bi	1	1672	bi	1	1777
1	2026	657	bi	1	2417	bi	1	1782	bi	1	1673	bi	1	1778
1	2027	714	bi	1	2418	bi	1	1783	bi	1	1674	bi	1	1779
1	2028	1048	bi	1	2419	bi	1	1784	bi	1	1675	bi	1	1780
1	2029	406	bi	1	2420	bi	1	1785	bi	1	1676	bi	1	1781
1	2030	265	bi	1	2421	bi	1	1786	bi	1	1677	bi	1	1782
1	2031	114	bi	1	2422	bi	1	1787	bi	1	1678	bi	1	1783
1	2032	393	uni	1	1025	-			-			uni	1	424
1	2033	84	-			-			-			-		
1	2034	391	uni	1	2146	-			-			uni	1	1007
1	2035	235	-			-			-			-		
1	2036	122	bi	1	2423	bi	1	1788	bi	1	1679	bi	1	1784
1	2037	242	bi	1	2424	bi	1	1789	bi	1	1680	bi	1	1785
1	2038	300	bi	1	2425	bi	1	1790	bi	1	1681	bi	1	1786
1	2039	343	bi	1	2426	bi	1	1791	bi	1	1682	bi	1	1787
1	2040	191	bi	1	2427	bi	1	1792	bi	1	1683	bi	1	1788
1	2041	289	bi	1	2428	bi	1	1793	bi	1	1684	bi	1	1789
1	2042	238	bi	1	2429	bi	1	1794	bi	1	1685	bi	1	1790
1	2043	276	bi	1	2430	bi	1	1795	bi	1	1686	bi	1	1791
1	2044	263	bi	1	2431	bi	1	1796	bi	1	1687	bi	1	1792
1	2045	379	bi	1	2432	bi	1	1797	bi	1	1688	bi	1	1793
1	2046	303	bi	1	2433	bi	1	1798	bi	1	1689	bi	1	1794
1	2047	255	bi	1	2434	bi	1	1799	bi	1	1690	bi	1	1795
1	2048	148	bi	1	2435	bi	1	1800	bi	1	1691	bi	1	1796
1	2049	158	bi	1	2436	bi	1	1801	bi	1	1692	bi	1	1797
1	2050	191	bi	1	2437	bi	1	1802	bi	1	1693	bi	1	1798
1	3341	111	uni	1	2438	uni	1	1803	uni	1	1694	uni	1	1799
1	3342	311	bi	1	2438	bi	1	1803	bi	1	1694	bi	1	1799
1	2051	127	-			-			bi	1	1695	bi	1	1800
1	2052	174	bi	1	2439	bi	1	1804	bi	1	1696	bi	1	1865
1	2053	284	bi	1	2440	bi	1	1805	bi	1	1697	bi	1	1866
1	2054	282	bi	1	2441	bi	1	1806	bi	1	1698	bi	1	1867
1	2055	113	bi	1	2442	bi	1	1807	bi	1	1699	bi	1	1868
1	2056	235	bi	1	2443	bi	1	1808	bi	1	1700	bi	1	1869
1	2057	118	bi	1	2444	bi	1	1809	bi	1	1701	bi	1	1870
1	2058	335	bi	1	2445	bi	1	1810	bi	1	1702	bi	1	1871
1	2059	489	bi	1	2446	bi	1	1811	bi	1	1703	bi	1	1872
1	2060	127	bi	1	2447	bi	1	1812	bi	1	1704	bi	1	1873
1	2061	161	bi	1	2448	bi	1	1813	bi	1	1705	bi	1	1874
1	2062	371	bi	1	2449	bi	1	1814	bi	1	1706	bi	1	1875
1	2063	714	bi	1	2450	bi	1	1815	bi	1	1707	bi	1	1876
1	2064	494	bi	1	2451	bi	1	1816	bi	1	1708	bi	1	1877
1	2065	571	bi	1	2452	bi	1	1817	bi	1	1709	bi	1	1878
1	2066	212	bi	1	2453	bi	1	1818	bi	1	1710	bi	1	1879
1	2067	489	bi	1	2454	bi	1	1819	bi	1	1711	bi	1	1880
1	2068	308	bi	1	2455	bi	1	1820	bi	1	1712	bi	1	1881
1	2069	313	bi	1	2456	bi	1	1821	bi	1	1713	bi	1	1882
1	2070	1500	bi	1	2458	bi	1	1823	bi	1	1714	bi	1	1884
1	2071	256	bi	1	2459	bi	1	1824	bi	1	1715	bi	1	1885
1	2072	576	bi	1	2460	bi	1	1825	bi	1	1716	bi	1	1886
1	2073	593	bi	1	2461	bi	1	1826	bi	1	1717	bi	1	1887

1	2074	335	bi	1	2462	bi	1	1827	bi	1	1718	bi	1	1888
1	2075	1260	bi	1	2464	bi	1	1828	bi	1	1719	-		
1	2076	323	bi	1	2465	bi	1	1829	bi	1	1720	-		
1	2077	224	bi	1	2466	bi	1	1830	bi	1	1721	-		
1	2078	293	bi	1	2467	bi	1	1831	bi	1	1722	uni	1	1163
1	2079	301	bi	1	2468	bi	1	1832	bi	1	1723	uni	1	2312
1	2080	426	bi	1	2469	bi	1	1833	bi	1	1724	uni	1	1165
1	2081	381	bi	1	2470	bi	1	1834	bi	1	1725	-		
1	2082	600	bi	1	2471	bi	1	1835	bi	1	1726	-		
1	2083	129	bi	1	2472	bi	1	1836	bi	1	1727	-		
1	2084	354	bi	1	2473	bi	1	1837	bi	1	1728	uni	1	898
1	2085	278	bi	1	2474	bi	1	1838	bi	1	1729	-		
1	2086	383	uni	1	530	bi	1	1841	-			bi	1	1522
1	2087	62	uni	1	529	-			-			-		
1	2088	73	-			-			-			-		
1	2089	136	-			-			-			-		
1	2090	137	-			-			-			-		
1	2091	245	uni	1	2895	uni	1	2493	uni	1	2111	uni	1	2268
1	2092	1005	bi	1	2487	bi	1	1925	uni	1	2443	uni	1	2406
1	2093	73	-			-			-			-		
1	2094	648	uni	1	2487	uni	1	2835	uni	1	2443	uni	1	2661
1	2095	56	-			-			-			-		
1	2096	110	-			-			-			-		
1	2097	350	bi	1	2488	uni	1	2581	uni	1	2199	uni	1	2405
1	2098	223	uni	1	307	uni	1	2835	uni	1	2443	uni	1	2610
1	2099	370	uni	1	1971	uni	1	1841	uni	1	1321	uni	1	1404
1	2100	434	uni	1	1855	uni	1	1288	uni	1	1213	uni	1	1286
1	2101	206	uni	1	2284	uni	1	1644	uni	1	1593	uni	1	1701
1	2102	231	uni	1	2818	uni	1	2363	uni	1	2037	uni	1	2191
1	2103	186	-			-			-			-		
1	2104	52	-			-			-			-		
1	2105	265	uni	1	2474	uni	1	1838	uni	1	1729	-		
1	2106	268	uni	1	733	uni	1	362	uni	1	307	uni	1	307
1	2107	277	uni	1	732	uni	1	361	uni	1	306	uni	1	306
1	2108	334	uni	1	731	uni	1	360	uni	1	305	uni	1	305
1	2109	216	uni	1	730	uni	1	359	uni	1	304	uni	1	304
1	2110	139	uni	1	965	uni	1	451	uni	1	2265	uni	1	2481
1	2111	643	uni	1	963	-			-			-		
1	2112	272	bi	1	961	uni	1	2653	uni	1	2260	uni	1	2477
1	2113	272	uni	1	2261	uni	1	1621	uni	1	1568	uni	1	2478
1	2114	167	uni	1	122	uni	1	2656	uni	1	2264	uni	1	2480
1	2115	396	uni	1	2470	uni	1	630	uni	1	529	-		
1	2116	246	uni	1	2583	uni	1	2647	uni	1	1805	uni	1	1951
1	2117	74	uni	1	2449	uni	1	1814	uni	1	1706	uni	1	1875
1	2118	584	uni	1	2449	uni	1	1814	uni	1	1706	uni	1	1875
1	2119	303	uni	1	2185	-			uni	1	783	-		
1	2120	341	uni	1	2186	uni	1	1962	bi	1	782	uni	1	1276
1	2121	705	uni	1	2187	-			bi	1	781	-		
1	2122	816	uni	1	2188	-			uni	1	780	-		
1	2123	130	uni	1	2189	-			uni	1	778	-		
1	2124	205	-			-			-			-		
1	2125	538	-			bi	1	2860	-			bi	1	2381
1	2126	59	-			-			-			-		
1	2127	287	-			-			-			-		
1	2128	404	-			-			-			-		
1	2129	187	-			-			-			-		
1	2130	40	-			-			-			-		

1	2131	149	-			-			-			-		
1	2132	127	-			-			uni	1	793	-		
1	2133	122	uni	1	2752	uni	1	2231	uni	1	1972	uni	1	2124
1	2134	115	-			-			-			-		
1	2135	203	-			-			-			-		
1	2136	343	uni	1	1181	uni	1	665	uni	1	558	uni	1	671
1	2137	324	uni	1	210	uni	1	2746	uni	1	2351	uni	1	2569
1	2138	276	-			-			-			-		
1	2139	269	bi	1	2893	bi	1	2491	bi	1	2109	bi	1	2266
1	2140	448	uni	1	2025	uni	1	1450	uni	1	1375	uni	1	1459
1	2141	221	uni	1	1583	uni	1	1010	uni	1	936	uni	1	1018
1	2142	63	-			bi	1	2853	-			-		
1	2143	73	-			-			-			-		
1	2144	471	uni	3	2961	-			-			uni	1	375
1	2145	92	-			-			-			-		
1	2146	317	-			-			-			-		
1	2147	113	uni	3	2960	-			-			uni	1	376
1	2148	3174	-			bi	1	1951	-			-		
1	2149	34	-			-			-			-		
1	2150	73	-			-			-			-		
1	2151	150	-			-			-			-		
1	2152	54	-			-			-			-		
1	2153	706	uni	1	2366	uni	1	1731	uni	1	1620	uni	1	1727
1	2154	76	-			-			-			-		
1	2155	429	-			-			-			-		
1	2156	83	-			-			-			-		
1	2157	283	-			-			-			-		
1	2158	34	-			-			-			-		
1	2159	926	uni	1	522	uni	1	2443	uni	1	782	bi	1	1804
1	2160	372	-			-			-			-		
1	2161	826	uni	3	2951	-			-			uni	1	387
1	2162	140	-			-			-			-		
1	2163	435	-			-			-			-		
1	3343	185	-			-			-			-		
1	2164	290	-			-			-			-		
1	2165	64	-			-			-			-		
1	2166	629	uni	1	2204	-			-			-		
1	2167	44	-			-			-			-		
1	2168	592	uni	3	2957	bi	3	2983	-			uni	1	381
1	2169	160	-			-			-			-		
1	2170	169	-			-			-			-		
1	2171	144	-			-			-			-		
1	2172	286	-			-			-			-		
1	2173	153	-			-			-			-		
1	2174	329	uni	3	2933	uni	1	1997	-			-		
1	2175	167	uni	1	2192	-			uni	1	774	-		
1	2176	74	uni	1	2193	-			uni	1	773	-		
1	2177	46	uni	1	2196	-			-			-		
1	2178	395	uni	1	2197	-			uni	1	772	-		
1	2179	85	-			-			bi	1	771	-		
1	2180	332	-			bi	3	3005	bi	1	770	bi	1	2643
1	2181	274	-			-			-			-		
1	2182	153	-			-			-			-		
1	2183	449	uni	1	2202	uni	1	1720	uni	1	769	uni	1	1718
1	2184	118	uni	1	2206	-			-			-		
1	2185	124	uni	1	2207	-			uni	1	768	-		
1	2186	105	uni	1	2208	-			bi	1	767	-		

1	2187	1075	uni	1	2209	uni	1	2104	uni	1	765	uni	1	1623
1	2188	611	-			bi	1	2923	-			-		
1	2189	460	-			-			-			-		
1	2190	310	bi	1	2481	bi	1	1999	-			-		
1	2191	69	-			-			-			-		
1	2192	612	bi	1	2482	bi	1	2000	bi	1	1740	bi	1	1889
1	2193	407	bi	1	2483	bi	1	2001	bi	1	1741	bi	1	1890
1	2194	160	bi	1	2484	bi	1	2002	bi	1	1742	bi	1	1891
1	2195	869	bi	1	2485	bi	1	2003	bi	1	1743	bi	1	1892
1	2196	124	bi	1	2486	bi	1	2004	bi	1	1744	bi	1	1893
1	3344	186	bi	1	1643	bi	1	1069	-			bi	1	414
1	3345	231	uni	1	1644	uni	1	2322	-			bi	1	413
1	2197	38	bi	1	1644	bi	1	1070	-			-		
1	2198	432	bi	1	2489	bi	1	2005	bi	1	1745	bi	1	1894
1	2199	372	bi	1	2490	bi	1	2006	bi	1	1746	bi	1	1895
1	2200	35	-			-			-			-		
1	2201	435	bi	1	2491	bi	1	2007	bi	1	1747	bi	1	1896
1	2202	194	bi	1	2492	bi	1	2008	bi	1	1748	bi	1	1897
1	2203	229	bi	1	2493	bi	1	2009	bi	1	1749	bi	1	1898
1	2204	214	bi	1	2494	bi	1	2010	bi	1	1750	bi	1	1899
1	2205	73	bi	1	2495	bi	1	2011	bi	1	1751	bi	1	1900
1	2206	173	bi	1	2496	bi	1	2012	bi	1	1752	bi	1	1901
1	2207	320	bi	1	2497	bi	1	2013	bi	1	1753	bi	1	1902
1	2208	451	bi	1	2498	bi	1	2014	bi	1	1754	bi	1	1903
1	2209	400	bi	1	2499	bi	1	2015	bi	1	1755	bi	1	1904
1	2210	173	bi	1	2500	bi	1	2016	bi	1	1756	bi	1	1905
1	2211	930	bi	1	2501	bi	1	2017	bi	1	1757	bi	1	1906
1	2212	438	bi	1	2503	bi	1	2019	bi	1	1758	bi	1	1908
1	2213	486	bi	1	2505	bi	1	2021	bi	1	1759	bi	1	1909
1	2214	1452	bi	1	2506	bi	1	2022	bi	1	1760	bi	1	1910
1	2215	573	bi	1	2514	bi	1	2023	bi	1	1767	bi	1	1917
1	2216	423	bi	1	2515	bi	1	2024	bi	1	1768	bi	1	1918
1	2217	32	-			-			-			-		
1	2218	260	bi	1	2516	bi	1	2025	bi	1	1769	uni	1	307
1	2219	201	bi	1	2517	bi	1	2026	bi	1	1770	-		
1	2220	35	-			-			-			-		
1	2221	291	-			-			-			-		
1	2222	117	-			-			-			-		
1	2223	281	uni	1	350	uni	1	2886	uni	1	2483	uni	1	2700
1	2224	42	-			-			-			-		
1	2225	79	-			-			-			-		
1	2226	465	bi	1	2551	bi	1	2032	bi	1	1773	bi	1	1919
1	2227	156	bi	1	2552	bi	1	2033	bi	1	1774	bi	1	1920
1	2228	412	bi	1	2553	bi	1	2034	bi	1	1775	bi	1	1921
1	2229	429	bi	1	2554	bi	1	2035	bi	1	1776	bi	1	1922
1	2230	257	bi	1	2555	bi	1	2036	bi	1	1777	bi	1	1923
1	2231	186	bi	1	2556	bi	1	2037	bi	1	1778	bi	1	1924
1	2232	241	bi	1	2557	bi	1	2038	bi	1	1779	bi	1	1925
1	2233	294	bi	1	2558	bi	1	2039	bi	1	1780	bi	1	1926
1	2234	262	bi	1	2559	bi	1	2040	bi	1	1781	bi	1	1927
1	2235	249	bi	1	2560	bi	1	2041	bi	1	1782	bi	1	1928
1	2236	259	bi	1	2561	bi	1	2042	bi	1	1783	bi	1	1929
1	2237	92	bi	1	2562	bi	1	2043	bi	1	1784	bi	1	1930
1	2238	48	bi	1	2563	bi	1	2044	bi	1	1785	bi	1	1931
1	2239	57	bi	1	2564	bi	1	2045	bi	1	1786	bi	1	1932
1	2240	695	bi	1	2565	bi	1	2046	bi	1	1787	bi	1	1933
1	2241	303	bi	1	2566	bi	1	2047	bi	1	1788	bi	1	1934

1	2242	34	-			-			-			-		
1	2243	263	bi	1	2567	bi	1	2048	bi	1	1789	bi	1	1935
1	2244	301	bi	1	2568	bi	1	2049	bi	1	1790	bi	1	1936
1	2245	136	bi	1	2569	bi	1	2050	bi	1	1791	bi	1	1937
1	2246	160	bi	1	2570	bi	1	2051	bi	1	1792	bi	1	1938
1	2247	733	bi	1	2571	bi	1	2052	bi	1	1793	bi	1	1939
1	2248	325	bi	1	2572	bi	1	2053	bi	1	1794	bi	1	1940
1	2249	150	bi	1	2573	bi	1	2054	bi	1	1795	bi	1	1941
1	2250	59	bi	1	2574	bi	1	2055	bi	1	1796	bi	1	1942
1	2251	145	bi	1	2575	bi	1	2056	bi	1	1797	bi	1	1943
1	2252	277	bi	1	2576	bi	1	2057	bi	1	1798	bi	1	1944
1	2253	288	bi	1	2577	bi	1	2058	bi	1	1799	bi	1	1945
1	2254	352	bi	1	2578	bi	1	2059	bi	1	1800	bi	1	1946
1	2255	431	bi	1	2579	bi	1	2060	bi	1	1801	bi	1	1947
1	2256	96	bi	1	2580	bi	1	2061	bi	1	1802	bi	1	1948
1	2257	261	bi	1	2581	bi	1	2062	bi	1	1803	bi	1	1949
1	2258	575	bi	1	2582	bi	1	2063	bi	1	1804	bi	1	1950
1	2259	484	bi	1	2583	bi	1	2064	bi	1	1805	bi	1	1951
1	2260	343	bi	1	2584	bi	1	2065	bi	1	1806	bi	1	1952
1	2261	111	bi	1	2585	bi	1	2066	bi	1	1807	bi	1	1953
1	2262	326	bi	1	2586	bi	1	2067	bi	1	1808	bi	1	1954
1	2263	444	bi	1	2587	bi	1	2068	bi	1	1809	bi	1	1955
1	2264	462	bi	1	2588	bi	1	2069	bi	1	1810	bi	1	1956
1	2265	209	bi	1	2589	bi	1	2070	bi	1	1811	bi	1	1957
1	2266	237	bi	1	2590	bi	1	2071	bi	1	1812	bi	1	1958
1	2267	283	bi	1	2591	bi	1	2072	bi	1	1813	bi	1	1959
1	2268	485	bi	1	2592	bi	1	2073	bi	1	1814	bi	1	1960
1	2269	297	bi	1	2593	bi	1	2074	bi	1	1815	bi	1	1961
1	2270	370	bi	1	2594	bi	1	2075	bi	1	1816	bi	1	1962
1	2271	165	bi	1	2595	bi	1	2076	bi	1	1818	bi	1	1963
1	2272	277	bi	1	2596	bi	1	2077	bi	1	1819	bi	1	1964
1	2273	250	bi	1	2597	bi	1	2078	bi	1	1820	bi	1	1965
1	2274	208	bi	1	2598	bi	1	2079	bi	1	1821	bi	1	1966
1	2275	345	bi	1	2599	bi	1	2080	bi	1	1822	bi	1	1967
1	2276	84	bi	1	2600	bi	1	2081	bi	1	1823	bi	1	1968
1	2277	179	bi	1	2601	bi	1	2082	bi	1	1824	bi	1	1969
1	2278	314	bi	1	2602	bi	1	2083	bi	1	1825	bi	1	1970
1	2279	343	bi	1	2603	bi	1	2084	bi	1	1826	bi	1	1971
1	2280	780	bi	1	2604	bi	1	2085	bi	1	1827	bi	1	1972
1	2281	168	bi	1	2605	bi	1	2086	bi	1	1828	bi	1	1973
1	2282	215	bi	1	2606	bi	1	2087	bi	1	1829	bi	1	1974
1	2283	351	bi	1	2607	bi	1	2088	bi	1	1831	bi	1	1975
1	2284	164	bi	1	2608	bi	1	2089	bi	1	1832	bi	1	1976
1	2285	185	bi	1	2609	bi	1	2090	bi	1	1833	bi	1	1977
1	2286	117	bi	1	2610	bi	1	2091	bi	1	1834	bi	1	1978
1	2287	145	bi	1	2611	bi	1	2092	bi	1	1835	bi	1	1979
1	2288	375	bi	1	2612	bi	1	2093	bi	1	1836	bi	1	1980
1	2289	1143	bi	1	2613	bi	1	2094	bi	1	1837	bi	1	1981
1	2290	403	bi	1	2614	bi	1	2095	bi	1	1838	bi	1	1982
1	2291	102	bi	1	2615	bi	1	2096	bi	1	1839	bi	1	1983
1	2292	590	bi	1	2616	bi	1	2097	bi	1	1840	bi	1	1984
1	2293	612	bi	1	2617	bi	1	2098	bi	1	1841	bi	1	1985
1	2294	261	bi	1	2618	bi	1	2099	bi	1	1842	bi	1	1986
1	2295	93	bi	1	2619	bi	1	2100	bi	1	1843	bi	1	1987
1	2296	422	bi	1	2620	bi	1	2101	bi	1	1844	bi	1	1988
1	2297	330	bi	1	2621	bi	1	2102	bi	1	1845	uni	1	712
1	2298	48	bi	1	2622	bi	1	2103	bi	1	1846	-		

1	2299	1512	bi	1	2623	bi	1	2104	bi	1	1847	uni	1	1088
1	2300	93	bi	1	2624	bi	1	2105	bi	1	1848	bi	1	1989
1	2301	108	bi	1	2626	bi	1	2107	bi	1	1849	bi	1	1990
1	2302	172	bi	1	2627	bi	1	2108	bi	1	1850	bi	1	1991
1	2303	564	bi	1	2628	bi	1	2109	bi	1	1851	bi	1	1992
1	2304	349	bi	1	2629	bi	1	2110	bi	1	1852	bi	1	1993
1	2305	155	bi	1	2630	bi	1	2111	bi	1	1853	bi	1	1994
1	2306	156	bi	1	2631	bi	1	2112	bi	1	1854	bi	1	1995
1	2307	252	bi	1	2632	bi	1	2113	bi	1	1855	bi	1	1996
1	2308	681	bi	1	2633	bi	1	2114	bi	1	1856	bi	1	1997
1	2309	411	bi	1	2634	bi	1	2115	bi	1	1857	bi	1	1999
1	2310	382	bi	1	2635	bi	1	2116	bi	1	1858	bi	1	2000
1	2311	270	bi	1	2636	bi	1	2117	bi	1	1859	bi	1	2001
1	2312	248	-			-			-			bi	1	1544
1	2313	244	bi	1	2638	bi	1	2119	bi	1	1860	bi	1	2006
1	2314	76	bi	1	2639	bi	1	2120	bi	1	1861	bi	1	2007
1	2315	217	bi	1	2640	bi	1	2121	bi	1	1862	bi	1	2008
1	2316	271	uni	1	2421	uni	1	1786	uni	1	1677	uni	1	1782
1	2317	448	uni	1	2420	uni	1	1785	uni	1	1676	uni	1	1781
1	2318	380	-			-			-			-		
1	2319	133	-			-			-			-		
1	2320	818	uni	1	2825	uni	1	2370	uni	1	2044	uni	1	2198
1	2321	284	-			-			-			-		
1	2322	837	uni	1	2414	uni	1	1783	uni	1	1665	uni	1	1775
1	2323	489	uni	1	2409	uni	1	1773	uni	1	1685	uni	1	1770
1	2324	390	-			-			bi	1	1664	-		
1	2325	267	bi	1	2641	bi	1	2122	bi	1	1863	bi	1	2009
1	2326	272	bi	1	2642	bi	1	2123	bi	1	1864	bi	1	2010
1	2327	273	bi	1	2643	bi	1	2124	bi	1	1865	bi	1	2011
1	2328	229	bi	1	2644	bi	1	2125	bi	1	1866	bi	1	2012
1	2329	346	bi	1	2645	bi	1	2126	bi	1	1867	bi	1	2013
1	2330	31	-			-			-			-		
1	2331	105	bi	1	2646	bi	1	2127	bi	1	1868	bi	1	2015
1	2332	120	bi	1	2647	bi	1	2128	bi	1	1869	bi	1	2016
1	2333	396	bi	1	2648	bi	1	2129	bi	1	1870	bi	1	2017
1	2334	479	bi	1	2649	bi	1	2130	bi	1	1871	bi	1	2019
1	2335	234	bi	1	2650	bi	1	2131	bi	1	1872	bi	1	2020
1	2336	1652	bi	1	2651	bi	1	2132	bi	1	1873	bi	1	2021
1	2337	444	bi	1	2652	bi	1	2133	bi	1	1874	bi	1	2023
1	2338	184	bi	1	2653	bi	1	2134	bi	1	1875	bi	1	2024
1	2339	236	bi	1	2654	bi	1	2135	bi	1	1876	bi	1	2025
1	2340	120	-			-			-			-		
1	2341	179	-			-			-			-		
1	2342	75	-			-			-			-		
1	2343	343	uni	1	2305	bi	3	2989	-			uni	1	386
1	2344	682	-			-			-			-		
1	2345	835	uni	1	520	-			uni	1	780	-		
1	2346	73	uni	2	2927	-			-			-		
1	2347	130	-			-			-			-		
1	2348	75	-			-			-			-		
1	2349	329	uni	1	2185	-			uni	1	783	-		
1	2350	156	-			-			-			-		
1	2351	184	-			-			-			-		
1	2352	243	uni	1	133	uni	1	2667	uni	1	2275	uni	1	2491
1	2353	658	uni	1	1124	uni	1	595	uni	1	496	-		
1	2354	189	-			-			-			-		
1	2355	205	-			-			-			-		

1	2356	428	uni	1	2481	uni	1	1999	uni	1	772	-		
1	2357	142	-			-			-			-		
1	2358	172	-			-			-			-		
1	2359	99	-			-			-			-		
1	2360	89	-			-			-			-		
1	2361	503	uni	1	2202	uni	1	1720	uni	1	769	uni	1	1718
1	2362	123	-			-			-			-		
1	2363	111	-			-			-			-		
1	2364	108	-			-			-			-		
1	2365	80	-			-			-			-		
1	2366	51	-			-			-			-		
1	2367	75	-			-			-			-		
1	2368	135	-			-			-			-		
1	2369	109	-			-			-			-		
1	2370	84	-			-			-			-		
1	2371	60	-			-			-			-		
1	2372	127	bi	1	2771	bi	1	2315	-			bi	1	2143
1	2373	172	bi	1	1315	-			-			-		
1	2374	380	uni	1	530	uni	1	1841	bi	1	807	uni	1	1522
1	2375	79	bi	1	2656	bi	1	2137	bi	1	1878	bi	1	2027
1	2376	91	bi	1	2657	bi	1	2138	bi	1	1879	bi	1	2028
1	2377	210	bi	1	2658	bi	1	2139	bi	1	1880	bi	1	2029
1	2378	413	bi	1	2659	bi	1	2140	bi	1	1881	bi	1	2030
1	2379	341	bi	1	2660	bi	1	2141	bi	1	1882	bi	1	2031
1	2380	278	bi	1	2661	bi	1	2142	bi	1	1883	bi	1	2032
1	2381	358	bi	1	2662	bi	1	2143	bi	1	1884	bi	1	2033
1	2382	195	bi	1	2663	bi	1	2144	bi	1	1885	bi	1	2034
1	2383	506	bi	1	2665	bi	1	2145	bi	1	1886	bi	1	2035
1	2384	472	bi	1	2666	bi	1	2146	bi	1	1887	bi	1	2036
1	2385	1178	bi	1	2667	bi	1	2147	bi	1	1888	bi	1	2037
1	2386	473	bi	1	2668	bi	1	2148	bi	1	1889	bi	1	2038
1	2387	280	bi	1	2669	bi	1	2149	bi	1	1890	bi	1	2039
1	2388	153	bi	1	2670	bi	1	2150	bi	1	1891	bi	1	2040
1	2389	265	bi	1	2671	bi	1	2151	bi	1	1892	bi	1	2041
1	2390	252	bi	1	2672	bi	1	2152	bi	1	1893	bi	1	2042
1	2391	86	bi	1	2673	bi	1	2153	bi	1	1894	bi	1	2043
1	2392	196	bi	1	2674	bi	1	2154	bi	1	1895	bi	1	2044
1	2393	341	bi	1	2675	bi	1	2155	bi	1	1896	bi	1	2045
1	2394	383	bi	1	2676	bi	1	2156	bi	1	1897	bi	1	2046
1	2395	194	bi	1	2677	bi	1	2157	bi	1	1898	bi	1	2047
1	2396	858	bi	1	2678	bi	1	2158	bi	1	1899	bi	1	2048
1	2397	336	bi	1	2679	bi	1	2159	bi	1	1900	bi	1	2049
1	2398	120	bi	1	2680	bi	1	2160	bi	1	1901	bi	1	2050
1	2399	440	bi	1	2681	bi	1	2161	bi	1	1902	bi	1	2051
1	2400	127	bi	1	2682	bi	1	2162	bi	1	1903	bi	1	2052
1	2401	315	bi	1	2683	bi	1	2163	bi	1	1904	bi	1	2053
1	2402	38	bi	1	2684	-			-			bi	1	2054
1	2403	398	bi	1	2685	bi	1	2164	bi	1	1905	bi	1	2055
1	2404	421	bi	1	2686	bi	1	2165	bi	1	1906	bi	1	2056
1	2405	388	bi	1	2687	bi	1	2166	bi	1	1907	bi	1	2057
1	2406	458	bi	1	2688	bi	1	2167	bi	1	1908	bi	1	2058
1	2407	1002	bi	1	2689	bi	1	2168	bi	1	1909	bi	1	2059
1	2408	445	bi	1	2690	bi	1	2169	bi	1	1910	bi	1	2060
1	2409	213	bi	1	2691	bi	1	2170	bi	1	1911	bi	1	2061
1	2410	56	-			-			-			-		
1	2411	451	bi	1	2692	bi	1	2171	bi	1	1912	bi	1	2062
1	2412	231	bi	1	2693	bi	1	2172	bi	1	1913	bi	1	2063

1	2413	325	bi	1	2694	bi	1	2173	bi	1	1914	bi	1	2064
1	2414	182	bi	1	2695	bi	1	2174	bi	1	1915	bi	1	2065
1	2415	324	bi	1	2696	bi	1	2175	bi	1	1916	bi	1	2066
1	2416	348	bi	1	2697	bi	1	2176	bi	1	1917	bi	1	2067
1	2417	276	bi	1	2698	bi	1	2177	bi	1	1918	bi	1	2068
1	2418	623	bi	1	2699	bi	1	2178	bi	1	1919	bi	1	2069
1	2419	576	bi	1	2700	bi	1	2179	bi	1	1920	bi	1	2070
1	2420	226	bi	1	2701	bi	1	2180	bi	1	1921	bi	1	2071
1	2421	190	bi	1	2702	bi	1	2181	bi	1	1922	bi	1	2072
1	2422	482	bi	1	2703	bi	1	2182	bi	1	1923	bi	1	2073
1	2423	627	bi	1	2704	bi	1	2183	bi	1	1924	bi	1	2074
1	2424	281	bi	1	2705	bi	1	2184	bi	1	1925	bi	1	2075
1	2425	209	bi	1	2706	bi	1	2185	bi	1	1926	bi	1	2076
1	2426	390	bi	1	2707	bi	1	2186	bi	1	1927	bi	1	2077
1	2427	127	bi	1	2708	bi	1	2187	bi	1	1928	bi	1	2078
1	2428	59	bi	1	2709	bi	1	2188	bi	1	1929	bi	1	2079
1	2429	434	bi	1	2710	bi	1	2189	bi	1	1930	bi	1	2080
1	2430	79	bi	1	2711	bi	1	2190	bi	1	1931	bi	1	2081
1	2431	140	bi	1	2712	bi	1	2191	bi	1	1932	bi	1	2082
1	2432	469	bi	1	2713	bi	1	2192	bi	1	1933	bi	1	2083
1	2433	303	bi	1	2714	bi	1	2193	bi	1	1934	bi	1	2084
1	2434	519	bi	1	2715	bi	1	2194	bi	1	1935	bi	1	2085
1	2435	181	bi	1	2716	bi	1	2195	bi	1	1936	bi	1	2086
1	2436	177	bi	1	2717	bi	1	2196	bi	1	1937	bi	1	2087
1	2437	74	bi	1	2718	bi	1	2197	bi	1	1938	bi	1	2088
1	2438	240	bi	1	2719	bi	1	2198	bi	1	1939	bi	1	2089
1	2439	34	-			-			-			-		
1	2440	155	bi	1	2720	bi	1	2199	bi	1	1940	bi	1	2090
1	2441	790	bi	1	2721	bi	1	2200	bi	1	1941	bi	1	2091
1	2442	255	bi	1	2722	bi	1	2201	bi	1	1942	bi	1	2092
1	2443	33	-			-			-			-		
1	2444	79	bi	1	2723	bi	1	2202	bi	1	1943	bi	1	2093
1	2445	92	bi	1	2724	bi	1	2203	bi	1	1944	bi	1	2094
1	2446	95	bi	1	2725	bi	1	2204	bi	1	1945	bi	1	2095
1	2447	602	bi	1	2726	bi	1	2205	bi	1	1946	bi	1	2096
1	2448	32	-			-			-			-		
1	2449	276	bi	1	2727	bi	1	2206	bi	1	1947	bi	1	2097
1	2450	495	bi	1	2728	bi	1	2207	bi	1	1948	bi	1	2098
1	2451	137	bi	1	2729	bi	1	2208	bi	1	1949	bi	1	2099
1	2452	516	bi	1	2730	bi	1	2209	bi	1	1950	bi	1	2100
1	2453	237	bi	1	2731	bi	1	2210	bi	1	1951	bi	1	2101
1	2454	175	uni	1	2732	uni	1	2211	uni	1	1952	uni	1	2102
1	2455	57	bi	1	2732	bi	1	2211	bi	1	1952	bi	1	2102
1	2456	391	uni	1	2146	-			-			uni	1	1007
1	2457	542	bi	1	2733	bi	1	2212	bi	1	1953	bi	1	2103
1	2458	95	bi	1	2734	bi	1	2213	bi	1	1954	bi	1	2104
1	2459	59	bi	1	2735	bi	1	2214	bi	1	1955	bi	1	2105
1	2460	218	bi	1	2736	bi	1	2215	bi	1	1956	bi	1	2106
1	2461	217	bi	1	2737	bi	1	2216	bi	1	1957	bi	1	2107
1	2462	649	bi	1	2738	bi	1	2217	bi	1	1958	bi	1	2108
1	2463	211	bi	1	2739	bi	1	2218	bi	1	1959	bi	1	2109
1	2464	403	bi	1	2740	bi	1	2219	bi	1	1960	bi	1	2110
1	2465	570	bi	1	2741	bi	1	2220	bi	1	1961	bi	1	2113
1	2466	237	bi	1	2742	bi	1	2221	bi	1	1962	bi	1	2114
1	2467	301	bi	1	2743	bi	1	2222	bi	1	1963	bi	1	2115
1	2468	338	bi	1	2744	bi	1	2223	bi	1	1964	bi	1	2116
1	2469	383	bi	1	2745	bi	1	2224	bi	1	1965	bi	1	2117

1	2470	431	bi	1	2746	bi	1	2225	bi	1	1966	bi	1	2118
1	2471	228	-			-			-			-		
1	2472	358	bi	1	2748	bi	1	2227	bi	1	1968	bi	1	2120
1	2473	279	bi	1	2749	bi	1	2228	bi	1	1969	bi	1	2121
1	2474	269	bi	1	2750	bi	1	2229	bi	1	1970	bi	1	2122
1	2475	362	bi	1	2751	bi	1	2230	bi	1	1971	bi	1	2123
1	2476	181	bi	1	2752	bi	1	2231	bi	1	1972	bi	1	2124
1	2477	331	bi	1	2753	bi	1	2232	bi	1	1973	bi	1	2125
1	2478	258	bi	1	2754	bi	1	2233	bi	1	1974	bi	1	2126
1	2479	185	bi	1	2755	bi	1	2234	bi	1	1975	bi	1	2127
1	2480	212	bi	1	2756	bi	1	2235	bi	1	1976	bi	1	2128
1	2481	402	bi	1	2757	bi	1	2236	bi	1	1977	bi	1	2129
1	2482	169	bi	1	2758	bi	1	2237	bi	1	1978	bi	1	2130
1	2483	320	bi	1	2759	bi	1	2238	bi	1	1979	bi	1	2131
1	2484	374	bi	1	283	-			bi	1	2423	-		
1	2485	866	bi	1	2760	bi	1	2239	bi	1	1980	bi	1	2132
1	2486	215	bi	1	2761	bi	1	2240	bi	1	1981	bi	1	2133
1	2487	169	bi	1	2762	bi	1	2241	bi	1	1982	bi	1	2134
1	2488	282	bi	1	2763	bi	1	2242	bi	1	1983	bi	1	2135
1	2489	210	bi	1	2764	bi	1	2243	bi	1	1984	bi	1	2136
1	2490	454	bi	1	2765	bi	1	2244	bi	1	1985	bi	1	2137
1	3346	298	bi	1	2766	bi	1	2245	bi	1	1986	bi	1	2138
1	2491	266	bi	1	2767	bi	1	2246	bi	1	1987	bi	1	2139
1	2492	235	bi	1	2768	bi	1	2247	bi	1	1988	bi	1	2140
1	2493	193	bi	1	2769	bi	1	2248	bi	1	1989	bi	1	2141
1	2494	220	bi	1	2770	bi	1	2249	bi	1	1990	bi	1	2142
1	2495	604	bi	1	2772	bi	1	2251	bi	1	1991	bi	1	2144
1	2496	393	bi	1	2773	bi	1	2252	bi	1	1992	bi	1	2145
1	2497	221	bi	1	2774	bi	1	2253	bi	1	1993	bi	1	2146
1	2498	133	bi	1	2775	bi	1	2254	bi	1	1994	bi	1	2148
1	2499	337	bi	1	2776	bi	1	2255	bi	1	1995	bi	1	2149
1	2500	572	bi	1	2777	bi	1	2256	bi	1	1996	bi	1	2150
1	2501	238	bi	1	2778	bi	1	2320	bi	1	1997	bi	1	2151
1	2502	342	bi	1	2779	bi	1	2323	bi	1	1998	bi	1	2152
1	2503	259	bi	1	2780	bi	1	2324	bi	1	1999	bi	1	2153
1	2504	261	bi	1	2781	bi	1	2325	bi	1	2000	bi	1	2154
1	2505	123	bi	1	2782	bi	1	2326	bi	1	2001	bi	1	2155
1	3347	724	bi	1	2783	bi	1	2327	bi	1	2002	bi	1	2156
1	2506	308	bi	1	2784	bi	1	2328	bi	1	2003	bi	1	2157
1	2507	1066	bi	1	2785	bi	1	2329	bi	1	2004	bi	1	2158
1	2508	1046	bi	1	2786	bi	1	2330	bi	1	2005	bi	1	2159
1	2509	379	bi	1	2787	bi	1	2331	bi	1	2006	bi	1	2160
1	2510	209	bi	1	2788	bi	1	2332	bi	1	2007	bi	1	2161
1	2511	245	bi	1	2789	bi	1	2333	bi	1	2008	bi	1	2162
1	2512	89	bi	1	2790	bi	1	2334	bi	1	2009	bi	1	2163
1	2513	232	bi	1	2791	bi	1	2335	bi	1	2010	bi	1	2164
1	2514	99	bi	1	2792	bi	1	2336	bi	1	2011	bi	1	2165
1	2515	199	bi	1	2793	bi	1	2337	bi	1	2012	bi	1	2166
1	2516	227	bi	1	2794	bi	1	2338	bi	1	2013	bi	1	2167
1	2517	396	bi	1	2795	bi	1	2339	bi	1	2014	bi	1	2168
1	2518	32	-			-			-			-		
1	2519	274	bi	1	2796	bi	1	2340	bi	1	2015	bi	1	2169
1	2520	175	bi	1	2797	bi	1	2341	bi	1	2016	bi	1	2170
1	2521	144	bi	1	2798	bi	1	2342	bi	1	2017	bi	1	2171
1	2522	375	bi	1	2799	bi	1	2343	bi	1	2018	bi	1	2172
1	2523	395	bi	1	2800	bi	1	2344	bi	1	2019	bi	1	2173
1	2524	267	bi	1	2801	bi	1	2345	bi	1	2020	bi	1	2174

1	2525	457	bi	1	2802	bi	1	2346	bi	1	2021	bi	1	2175
1	2526	46	-			-			bi	1	1610	-		
1	2527	83	bi	1	2803	bi	1	2347	bi	1	2022	bi	1	2176
1	2528	1026	bi	1	2804	bi	1	2348	bi	1	2023	bi	1	2177
1	2529	500	bi	1	2805	bi	1	2349	bi	1	2024	bi	1	2178
1	2530	277	bi	1	2806	bi	1	2350	bi	1	2025	bi	1	2179
1	2531	33	-			-			-			-		
1	2532	256	-			-			-			-		
1	2533	123	bi	1	2808	bi	1	2352	bi	1	2027	bi	1	2181
1	2534	167	bi	1	2809	bi	1	2353	bi	1	2028	bi	1	2182
1	2535	230	bi	1	2810	bi	1	2355	bi	1	2029	bi	1	2183
1	2536	141	bi	1	2811	bi	1	2356	bi	1	2030	bi	1	2184
1	2537	499	bi	1	2812	bi	1	2357	bi	1	2031	bi	1	2185
1	2538	223	bi	1	2813	bi	1	2358	bi	1	2032	bi	1	2186
1	2539	291	bi	1	2814	bi	1	2359	bi	1	2033	bi	1	2187
1	2540	282	bi	1	2815	bi	1	2360	bi	1	2034	bi	1	2188
1	2541	368	bi	1	2816	bi	1	2361	bi	1	2035	bi	1	2189
1	2542	543	bi	1	2817	bi	1	2362	bi	1	2036	bi	1	2190
1	2543	241	bi	1	2818	bi	1	2363	bi	1	2037	bi	1	2191
1	2544	293	bi	1	2819	bi	1	2364	bi	1	2038	bi	1	2192
1	2545	182	bi	1	2820	bi	1	2365	bi	1	2039	bi	1	2193
1	2546	57	bi	1	2821	bi	1	2366	bi	1	2040	bi	1	2194
1	2547	51	bi	1	2822	bi	1	2367	bi	1	2041	bi	1	2195
1	2548	213	bi	1	2823	bi	1	2368	bi	1	2042	bi	1	2196
1	2549	301	bi	1	2825	bi	1	2370	bi	1	2044	bi	1	2198
1	2550	305	bi	1	2826	bi	1	2371	bi	1	2045	bi	1	2199
1	2551	252	bi	1	2827	bi	1	2372	bi	1	2046	bi	1	2200
1	2552	178	bi	1	2828	bi	1	2373	bi	1	2047	bi	1	2201
1	2553	561	bi	1	2830	bi	1	2375	bi	1	2049	bi	1	2203
1	2554	188	bi	1	2831	bi	1	2376	bi	1	2050	bi	1	2204
1	2555	230	bi	1	2832	bi	1	2377	bi	1	2051	bi	1	2205
1	2556	335	bi	1	2833	bi	1	2378	bi	1	2052	bi	1	2206
1	2557	247	bi	1	2834	bi	1	2379	bi	1	2053	bi	1	2207
1	2558	264	bi	1	2835	bi	1	2380	bi	1	2054	bi	1	2208
1	2559	354	bi	1	2836	bi	1	2381	bi	1	2055	bi	1	2209
1	2560	425	bi	1	2837	bi	1	2382	bi	1	2056	bi	1	2210
1	2561	79	bi	1	2838	bi	1	2383	bi	1	2057	bi	1	2211
1	2562	401	bi	1	2839	bi	1	2384	bi	1	2058	bi	1	2212
1	2563	507	bi	1	2840	bi	1	2385	bi	1	2059	bi	1	2213
1	2564	50	bi	1	2841	bi	1	2386	-			bi	1	2214
1	2565	699	bi	1	2842	bi	1	2387	bi	1	2060	bi	1	2215
1	2566	256	bi	1	2843	bi	1	2388	bi	1	2061	bi	1	2216
1	2567	728	bi	1	2845	bi	1	2390	bi	1	2063	bi	1	2218
1	2568	199	bi	1	2846	bi	1	2391	bi	1	2064	bi	1	2219
1	2569	373	bi	1	2847	bi	1	2392	bi	1	2065	bi	1	2220
1	2570	253	bi	1	2848	bi	1	2393	bi	1	2066	bi	1	2221
1	2571	221	bi	1	2849	bi	1	2394	bi	1	2067	bi	1	2222
1	2572	291	bi	1	2850	bi	1	2395	bi	1	2068	bi	1	2223
1	2573	116	bi	1	2851	bi	1	2396	bi	1	2069	bi	1	2224
1	2574	280	bi	1	2852	bi	1	2397	bi	1	2070	bi	1	2225
1	2575	317	bi	1	2853	bi	1	2398	bi	1	2071	bi	1	2226
1	2576	110	bi	1	2854	bi	1	2399	bi	1	2072	bi	1	2227
1	2577	213	bi	1	2855	bi	1	2400	bi	1	2073	bi	1	2228
1	2578	39	bi	1	2856	bi	1	2401	-			bi	1	2229
1	2579	199	bi	1	2857	bi	1	2402	bi	1	2074	bi	1	2230
1	2580	221	bi	1	2858	bi	1	2403	bi	1	2075	bi	1	2231
1	2581	218	bi	1	2859	bi	1	2404	bi	1	2076	bi	1	2232

1	2582	453	bi	1	2860	bi	1	2405	bi	1	2077	bi	1	2233
1	2583	188	bi	1	2861	bi	1	2406	bi	1	2078	bi	1	2234
1	2584	262	bi	1	2862	bi	1	2407	bi	1	2079	bi	1	2235
1	3348	314	bi	1	2863	bi	1	2408	bi	1	2080	bi	1	2236
1	2585	397	bi	1	2864	bi	1	2409	bi	1	2081	bi	1	2237
1	2586	119	bi	1	2865	bi	1	2410	bi	1	2082	bi	1	2238
1	2587	277	bi	1	2866	bi	1	2411	bi	1	2083	bi	1	2239
1	2588	212	bi	1	2867	bi	1	2412	bi	1	2084	bi	1	2240
1	2589	273	bi	1	2868	bi	1	2413	bi	1	2085	bi	1	2241
1	2590	177	bi	1	2869	bi	1	2414	bi	1	2086	bi	1	2242
1	2591	105	bi	1	2871	bi	1	2416	bi	1	2087	bi	1	2243
1	2592	587	bi	1	2872	bi	1	2417	bi	1	2088	bi	1	2244
1	2593	405	bi	1	2873	bi	1	2418	bi	1	2089	bi	1	2245
1	2594	331	bi	1	2874	bi	1	2419	bi	1	2090	bi	1	2246
1	2595	56	bi	1	2875	bi	1	2420	bi	1	2091	bi	1	2247
1	2596	613	bi	1	2876	bi	1	2421	bi	1	2092	bi	1	2248
1	2597	117	bi	1	2877	bi	1	2422	bi	1	2093	bi	1	2249
1	2598	133	bi	1	2878	bi	1	2423	bi	1	2094	bi	1	2250
1	2599	324	bi	1	2879	bi	1	2424	bi	1	2095	bi	1	2251
1	2600	73	bi	1	2880	bi	1	2425	bi	1	2096	bi	1	2252
1	2601	237	bi	1	2881	bi	1	2426	bi	1	2097	bi	1	2253
1	2602	181	bi	1	2882	bi	1	2427	bi	1	2098	bi	1	2254
1	2603	257	bi	1	2883	bi	1	2428	bi	1	2099	bi	1	2255
1	2604	192	bi	1	2884	bi	1	2429	bi	1	2100	bi	1	2256
1	2605	517	bi	1	2885	bi	1	2430	bi	1	2101	bi	1	2258
1	2606	355	bi	1	2886	bi	1	2431	bi	1	2102	bi	1	2259
1	2607	355	bi	1	2887	bi	1	2432	bi	1	2103	bi	1	2260
1	2608	66	bi	1	2888	bi	1	2433	bi	1	2104	bi	1	2261
1	2609	135	-			-			-			-		
1	2610	393	uni	1	1025	-			-			uni	1	424
1	2611	264	-			-			-			-		
1	2612	434	uni	1	2296	uni	1	2434	uni	1	518	bi	1	1806
1	2613	69	-			bi	1	2435	-			bi	1	2336
1	2614	78	-			uni	1	294	-			bi	1	2337
1	2615	46	uni	1	1365	bi	1	1659	-			uni	1	2338
1	2616	125	-			bi	1	1660	-			-		
1	2617	184	-			bi	1	1661	-			-		
1	3349	106	uni	1	2302	uni	1	1662	-			bi	1	2341
1	3350	106	uni	1	2303	uni	1	1663	-			bi	1	2342
1	2618	303	uni	1	2304	uni	1	1664	-			bi	1	2343
1	2619	932	uni	1	2305	uni	1	1665	-			bi	1	2344
1	2620	235	-			bi	1	2444	-			bi	1	2345
1	2621	768	-			bi	1	2445	-			bi	1	2346
1	2622	152	-			bi	1	2446	-			bi	1	2347
1	2623	215	-			bi	1	2447	-			bi	1	2348
1	2624	121	-			bi	1	2448	-			bi	1	2349
1	2625	113	-			bi	1	2449	-			bi	1	2350
1	2626	109	-			bi	1	2450	-			bi	1	2351
1	2627	94	-			bi	1	2451	-			bi	1	2352
1	2628	455	-			bi	1	2452	-			bi	1	2353
1	2629	191	-			bi	1	2453	-			bi	1	2354
1	2630	409	-			bi	1	2454	-			bi	1	2355
1	2631	576	-			bi	1	2455	-			bi	1	2356
1	2632	151	-			bi	1	2456	-			bi	1	2357
1	2633	125	-			bi	1	2457	-			-		
1	2634	136	-			bi	1	2459	-			bi	1	2358
1	2635	156	-			bi	1	2461	-			bi	1	2359

1	2636	78	-			-			-			uni	1	1517
1	2637	61	-			bi	3	2957	-			bi	1	1840
1	2638	40	-			-			-			-		
1	2639	33	-			-			-			-		
1	2640	273	-			-			-			-		
1	2641	69	-			bi	1	2468	-			bi	1	2364
1	2642	201	-			bi	1	2469	-			bi	1	2365
1	2643	157	-			bi	1	2470	-			bi	1	2366
1	2644	90	-			bi	1	2471	-			uni	1	1526
1	2645	284	uni	1	1668	bi	1	2472	uni	1	1018	bi	1	2367
1	2646	263	uni	1	1667	uni	1	1093	uni	1	1017	bi	1	2368
1	2647	268	-			bi	1	2474	-			bi	1	2369
1	2648	339	-			bi	1	2475	-			bi	1	2371
1	2649	36	bi	1	2334	bi	1	1697	-			bi	1	2372
1	2650	75	bi	1	2335	bi	1	2477	-			bi	1	2373
1	2651	100	uni	1	1324	bi	1	2478	-			bi	1	2374
1	2652	80	-			uni	1	2480	-			uni	1	1855
1	2653	90	-			bi	1	2481	-			-		
1	2654	105	-			-			-			-		
1	2655	43	-			-			-			-		
1	2656	59	-			-			-			bi	1	2377
1	2657	128	-			-			-			-		
1	2658	70	-			-			-			-		
1	2659	111	uni	1	1317	bi	1	2483	uni	1	1122	bi	1	2378
1	2660	115	-			bi	1	2484	-			bi	1	2379
1	2661	243	uni	1	100	bi	1	2485	uni	1	2243	uni	1	2460
1	2662	394	bi	1	1314	bi	1	2486	uni	1	1219	uni	1	1546
1	2663	50	bi	1	2889	bi	1	2487	bi	1	2105	bi	1	2262
1	2664	712	bi	1	2890	bi	1	2488	bi	1	2106	bi	1	2263
1	2665	646	bi	1	2891	bi	1	2489	bi	1	2107	bi	1	2264
1	2666	60	bi	1	2892	bi	1	2490	bi	1	2108	bi	1	2265
1	2667	475	bi	1	2896	bi	1	2494	bi	1	2112	bi	1	2269
1	2668	288	bi	1	2897	bi	1	2495	bi	1	2113	bi	1	2270
1	2669	194	bi	1	2898	bi	1	2496	bi	1	2114	bi	1	2271
1	2670	315	bi	1	2899	bi	1	2497	bi	1	2115	bi	1	2272
1	2671	273	bi	1	2900	bi	1	2498	bi	1	2116	bi	1	2273
1	2672	240	bi	1	2901	bi	1	2500	bi	1	2117	bi	1	2275
1	2673	393	bi	1	2902	bi	1	2501	bi	1	2118	bi	1	2276
1	2674	247	bi	1	2903	bi	1	2502	bi	1	2119	bi	1	2277
1	3351	50	bi	1	2904	bi	1	2503	bi	1	2120	bi	1	2278
1	2675	197	bi	1	2905	bi	1	2504	bi	1	2121	bi	1	2279
1	2676	220	bi	1	2906	bi	1	2505	bi	1	2122	bi	1	2280
1	2677	104	bi	1	2907	bi	1	2506	bi	1	2123	bi	1	2281
1	2678	370	bi	1	2908	bi	1	2507	bi	1	2124	bi	1	2282
1	2679	176	bi	1	2909	bi	1	2508	bi	1	2125	bi	1	2283
1	2680	263	bi	1	2910	bi	1	2509	bi	1	2126	bi	1	2284
1	2681	289	bi	1	2911	bi	1	2510	bi	1	2127	bi	1	2285
1	2682	457	bi	1	2912	bi	1	2511	bi	1	2128	bi	1	2286
1	2683	142	bi	1	2913	bi	1	2512	bi	1	2129	bi	1	2287
1	2684	163	bi	1	2914	bi	1	2513	bi	1	2130	bi	1	2288
1	2685	412	bi	1	2915	bi	1	2514	bi	1	2131	bi	1	2289
1	2686	246	bi	1	2916	bi	1	2515	bi	1	2132	bi	1	2290
1	2687	310	bi	1	2917	bi	1	2516	bi	1	2133	bi	1	2291
1	2688	319	bi	1	2918	bi	1	2517	bi	1	2134	bi	1	2292
1	2689	76	bi	1	2919	bi	1	2518	bi	1	2135	bi	1	2293
1	2690	322	bi	1	2920	bi	1	2519	bi	1	2136	bi	1	2294
1	2691	151	bi	1	2921	bi	1	2520	bi	1	2137	bi	1	2295

1	2692	77	bi	1	2922	bi	1	2521	bi	1	2138	bi	1	2296
1	2693	297	bi	1	2923	bi	1	2522	bi	1	2139	bi	1	2297
1	2694	408	bi	1	2924	bi	1	2523	bi	1	2140	bi	1	2298
1	2695	335	bi	1	2925	bi	1	2524	bi	1	2141	bi	1	2299
1	2696	85	-			-			-			-		
1	2697	121	bi	1	2	bi	1	2527	bi	1	2143	bi	1	2301
1	2698	123	bi	1	3	bi	1	2528	bi	1	2144	bi	1	2302
1	2699	389	bi	1	4	bi	1	2529	bi	1	2145	bi	1	2303
1	2700	69	bi	1	5	bi	1	2530	-			bi	1	2304
1	2701	197	bi	1	6	bi	1	2531	bi	1	2146	bi	1	2305
1	2702	337	bi	1	7	bi	1	2532	bi	1	2147	bi	1	2306
1	2703	39	-			-			-			-		
1	2704	321	bi	1	8	bi	1	2533	bi	1	2148	bi	1	2307
1	2705	518	bi	1	9	bi	1	2534	bi	1	2149	bi	1	2308
1	2706	427	bi	1	10	bi	1	2535	bi	1	2150	bi	1	2309
1	2707	430	bi	1	11	bi	1	2536	bi	1	2151	bi	1	2310
1	2708	273	bi	1	12	bi	1	2537	bi	1	2152	bi	1	2311
1	2709	292	bi	1	13	bi	1	2538	bi	1	2153	bi	1	2312
1	2710	351	bi	1	14	bi	1	2539	bi	1	2154	bi	1	2313
1	2711	313	bi	1	15	bi	1	2540	bi	1	2155	bi	1	2314
1	2712	104	bi	1	16	bi	1	2541	bi	1	2156	bi	1	2315
1	2713	222	bi	1	17	bi	1	2542	bi	1	2157	bi	1	2316
1	2714	211	bi	1	18	bi	1	2543	bi	1	2158	bi	1	2317
1	2715	368	bi	1	19	bi	1	2544	bi	1	2159	bi	1	2318
1	2716	244	bi	1	20	bi	1	2545	bi	1	2160	bi	1	2319
1	2717	160	bi	1	21	bi	1	2546	bi	1	2161	bi	1	2320
1	2718	462	bi	1	23	bi	1	2548	bi	1	2163	bi	1	2322
1	2719	218	bi	1	24	bi	1	2549	bi	1	2164	bi	1	2323
1	2720	383	bi	1	25	bi	1	2550	bi	1	2165	bi	1	2324
1	2721	164	bi	1	26	bi	1	2551	bi	1	2166	bi	1	2325
1	2722	590	bi	1	27	bi	1	2552	bi	1	2168	bi	1	2326
1	2723	576	bi	1	28	bi	1	2553	bi	1	2169	bi	1	2327
1	2724	35	-			-			-			-		
1	2725	129	bi	1	29	bi	1	2554	bi	1	2170	bi	1	2328
1	2726	71	bi	1	30	bi	1	2555	bi	1	2171	bi	1	2329
1	2727	562	bi	1	31	bi	1	2556	bi	1	2172	bi	1	2330
1	2728	67	bi	1	32	bi	1	2557	bi	1	2173	bi	1	2331
1	2729	233	bi	1	33	bi	1	2558	bi	1	2174	bi	1	2382
1	2730	219	bi	1	34	bi	1	2559	bi	1	2175	bi	1	2383
1	2731	441	bi	1	35	bi	1	2560	bi	1	2176	bi	1	2384
1	2732	855	bi	1	36	bi	1	2561	bi	1	2177	bi	1	2385
1	2733	200	bi	1	37	bi	1	2562	bi	1	2178	bi	1	2386
1	2734	881	bi	1	38	bi	1	2563	bi	1	2179	bi	1	2387
1	2735	163	bi	1	39	bi	1	2564	bi	1	2180	bi	1	2388
1	2736	216	bi	1	40	bi	1	2565	bi	1	2181	bi	1	2389
1	2737	405	bi	1	41	bi	1	2566	bi	1	2182	bi	1	2390
1	2738	438	bi	1	42	bi	1	2567	bi	1	2185	bi	1	2391
1	2739	113	-			-			-			-		
1	2740	70	uni	1	856	-			-			-		
1	2741	93	-			-			-			-		
1	2742	68	uni	1	1761	uni	1	1194	uni	1	1120	uni	1	1187
1	2743	191	-			-			-			-		
1	2744	66	-			-			-			-		
1	2745	186	-			-			-			-		
1	2746	178	-			-			-			-		
1	2747	161	-			-			-			-		
1	2748	47	-			-			-			-		

1	2749	48	-			-			-			-		
1	3352	60	-			-			-			-		
1	2750	137	-			-			-			-		
1	2751	795	-			-			-			-		
1	2752	83	-			-			-			-		
1	2753	98	-			-			-			-		
1	2754	222	-			-			-			-		
1	2755	83	-			-			-			-		
1	2756	106	-			-			-			-		
1	2757	317	uni	1	1316	uni	1	248	uni	1	1122	uni	1	1193
1	2758	382	bi	1	2550	uni	1	2486	uni	1	1219	bi	1	1546
1	2759	246	bi	1	43	bi	1	2568	bi	1	2186	bi	1	2392
1	2760	194	bi	1	44	bi	1	2569	bi	1	2187	bi	1	2393
1	2761	295	bi	1	45	bi	1	2570	bi	1	2188	bi	1	2394
1	2762	296	bi	1	46	bi	1	2571	bi	1	2189	bi	1	2395
1	2763	132	bi	1	47	bi	1	2572	bi	1	2190	bi	1	2396
1	2764	304	bi	1	48	bi	1	2573	bi	1	2191	bi	1	2397
1	2765	332	bi	1	49	bi	1	2574	bi	1	2192	bi	1	2398
1	2766	325	bi	1	50	bi	1	2575	bi	1	2193	bi	1	2399
1	2767	456	bi	1	51	bi	1	2576	bi	1	2194	bi	1	2400
1	2768	100	bi	1	52	bi	1	2577	bi	1	2195	bi	1	2401
1	2769	670	bi	1	53	bi	1	2578	bi	1	2196	bi	1	2402
1	2770	75	bi	1	54	bi	1	2579	bi	1	2197	bi	1	2403
1	2771	123	bi	1	55	bi	1	2580	bi	1	2198	bi	1	2404
1	2772	378	bi	1	56	bi	1	2581	bi	1	2199	bi	1	2405
1	2773	230	bi	1	57	bi	1	2582	bi	1	2200	bi	1	2406
1	2774	324	bi	1	58	bi	1	2583	bi	1	2201	bi	1	2407
1	2775	472	bi	1	59	bi	1	2584	bi	1	2202	bi	1	2408
1	2776	703	bi	1	60	bi	1	2585	bi	1	2203	bi	1	2409
1	2777	248	bi	1	1176	uni	1	2586	bi	1	2204	uni	1	2410
1	2778	368	bi	1	62	uni	1	660	bi	1	2205	bi	1	2411
1	2779	281	bi	1	63	bi	1	2588	bi	1	2206	bi	1	2412
1	2780	250	bi	1	64	bi	1	2589	bi	1	2207	bi	1	2413
1	2781	165	bi	1	65	bi	1	2590	bi	1	2208	bi	1	2414
1	2782	134	bi	1	66	bi	1	2591	bi	1	2209	bi	1	2415
1	2783	938	bi	1	67	bi	1	2592	bi	1	2210	bi	1	2416
1	2784	176	bi	1	68	bi	1	2593	bi	1	2211	bi	1	2417
1	2785	483	bi	1	69	bi	1	2594	bi	1	2212	bi	1	2418
1	2786	222	bi	1	70	bi	1	2595	bi	1	2213	bi	1	2419
1	2787	403	bi	1	71	bi	1	2596	bi	1	2214	bi	1	2420
1	2788	225	bi	1	72	bi	1	2597	bi	1	2215	bi	1	2421
1	2789	389	bi	1	73	bi	1	2598	bi	1	2216	bi	1	2422
1	2790	100	bi	1	74	bi	1	2599	bi	1	2217	bi	1	2423
1	2791	550	bi	1	75	bi	1	2600	bi	1	2218	bi	1	2424
1	2792	105	bi	1	76	bi	1	2601	bi	1	2219	bi	1	2425
1	2793	87	bi	1	77	bi	1	2602	bi	1	2220	bi	1	2426
1	2794	420	bi	1	78	bi	1	2603	bi	1	2221	bi	1	2427
1	2795	403	bi	1	79	bi	1	2604	bi	1	2222	bi	1	2428
1	2796	542	bi	1	80	bi	1	2605	bi	1	2223	bi	1	2429
1	2797	262	bi	1	81	bi	1	2606	bi	1	2224	bi	1	2430
1	2798	408	bi	1	82	bi	1	2607	bi	1	2225	bi	1	2431
1	2799	455	bi	1	83	bi	1	2608	bi	1	2226	bi	1	2432
1	2800	499	bi	1	84	bi	1	2609	bi	1	2227	bi	1	2433
1	2801	172	bi	1	85	bi	1	2610	bi	1	2228	bi	1	2434
1	2802	151	bi	1	86	bi	1	2611	bi	1	2229	bi	1	2435
1	2803	231	bi	1	87	bi	1	2612	bi	1	2230	bi	1	2437
1	2804	540	bi	1	88	bi	1	2613	bi	1	2231	bi	1	2438

1	2805	356	bi	1	89	bi	1	2614	bi	1	2232	bi	1	2439
1	2806	219	bi	1	90	bi	1	2615	bi	1	2233	-		
1	2807	358	bi	1	91	-			bi	1	2234	bi	1	2445
1	2808	552	bi	1	92	bi	1	2622	bi	1	2235	bi	1	2452
1	2809	289	bi	1	93	bi	1	2623	bi	1	2236	bi	1	2453
1	2810	520	bi	1	94	bi	1	2624	bi	1	2237	bi	1	2454
1	2811	475	bi	1	95	bi	1	2625	bi	1	2238	bi	1	2455
1	2812	394	bi	1	96	bi	1	2626	bi	1	2239	bi	1	2456
1	2813	483	bi	1	97	bi	1	2627	bi	1	2240	bi	1	2457
1	2814	851	bi	1	98	bi	1	2628	bi	1	2241	bi	1	2458
1	2815	421	bi	1	99	bi	1	2629	bi	1	2242	bi	1	2459
1	2816	128	bi	1	100	bi	1	2630	bi	1	2243	bi	1	2460
1	3353	247	bi	1	102	bi	1	2631	bi	1	2244	bi	1	2461
1	2817	594	bi	1	103	bi	1	2632	bi	1	2245	bi	1	2462
1	2818	309	bi	1	104	bi	1	2633	bi	1	2246	bi	1	2463
1	2819	155	bi	1	105	bi	1	2634	bi	1	2247	bi	1	2464
1	2820	170	bi	1	106	bi	1	2635	bi	1	2248	bi	1	2465
1	2821	463	bi	1	107	bi	1	2636	bi	1	2249	bi	1	2466
1	2822	1373	bi	1	109	bi	1	2637	bi	1	2250	bi	1	2467
1	2823	39	bi	1	110	bi	1	2638	bi	1	2251	bi	1	2468
1	2824	160	bi	1	111	bi	1	2639	bi	1	2252	bi	1	2469
1	2825	433	bi	1	112	bi	1	2640	bi	1	2253	bi	1	2470
1	2826	207	bi	1	113	bi	1	2641	bi	1	2254	bi	1	2471
1	2827	273	uni	1	2260	bi	1	2642	uni	1	1567	uni	1	1676
1	2828	257	uni	1	2261	bi	1	2643	uni	1	1568	uni	1	1677
1	2829	158	uni	1	2262	bi	1	2644	uni	1	1569	uni	1	337
1	2830	248	-			bi	1	2645	-			-		
1	2831	137	uni	1	278	bi	1	2646	uni	1	532	uni	1	2632
1	2832	244	uni	1	2030	bi	1	2647	uni	1	1380	uni	1	1464
1	2833	152	bi	1	114	bi	1	2648	bi	1	2255	bi	1	2472
1	2834	105	bi	1	115	bi	1	2649	bi	1	2256	bi	1	2473
1	2835	359	bi	1	116	bi	1	2650	bi	1	2257	bi	1	2474
1	2836	114	bi	1	117	bi	1	2651	bi	1	2258	bi	1	2475
1	2837	37	-			-			-			-		
1	2838	552	bi	1	118	bi	1	2652	bi	1	2259	bi	1	2476
1	2839	273	bi	1	119	bi	1	2653	bi	1	2260	bi	1	2477
1	2840	258	bi	1	120	bi	1	2654	bi	1	2261	bi	1	2478
1	2841	384	bi	1	121	bi	1	2655	bi	1	2263	bi	1	2479
1	2842	159	bi	1	122	bi	1	2656	bi	1	2264	bi	1	2480
1	2843	146	bi	1	123	bi	1	2657	bi	1	2265	bi	1	2481
1	2844	365	bi	1	124	bi	1	2658	bi	1	2266	bi	1	2482
1	2845	263	bi	1	125	bi	1	2659	bi	1	2267	bi	1	2483
1	2846	251	bi	1	126	bi	1	2660	bi	1	2268	bi	1	2484
1	2847	184	bi	1	127	bi	1	2661	bi	1	2269	bi	1	2485
1	2848	256	bi	1	128	bi	1	2662	bi	1	2270	bi	1	2486
1	2849	121	bi	1	129	bi	1	2663	bi	1	2271	bi	1	2487
1	2850	192	bi	1	130	bi	1	2664	bi	1	2272	bi	1	2488
1	2851	209	bi	1	131	bi	1	2665	bi	1	2273	bi	1	2489
1	2852	262	bi	1	132	bi	1	2666	bi	1	2274	bi	1	2490
1	2853	245	bi	1	133	bi	1	2667	bi	1	2275	bi	1	2491
1	2854	92	bi	1	134	bi	1	2668	bi	1	2276	bi	1	2492
1	2855	157	bi	1	135	bi	1	2669	bi	1	2277	bi	1	2493
1	2856	190	bi	1	136	bi	1	2670	bi	1	2278	bi	1	2494
1	2857	483	bi	1	137	bi	1	2671	bi	1	2279	bi	1	2495
1	2858	170	bi	1	138	bi	1	2672	bi	1	2280	bi	1	2496
1	2859	287	bi	1	139	bi	1	2673	bi	1	2281	bi	1	2497
1	2860	92	-			-			-			-		

1	2861	705	bi	1	140	bi	1	2674	bi	1	2282	bi	1	2498
1	2862	90	bi	1	141	bi	1	2675	bi	1	2283	bi	1	2499
1	2863	188	bi	1	142	bi	1	2676	bi	1	2284	bi	1	2500
1	2864	259	bi	1	143	bi	1	2677	bi	1	2285	bi	1	2501
1	2865	218	bi	1	144	bi	1	2678	bi	1	2286	bi	1	2502
1	2866	268	bi	1	145	bi	1	2679	bi	1	2287	bi	1	2503
1	2867	204	bi	1	146	bi	1	2680	bi	1	2288	bi	1	2504
1	2868	35	-			-			-			-		
1	2869	183	bi	1	147	bi	1	2681	bi	1	2289	bi	1	2505
1	2870	179	bi	1	148	bi	1	2682	bi	1	2290	bi	1	2506
1	2871	1295	bi	1	149	bi	1	2684	bi	1	2291	bi	1	2507
1	2872	1427	bi	1	150	bi	1	2836	bi	1	2292	bi	1	2508
1	2873	121	bi	1	151	bi	1	2686	bi	1	2293	bi	1	2509
1	2874	204	bi	1	152	bi	1	2687	bi	1	2294	bi	1	2510
1	2875	186	bi	1	153	bi	1	2688	bi	1	2295	bi	1	2511
1	2876	407	bi	1	154	bi	1	2689	bi	1	2296	bi	1	2512
1	2877	548	bi	1	155	bi	1	2690	bi	1	2297	bi	1	2513
1	2878	318	bi	1	156	bi	1	2691	bi	1	2298	bi	1	2514
1	2879	252	bi	1	157	bi	1	2692	bi	1	2299	bi	1	2515
1	2880	322	bi	1	158	bi	1	2693	bi	1	2300	bi	1	2516
1	2881	316	bi	1	159	bi	1	2694	bi	1	2301	bi	1	2517
1	2882	151	bi	1	160	bi	1	2695	bi	1	2302	bi	1	2518
1	2883	46	uni	1	305	uni	1	2833	bi	1	2303	bi	1	2519
1	2884	61	-			-			uni	1	2303	uni	1	2519
1	2885	757	bi	1	161	bi	1	2696	bi	1	2304	bi	1	2520
1	2886	167	bi	1	162	bi	1	2697	bi	1	2305	bi	1	2521
1	2887	299	bi	1	163	bi	1	2698	bi	1	2306	bi	1	2522
1	2888	133	bi	1	164	bi	1	2699	bi	1	2307	bi	1	2523
1	2889	162	bi	1	165	bi	1	2700	bi	1	2308	bi	1	2524
1	2890	443	bi	1	166	bi	1	2701	bi	1	2309	bi	1	2525
1	2891	276	bi	1	167	bi	1	2702	bi	1	2310	bi	1	2526
1	2892	1193	bi	1	168	bi	1	2703	bi	1	2311	bi	1	2527
1	2893	231	bi	1	170	bi	1	2705	bi	1	2312	bi	1	2528
1	2894	173	-			-			-			-		
1	2895	391	uni	1	2146	-			-			uni	1	1007
1	2896	127	-			-			-			-		
1	2897	89	-			-			-			-		
1	2898	398	-			-			-			-		
1	2899	242	uni	1	2392	uni	1	1756	uni	1	1645	uni	1	1753
1	2900	72	-			-			-			-		
1	2901	594	bi	1	171	bi	1	2707	bi	1	2313	bi	1	2529
1	2902	308	bi	1	172	bi	1	2708	bi	1	2314	bi	1	2530
1	2903	321	bi	1	173	bi	1	2709	bi	1	2315	bi	1	2531
1	2904	315	bi	1	174	bi	1	2710	bi	1	2316	bi	1	2532
1	2905	336	bi	1	175	bi	1	2711	bi	1	2317	bi	1	2533
1	2906	80	bi	1	176	bi	1	2713	bi	1	2318	bi	1	2535
1	2907	334	bi	1	177	bi	1	2714	bi	1	2319	bi	1	2536
1	2908	679	bi	1	178	bi	1	2715	bi	1	2320	bi	1	2537
1	2909	559	bi	1	179	bi	1	2716	bi	1	2321	bi	1	2538
1	2910	121	bi	1	180	bi	1	2717	bi	1	2322	bi	1	2539
1	2911	63	bi	1	185	bi	1	2722	bi	1	2327	bi	1	2545
1	2912	215	bi	1	186	bi	1	2723	bi	1	2328	bi	1	2546
1	2913	218	bi	1	187	bi	1	2724	bi	1	2329	bi	1	2547
1	2914	298	bi	1	188	bi	1	2725	bi	1	2330	bi	1	2548
1	2915	719	bi	1	189	bi	1	2726	bi	1	2331	bi	1	2549
1	2916	250	bi	1	190	bi	1	2727	bi	1	2332	bi	1	2550
1	2917	460	bi	1	191	bi	1	2728	bi	1	2333	bi	1	2551

1	2918	314	bi	1	192	bi	1	2729	bi	1	2334	bi	1	2552
1	3354	109	bi	1	193	bi	1	2730	bi	1	2335	bi	1	2553
1	2919	811	bi	1	194	bi	1	2731	bi	1	2336	bi	1	2554
1	2920	100	bi	1	195	bi	1	2732	bi	1	2337	bi	1	2555
1	2921	205	bi	1	196	bi	1	2733	bi	1	2338	bi	1	2556
1	2922	494	bi	1	197	bi	1	2734	bi	1	2339	bi	1	2557
1	2923	102	bi	1	198	bi	1	2735	bi	1	2340	bi	1	2558
1	2924	295	bi	1	199	bi	1	2736	bi	1	2341	bi	1	2559
1	2925	73	bi	1	200	bi	1	2737	bi	1	2342	bi	1	2560
1	2926	157	bi	1	202	bi	1	2738	bi	1	2343	bi	1	2561
1	2927	218	bi	1	203	bi	1	2739	bi	1	2344	bi	1	2562
1	2928	358	bi	1	204	bi	1	2740	bi	1	2345	bi	1	2563
1	2929	151	bi	1	205	bi	1	2741	bi	1	2346	bi	1	2564
1	2930	169	bi	1	206	bi	1	2742	bi	1	2347	bi	1	2565
1	2931	291	bi	1	207	bi	1	2743	bi	1	2348	bi	1	2566
1	2932	279	bi	1	208	bi	1	2744	bi	1	2349	bi	1	2567
1	2933	369	bi	1	209	bi	1	2745	bi	1	2350	bi	1	2568
1	2934	334	bi	1	210	bi	1	2746	bi	1	2351	bi	1	2569
1	2935	300	bi	1	211	bi	1	2747	bi	1	2352	bi	1	2570
1	2936	296	bi	1	212	bi	1	2748	bi	1	2353	bi	1	2571
1	2937	151	-			-			-			-		
1	2938	239	bi	1	213	bi	1	2749	bi	1	2354	uni	1	2585
1	2939	193	bi	1	214	bi	1	2750	bi	1	2355	bi	1	2574
1	2940	289	bi	1	221	bi	1	2757	bi	1	2362	bi	1	2576
1	2941	435	bi	1	222	bi	1	2758	bi	1	2363	bi	1	2577
1	2942	423	bi	1	223	bi	1	2759	bi	1	2364	bi	1	2578
1	2943	150	bi	1	224	bi	1	2760	bi	1	2365	bi	1	2579
1	2944	239	uni	1	226	uni	1	2761	uni	1	2367	uni	1	2581
1	2945	237	bi	1	226	bi	1	2761	bi	1	2367	bi	1	2581
1	2946	233	bi	1	225	uni	1	2761	bi	1	2366	uni	1	2581
1	2947	238	bi	1	227	bi	1	2762	bi	1	2368	bi	1	2582
1	2948	779	bi	1	228	bi	1	2764	bi	1	2369	bi	1	2583
1	2949	217	bi	1	229	bi	1	2765	bi	1	2370	bi	1	2584
1	2950	148	bi	1	230	uni	1	2749	bi	1	2371	bi	1	2585
1	2951	134	-			-			-			-		
1	2952	324	bi	1	233	bi	1	2767	bi	1	2373	bi	1	2587
1	2953	146	bi	1	234	bi	1	2768	bi	1	2374	bi	1	2588
1	2954	185	bi	1	235	bi	1	2769	bi	1	2375	bi	1	2589
1	2955	711	bi	1	236	bi	1	2770	bi	1	2376	bi	1	2590
1	2956	859	bi	1	237	bi	1	2771	bi	1	2377	bi	1	2591
1	2957	124	bi	1	238	bi	1	2772	bi	1	2378	bi	1	2592
1	2958	266	bi	1	239	bi	1	2773	bi	1	2379	bi	1	2593
1	2959	519	bi	1	240	bi	1	2774	bi	1	2380	bi	1	2594
1	2960	349	bi	1	242	bi	1	2775	bi	1	2381	bi	1	2595
1	2961	415	bi	1	243	bi	1	2776	bi	1	2382	bi	1	2596
1	2962	402	bi	1	244	bi	1	2777	bi	1	2383	bi	1	2597
1	2963	207	bi	1	245	bi	1	2778	bi	1	2384	bi	1	2598
1	2964	142	bi	1	246	bi	1	2779	bi	1	2385	bi	1	2599
1	2965	128	bi	1	247	bi	1	2780	bi	1	2386	bi	1	2600
1	2966	264	bi	1	248	bi	1	2781	bi	1	2387	bi	1	2601
1	2967	379	bi	1	249	bi	1	2782	bi	1	2388	bi	1	2602
1	2968	295	bi	1	250	bi	1	2783	bi	1	2389	bi	1	2603
1	2969	166	bi	1	251	bi	1	2784	bi	1	2390	bi	1	2604
1	2970	484	bi	1	252	bi	1	2785	bi	1	2392	bi	1	2605
1	2971	162	bi	1	253	bi	1	2786	bi	1	2393	bi	1	2606
1	2972	359	bi	1	254	bi	1	2787	bi	1	2394	bi	1	2607
1	2973	253	bi	1	255	bi	1	2788	bi	1	2395	bi	1	2608

1	2974	267	bi	1	256	bi	1	2789	bi	1	2396	bi	1	2609
1	2975	245	bi	1	257	bi	1	2790	bi	1	2397	bi	1	2610
1	2976	130	uni	1	2782	uni	1	2326	uni	1	2001	uni	1	2155
1	2977	1555	bi	1	259	bi	1	2792	bi	1	2399	bi	1	2612
1	2978	45	-			-			-			-		
1	2979	338	bi	1	260	bi	1	2794	bi	1	2400	bi	1	2614
1	2980	182	bi	1	261	bi	1	2795	bi	1	2401	bi	1	2615
1	2981	228	bi	1	262	bi	1	2796	bi	1	2402	bi	1	2616
1	2982	140	bi	1	263	bi	1	2797	bi	1	2403	bi	1	2617
1	2983	243	bi	1	264	bi	1	2798	bi	1	2404	bi	1	2618
1	2984	590	bi	1	265	bi	1	2799	bi	1	2405	bi	1	2619
1	2985	273	bi	1	266	bi	1	2800	bi	1	2406	bi	1	2620
1	2986	224	bi	1	267	bi	1	2801	bi	1	2407	bi	1	2621
1	2987	241	bi	1	268	bi	1	2802	bi	1	2408	bi	1	2622
1	2988	131	bi	1	269	bi	1	2803	bi	1	2409	bi	1	2623
1	2989	90	bi	1	270	bi	1	2804	bi	1	2410	bi	1	2624
1	2990	50	bi	1	271	bi	1	2805	bi	1	2411	bi	1	2625
1	2991	325	bi	1	272	bi	1	2806	bi	1	2412	bi	1	2626
1	2992	47	bi	1	273	bi	1	2807	bi	1	2413	bi	1	2627
1	2993	512	bi	1	274	bi	1	2808	bi	1	2414	bi	1	2628
1	2994	325	bi	1	275	bi	1	2809	bi	1	2415	bi	1	2629
1	2995	257	bi	1	276	bi	1	2810	bi	1	2416	bi	1	2630
1	2996	312	bi	1	277	bi	1	2811	bi	1	2417	bi	1	2631
1	2997	140	bi	1	278	bi	1	2812	bi	1	2418	bi	1	2632
1	2998	164	bi	1	279	bi	1	2813	bi	1	2419	bi	1	2633
1	2999	272	bi	1	280	bi	1	2814	bi	1	2420	bi	1	2634
1	3000	276	bi	1	281	bi	1	2815	bi	1	2421	bi	1	2635
1	3001	404	uni	1	282	uni	1	2816	uni	1	2422	bi	1	2636
1	3002	391	uni	1	2146	-			-			uni	1	1007
1	3003	427	bi	1	282	bi	1	2816	bi	1	2422	bi	1	2637
1	3004	965	-			-			-			-		
1	3005	139	-			-			-			-		
1	3006	1211	-			-			-			-		
1	3007	77	-			bi	1	920	-			-		
1	3008	103	-			-			-			-		
1	3009	255	bi	1	2549	uni	1	694	uni	1	584	uni	1	698
1	3010	110	-			-			-			-		
1	3011	168	-			-			-			-		
1	3012	278	-			-			-			-		
1	3013	91	-			uni	1	2481	-			-		
1	3355	190	-			-			-			-		
1	3356	125	uni	1	1314	-			-			uni	1	1546
1	3014	131	bi	1	288	bi	1	2819	bi	1	2427	bi	1	2644
1	3357	117	bi	1	289	bi	1	2820	bi	1	2428	bi	1	2645
1	3015	36	-			-			-			-		
1	3016	156	bi	1	290	bi	1	2821	bi	1	2429	bi	1	2646
1	3017	240	bi	1	291	bi	1	2822	bi	1	2430	bi	1	2647
1	3018	507	bi	1	292	bi	1	2823	bi	1	2431	bi	1	2648
1	3019	231	bi	1	293	bi	1	2824	bi	1	2432	bi	1	2649
1	3020	1218	bi	1	294	bi	1	2825	bi	1	2433	bi	1	2650
1	3021	1208	bi	1	295	bi	1	2826	bi	1	2434	bi	1	2651
1	3022	72	bi	1	299	bi	1	2829	bi	1	2437	bi	1	2655
1	3023	331	bi	1	300	bi	1	2830	bi	1	2438	bi	1	2656
1	3024	211	-			-			-			-		
1	3025	432	-			-			-			-		
1	3026	45	-			-			-			-		
1	3027	31	-			-			-			-		

1	3028	539	bi	1	302	bi	1	2832	bi	1	2440	bi	1	2658
1	3029	66	bi	1	231	-			-			bi	1	2659
1	3030	1253	uni	1	306	uni	1	2834	uni	1	2442	uni	1	274
1	3031	48	bi	1	305	bi	1	2833	uni	1	2303	bi	1	2660
1	3032	646	bi	1	306	bi	1	2834	bi	1	2442	bi	1	2661
1	3033	312	uni	1	307	uni	1	2835	uni	1	2443	uni	1	2661
1	3034	1266	bi	1	307	uni	1	2685	bi	1	2443	uni	1	2508
1	3035	121	bi	1	309	bi	1	2837	bi	1	2445	bi	1	2662
1	3036	316	bi	1	310	bi	1	2838	bi	1	2446	bi	1	2663
1	3037	356	bi	1	311	bi	1	2839	bi	1	2447	bi	1	2664
1	3038	310	bi	1	312	bi	1	2840	bi	1	2448	bi	1	2665
1	3039	649	bi	1	313	bi	1	2841	bi	1	2449	bi	1	2666
1	3040	141	bi	1	314	bi	1	2842	bi	1	2450	bi	1	2667
1	3041	186	bi	1	315	bi	1	2843	bi	1	2451	bi	1	2668
1	3042	329	bi	1	316	bi	1	2844	bi	1	2452	bi	1	2669
1	3043	77	bi	1	317	bi	1	2845	bi	1	2436	bi	1	2653
1	3044	122	bi	1	318	bi	1	2846	bi	1	2435	bi	1	2652
1	3045	28	-			-			-			-		
1	3046	34	-			-			-			-		
1	3047	264	bi	1	319	bi	1	2847	bi	1	2453	bi	1	2670
1	3048	198	bi	1	320	bi	1	2848	bi	1	2454	bi	1	2671
1	3049	187	bi	1	321	bi	1	2849	bi	1	2455	bi	1	2672
1	3050	162	bi	1	322	bi	1	2850	bi	1	2456	bi	1	2673
1	3051	121	bi	1	323	bi	1	2851	bi	1	2457	bi	1	2674
1	3052	450	bi	1	324	bi	1	2862	bi	1	2458	bi	1	2676
1	3053	133	bi	1	325	bi	1	2863	bi	1	2459	bi	1	2677
1	3054	380	bi	1	326	-			bi	1	2460	-		
1	3055	158	uni	1	327	-			uni	1	2461	-		
1	3056	64	bi	1	327	-			bi	1	2461	-		
1	3057	518	bi	1	328	bi	1	2864	bi	1	2462	bi	1	2678
1	3058	365	bi	1	329	bi	1	2865	bi	1	2463	bi	1	2679
1	3059	423	bi	1	330	bi	1	2866	bi	1	2464	bi	1	2680
1	3060	521	bi	1	331	bi	1	2867	bi	1	2465	bi	1	2681
1	3061	416	bi	1	332	bi	1	2868	bi	1	2466	bi	1	2682
1	3062	307	bi	1	333	bi	1	2869	bi	1	2467	bi	1	2683
1	3063	337	bi	1	334	bi	1	2870	bi	1	2468	bi	1	2684
1	3064	832	bi	1	335	bi	1	2871	bi	1	2469	bi	1	2685
1	3065	156	bi	1	336	bi	1	2872	bi	1	2470	bi	1	2686
1	3066	396	bi	1	337	bi	1	2873	bi	1	2471	bi	1	2687
1	3067	436	bi	1	338	bi	1	2874	bi	1	2472	bi	1	2688
1	3068	286	bi	1	339	bi	1	2875	bi	1	2473	bi	1	2689
1	3069	28	-			-			-			-		
1	3070	230	bi	1	341	bi	1	2877	bi	1	2474	bi	1	2690
1	3071	394	bi	1	342	bi	1	2878	bi	1	2475	bi	1	2691
1	3072	424	bi	1	343	bi	1	2879	bi	1	2476	bi	1	2692
1	3073	494	bi	1	344	bi	1	2880	bi	1	2477	bi	1	2694
1	3074	561	bi	1	345	bi	1	2881	bi	1	2478	bi	1	2695
1	3075	230	bi	1	346	bi	1	2882	bi	1	2479	bi	1	2696
1	3076	372	bi	1	347	bi	1	2883	bi	1	2480	bi	1	2697
1	3077	62	bi	1	348	bi	1	2884	bi	1	2481	bi	1	2698
1	3078	297	bi	1	349	bi	1	2885	bi	1	2482	bi	1	2699
1	3079	254	bi	1	350	bi	1	2886	bi	1	2483	bi	1	2700
1	3080	239	bi	1	351	bi	1	2887	bi	1	2484	bi	1	2701
1	3081	329	bi	1	352	bi	1	2888	bi	1	2485	bi	1	2702
1	3082	49	-			-			bi	1	2486	-		
1	3083	591	bi	1	353	bi	1	2890	bi	1	2487	bi	1	2703
1	3084	226	bi	1	354	bi	1	2891	bi	1	2488	bi	1	2704

1	3085	119	bi	1	355	bi	1	2892	bi	1	2489	bi	1	2705
1	3086	332	bi	1	356	bi	1	2893	bi	1	2490	bi	1	2706
1	3087	185	bi	1	357	bi	1	2894	bi	1	2491	bi	1	2707
1	3088	162	bi	1	358	bi	1	2895	bi	1	2492	bi	1	2708
1	3089	621	bi	1	359	bi	1	2896	bi	1	2493	bi	1	2709
1	3090	267	bi	1	360	bi	1	2897	bi	1	2494	bi	1	2710
1	3091	633	bi	1	361	bi	1	2898	bi	1	2495	bi	1	2711
1	3092	466	bi	1	362	bi	1	2899	bi	1	2496	bi	1	2712
1	3093	35	-			-			-			-		
1	3094	1745	bi	1	363	bi	1	2900	bi	1	2497	bi	1	2713
1	3095	290	bi	1	365	bi	1	2902	bi	1	2499	bi	1	2715
1	3096	391	bi	1	366	bi	1	2903	bi	1	2500	bi	1	2716
1	3097	466	bi	1	367	bi	1	2904	bi	1	2501	bi	1	2717
1	3098	179	bi	1	368	bi	1	2905	bi	1	2502	bi	1	2718
1	3099	471	bi	1	369	bi	1	2906	bi	1	2503	bi	1	2719
1	3100	296	bi	1	370	bi	1	2907	bi	1	2504	bi	1	2720
1	3101	103	bi	1	371	bi	1	2908	bi	1	2505	bi	1	2721
1	3102	337	bi	1	372	bi	1	2909	bi	1	2506	bi	1	2722
1	3103	45	bi	1	373	bi	1	2910	bi	1	2507	bi	1	2723
1	3104	362	bi	1	374	bi	1	2911	bi	1	2508	bi	1	2724
1	3105	134	bi	1	375	bi	1	2912	bi	1	2509	bi	1	2725
1	3106	121	bi	1	376	bi	1	2913	bi	1	2510	bi	1	2726
1	3107	466	bi	1	377	bi	1	2914	bi	1	2511	bi	1	2727
1	3108	232	bi	1	378	bi	1	2915	bi	1	2512	bi	1	2728
1	3109	241	bi	1	379	bi	1	2916	bi	1	2513	bi	1	2729
1	3110	259	bi	1	380	bi	1	2924	bi	1	2514	bi	1	2730
1	3111	276	bi	1	381	bi	1	2925	bi	1	2515	bi	1	2731
1	3112	119	bi	1	382	bi	1	2926	bi	1	2516	bi	1	2732
1	3113	45	bi	1	383	bi	1	2927	bi	1	2517	bi	1	2733
2	3114	337	uni	4	2995	bi	1	1891	-			-		
2	3115	389	uni	3	2935	-			-			-		
2	3116	544	uni	3	2934	uni	1	2652	uni	1	2259	uni	1	2476
2	3117	320	bi	4	2992	uni	1	69	uni	1	63	uni	1	69
2	3118	23	-			-			-			-		
2	3119	229	bi	1	2520	uni	2	2938	-			bi	1	372
2	3120	246	uni	1	1275	bi	2	2933	uni	1	644	uni	1	761
2	3121	229	uni	1	2520	uni	2	2938	-			uni	1	372
2	3122	108	uni	1	667	uni	1	300	uni	1	246	uni	1	247
2	3123	296	-			-			-			-		
2	3124	229	uni	1	2520	uni	2	2938	-			uni	1	372
2	3125	164	-			-			-			-		
2	3126	45	-			-			-			-		
2	3127	230	uni	1	798	uni	2	2938	-			uni	1	410
2	3128	81	uni	1	304	uni	1	2833	-			uni	1	2586
2	3129	159	uni	4	3017	uni	1	8	uni	1	8	uni	1	8
2	3358	317	uni	3	2964	-			-			uni	1	373
2	3130	252	uni	3	2965	-			-			-		
2	3131	89	-			-			-			-		
2	3132	163	uni	4	3021	-			-			-		
2	3133	262	uni	4	3022	-			-			-		
2	3134	54	-			-			-			-		
2	3135	80	-			-			-			-		
2	3136	48	-			-			-			-		
2	3137	738	uni	4	3024	-			-			-		
2	3138	782	uni	4	3025	-			-			-		
2	3139	114	bi	4	3027	-			-			-		
2	3140	831	uni	4	3028	-			uni	1	780	-		

2	3141	153	bi	4	3030	uni	1	1948	uni	1	8	uni	1	8
2	3142	134	bi	4	3031	-			-			-		
2	3143	92	bi	4	3032	-			-			-		
2	3144	345	bi	4	3033	-			-			-		
2	3145	105	bi	4	3034	-			-			-		
2	3146	139	bi	4	3035	-			-			-		
2	3147	206	bi	4	3036	-			-			-		
2	3148	424	bi	4	3037	uni	1	1563	uni	1	1486	uni	1	193
2	3149	287	bi	4	3038	-			-			uni	1	395
2	3150	293	bi	4	3039	-			-			uni	1	394
2	3151	87	bi	4	3040	-			-			-		
2	3152	129	bi	4	3041	-			-			-		
2	3153	118	bi	4	3042	-			-			uni	1	396
2	3154	1297	uni	4	3043	-			-			uni	1	397
2	3155	112	bi	4	3044	bi	3	2953	-			-		
2	3156	31	-			-			-			-		
2	3157	62	-			-			-			-		
2	3158	891	bi	4	3045	-			-			-		
2	3159	102	bi	3	2938	uni	1	1852	-			-		
2	3160	119	-			bi	3	2945	-			-		
2	3161	229	uni	1	2520	uni	2	2938	-			uni	1	372
2	3162	47	-			bi	2	2941	-			-		
2	3163	497	-			bi	2	2928	-			-		
2	3164	391	uni	1	2146	-			-			uni	1	1007
2	3165	131	-			uni	1	188	uni	1	185	uni	1	185
2	3166	480	-			-			-			-		
2	3167	391	uni	1	2146	-			-			uni	1	1007
2	3168	229	uni	1	2520	uni	2	2938	-			uni	1	372
2	3169	115	-			bi	3	3007	-			-		
2	3170	90	-			-			-			-		
2	3171	644	uni	1	1921	uni	1	1353	uni	1	1279	uni	1	1355
2	3172	480	uni	1	1923	uni	1	1355	uni	1	1281	uni	1	1357
2	3173	321	uni	1	1924	uni	1	1356	uni	1	1282	uni	1	1358
2	3174	121	uni	1	1188	uni	1	1873	uni	1	563	uni	1	677
2	3175	88	-			-			-			-		
2	3176	199	bi	4	3005	bi	3	3008	uni	1	140	bi	1	456
2	3177	96	bi	1	854	bi	3	3009	-			-		
2	3178	83	bi	3	2983	-			-			-		
2	3179	101	bi	3	2984	-			-			-		
2	3180	443	bi	3	2985	uni	3	3017	uni	1	832	uni	1	905
2	3181	117	-			-			-			uni	1	2332
2	3182	61	uni	4	3003	-			-			-		
2	3183	154	-			-			-			-		
2	3359	97	uni	3	2987	-			-			-		
2	3184	124	-			-			-			-		
2	3185	282	bi	1	822	uni	3	3019	uni	1	2483	bi	1	435
3	3186	439	bi	3	2934	uni	1	2652	uni	1	2259	uni	1	2476
3	3187	229	uni	1	2520	uni	2	2938	-			uni	1	372
3	3360	140	-			-			-			-		
3	3188	391	uni	1	2146	-			-			uni	1	1007
3	3189	613	-			-			-			-		
3	3190	352	uni	1	1476	uni	3	3017	uni	1	832	uni	1	905
3	3191	229	uni	1	2520	uni	2	2938	-			uni	1	372
3	3192	101	-			-			-			-		
3	3193	199	uni	4	3005	uni	3	3008	uni	1	140	uni	1	456
3	3194	95	uni	4	3004	uni	3	3009	-			-		
3	3195	70	bi	3	2982	-			-			-		

3	3196	75	-			-			-			-		
3	3197	52	-			-			-			-		
3	3198	84	-			-			-			-		
3	3199	439	uni	3	2985	uni	3	3017	uni	1	832	uni	1	905
3	3200	169	uni	4	2999	bi	1	2258	uni	1	833	bi	1	2332
3	3201	138	-			bi	3	3018	-			-		
3	3202	274	uni	1	822	bi	3	3019	uni	1	2483	uni	1	367
3	3203	502	-			bi	3	2942	-			-		
4	3204	337	bi	4	2995	uni	1	1891	-			-		
4	3205	386	bi	3	2935	-			-			-		
4	3206	530	uni	3	2934	uni	1	727	uni	1	616	uni	1	732
4	3207	314	bi	3	2936	uni	1	69	uni	1	63	-		
4	3208	26	-			-			-			-		
4	3209	127	bi	3	2937	bi	1	1996	-			-		
4	3210	63	uni	3	2938	-			-			-		
4	3211	38	uni	3	2938	-			-			-		
4	3212	892	uni	4	3045	-			-			-		
4	3213	1306	bi	3	2942	-			-			bi	1	397
4	3361	119	bi	3	2943	-			-			bi	1	396
4	3214	286	bi	3	2944	-			-			bi	1	395
4	3215	300	bi	3	2945	-			-			bi	1	394
4	3216	145	bi	3	2946	-			-			bi	1	393
4	3217	76	bi	3	2947	-			-			bi	1	392
4	3218	99	bi	3	2948	-			-			bi	1	391
4	3219	267	bi	3	2949	-			-			bi	1	389
4	3220	118	bi	3	2950	-			-			bi	1	388
4	3221	746	bi	3	2951	-			-			bi	1	387
4	3222	872	bi	3	2952	uni	1	1980	uni	1	1486	bi	1	386
4	3223	209	bi	3	2953	-			-			bi	1	385
4	3224	85	bi	3	2954	-			-			bi	1	384
4	3225	203	bi	3	2955	-			-			bi	1	383
4	3226	163	bi	3	2956	-			-			bi	1	382
4	3227	611	bi	3	2957	bi	1	1969	-			bi	1	381
4	3228	720	bi	3	2958	bi	3	2982	-			bi	1	378
4	3229	91	bi	3	2959	-			-			bi	1	377
4	3230	119	bi	3	2960	-			-			bi	1	376
4	3231	562	bi	3	2961	-			-			bi	1	375
4	3232	110	bi	3	2962	-			-			bi	1	374
4	3233	56	bi	3	2963	-			-			-		
4	3234	448	bi	3	2964	-			-			bi	1	373
4	3235	259	bi	3	2965	-			-			-		
4	3236	183	bi	3	2966	-			-			-		
4	3237	58	bi	3	2967	-			-			-		
4	3238	62	bi	3	2968	-			-			-		
4	3239	150	bi	3	2969	-			-			-		
4	3240	67	bi	3	2970	-			-			-		
4	3241	77	bi	3	2971	bi	3	2995	-			-		
4	3242	165	bi	3	2972	-			-			-		
4	3243	151	bi	3	2973	bi	1	1948	uni	1	8	uni	1	8
4	3244	51	bi	3	2974	-			-			-		
4	3245	193	bi	3	2975	uni	1	1946	-			-		
4	3246	110	-			-			-			-		
4	3247	487	bi	3	2976	uni	1	1749	uni	1	1638	uni	1	1746
4	3248	82	-			-			-			bi	1	2675
4	3249	447	uni	1	2409	uni	1	1773	uni	1	1665	uni	1	1770
4	3250	701	uni	4	3008	uni	1	1478	uni	1	1403	uni	1	1487
4	3251	430	-			-			-			-		

4	3252	391	uni	1	2146	-			-			uni	1	1007
4	3253	125	-			-			-			-		
4	3254	135	uni	1	1083	uni	1	555	uni	1	455	uni	1	576
4	3255	165	-			-			-			-		
4	3256	75	-			-			-			-		
4	3257	112	uni	1	1095	uni	1	566	uni	1	467	uni	1	587
4	3258	202	uni	1	843	bi	2	2930	uni	1	140	uni	1	456
4	3259	631	bi	1	901	-			-			-		
4	3260	439	uni	3	2985	bi	3	3017	uni	1	832	uni	1	905
4	3261	169	bi	4	2999	uni	1	2258	uni	1	833	uni	1	2332
4	3262	68	-			-			-			-		
4	3263	99	bi	3	2987	-			-			-		
4	3264	261	bi	4	2997	uni	1	2886	uni	1	2483	uni	1	2700
4	3265	119	bi	4	2996	-			-			-		
